# Biophysical Survey of Small-Molecule β-Catenin Inhibitors: A Cautionary Tale

**DOI:** 10.1021/acs.jmedchem.2c00228

**Published:** 2022-05-17

**Authors:** Michael A. McCoy, Dominique Spicer, Neil Wells, Kurt Hoogewijs, Marc Fiedler, Matthias G. J. Baud

**Affiliations:** † School of Chemistry, University of Southampton, Southampton SO17 1BJ, U.K.; ‡ National University of Ireland, University Road, Galway H91 TK33, Ireland; § Medical Research Council, Laboratory of Molecular Biology, Francis Crick Avenue, Cambridge CB2 0QH, U.K.

## Abstract

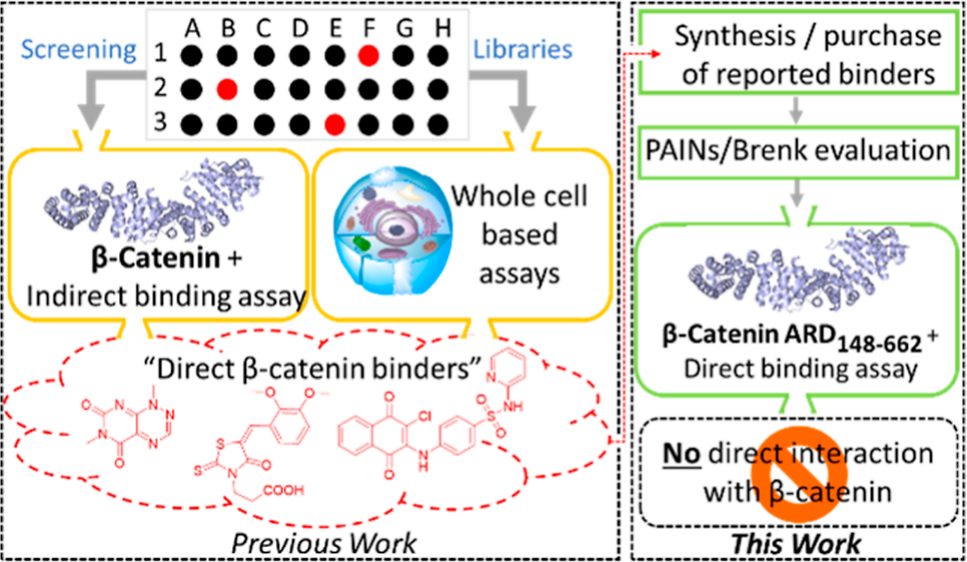

The canonical Wingless-related integration site signaling pathway plays a critical role in human physiology, and its dysregulation can lead to an array of diseases. β-Catenin is a multifunctional protein within this pathway and an attractive yet challenging therapeutic target, most notably in oncology. This has stimulated the search for potent small-molecule inhibitors binding directly to the β-catenin surface to inhibit its protein–protein interactions and downstream signaling. Here, we provide an account of the claimed (and some putative) small-molecule ligands of β-catenin from the literature. Through in silico analysis, we show that most of these molecules contain promiscuous chemical substructures notorious for interfering with screening assays. Finally, and in line with this analysis, we demonstrate using orthogonal biophysical techniques that none of the examined small molecules bind at the surface of β-catenin. While shedding doubts on their reported mode of action, this study also reaffirms β-catenin as a prominent target in drug discovery.

## Introduction

### Canonical Wnt/β-Catenin Signaling Pathway

**Figure 1:**
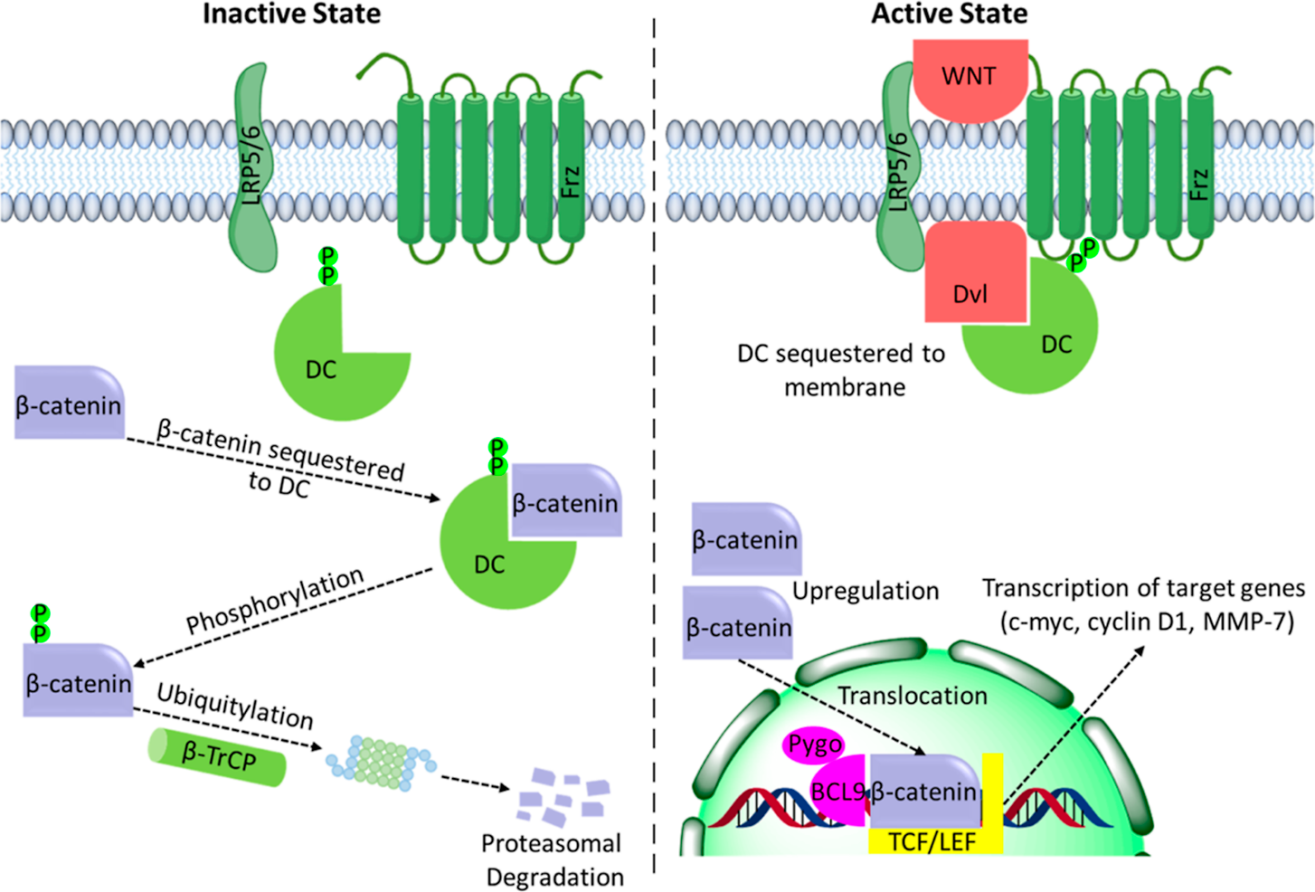
Schematic of the Wnt signaling pathway in both the inactive (left) and active (right) state.

The canonical Wingless-related integration site (Wnt)/β-catenin signaling pathway plays a pivotal role in human physiology and is highly evolutionary conserved in metazoans. At the crux of the canonical pathway, the protein β-catenin fulfills multiple roles underlying signal transduction and structural maintenance and is critical for embryonic development and adult tissue homeostasis.^1^ In the absence of a Wnt stimulus, the cellular concentration of β-catenin is tightly regulated and maintained at a low level.^2^ This regulatory mechanism is mediated by the action of a cytoplasmic multiprotein complex that associates with β-catenin and induces its phosphorylation/ubiquitination, which leads to its proteasomal degradation (Figure 1 left). The scaffolding proteins Axin and adenomatous polyposis coli (APC) form the core of this destruction complex (DC) and can recruit the casein kinases 1α, β, and δ (CK1) and glycogen synthase kinase 3 (GSK3). A sequential process involves the binding of β-catenin to Axin, followed by the phosphorylation of β-catenin at Ser45 by CK1, and further phosphorylation at Ser33, Ser37, and Ser41 by GSK3. This series of phosphorylations promotes the transfer of β-catenin from Axin to APC and allows Axin to bind a new molecule of β-catenin. The APC:β-catenin complex exposes the N-terminally phosphorylated part of β-catenin to the ubiquitin ligase β-transducin repeat-containing protein (β-TrCP), resulting in β-catenin ubiquitination and degradation by the proteasome. However, the binding of a Wnt-protein ligand to the extracellular domain of the Frizzled (Fz) transmembrane receptor induces the recruitment of the phosphoprotein Dishevelled (Dvl) to the membrane, assisted by several co-receptors (e.g., LRP-5/6, Ryk, and ROR2). This induces the disruption of the DC through its segregation to the plasma membrane, allowing β-catenin accumulation in the cytoplasm. β-Catenin translocates to the nucleus and associates with transcriptional regulators, resulting in the upregulation of Wnt target genes (e.g., c-myc, axin2, cyclin D1, MMP-7, and CD44) and cell proliferation (Figure 1, right).

These oncogenes, and others, play key roles in the inception and progression of many cancer types, giving rise to the various hallmarks of cancer.^3−6^ Overall, the degradative regulation mechanism of β-catenin offers a fast cellular response mechanism at the post-translational level upon extracellular stimuli.^7^

### Pharmacological Modulation

The importance of this regulatory mechanism is underscored by the observation that the dysregulation of Wnt/β-catenin signaling contributes to the development of an array of diseases^8^ including osteoarthritis,^9^ neurodegenerative diseases,^10,11^ inflammatory bowel disease,^12^ type II diabetes,^13^ and multiple cancer types.^14−22^ In particular, an intimate relationship between the misregulation of Wnt signaling and numerous subtypes of colorectal cancer (CRC) is now well established.^1^ However, current therapies targeting CRC remain largely toxic and ineffective, making CRC the second leading cause of cancer-related death in developed countries.^23^ Crucially, the hyperactivity of the signal transducer protein β-catenin (encoded by the CTNNB1 gene) is a hallmark of CRC, and the modulation of its activity has been proposed as a promising strategy for developing novel classes of anticancer drugs.^21,24−28^ The small-molecule inhibition of diverse components of canonical Wnt signaling has recently been summarized in an excellent review.^29^

**Figure 2:**
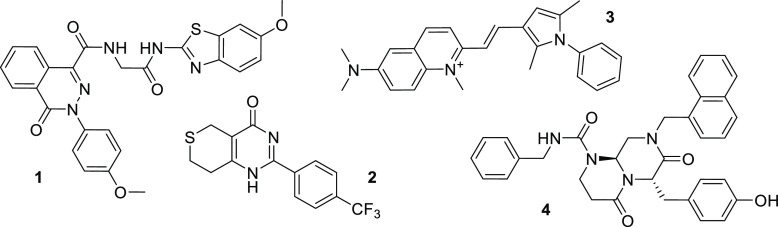
Structures of representative Wnt signaling inhibitors acting upstream (**1**–**3**) and downstream (**4**) of the DC.

In its active form, the signal transducer protein β-catenin acts as a transcription factor but also plays a key role in cell adhesion through its interaction with the cadherins. Despite the current paradigm, the relationships linking these complex and intricate protein–protein interaction (PPI) cascades and the resulting phenotypes are only partly understood. Thus, there is enormous interest in developing small molecules that target β-catenin-mediated signaling as chemical probes to dissect important molecular recognition events controlling these PPIs and their phenotypic effects, but also as proof-of-principle lead compounds toward first-in-class therapeutics in disease, notably CRC. Importantly, the spatial and temporal controls that are afforded by small molecules compared to more traditional gene knockouts or RNAi present significant advantages for studying the complex molecular physiology of this system. To date, limited success has been achieved by indirectly targeting β-catenin. For example, the porcupine inhibitor IWP-1 (**1**, Figure 2) prevents signaling through inhibiting the palmitoylation and secretion of Wnt ligands.^30^ XAV939 (**2**) stabilizes Axin through tankyrase inhibition, resulting in enhanced β-catenin degradation.^31^ Pyrvinium (**3**) promotes β-catenin phosphorylation through casein kinase activation^32^ and ICG-001 (**4**) binds to the cyclic adenosine monophosphate response element binding protein binding protein (CBP) and prevents its interaction with β-catenin in the nucleus.^33^ Although all the abovementioned strategies aim to downregulate the activity of β-catenin, their indirect inhibitory effects on β-catenin activity raise important concerns regarding their selectivity and mode of action as their molecular targets also interact with numerous other transcription factors from other signaling pathways. Additionally, oncogenic mutations leading to the inactivation of the DC (e.g., truncated APC) or the desensitization of β-catenin to the DC invariably result in β-catenin stabilization^34^ and will lead to limited response to molecular agents targeting the DC or upstream components.^1^

### Direct Targeting of β-Catenin

**Figure 3:**
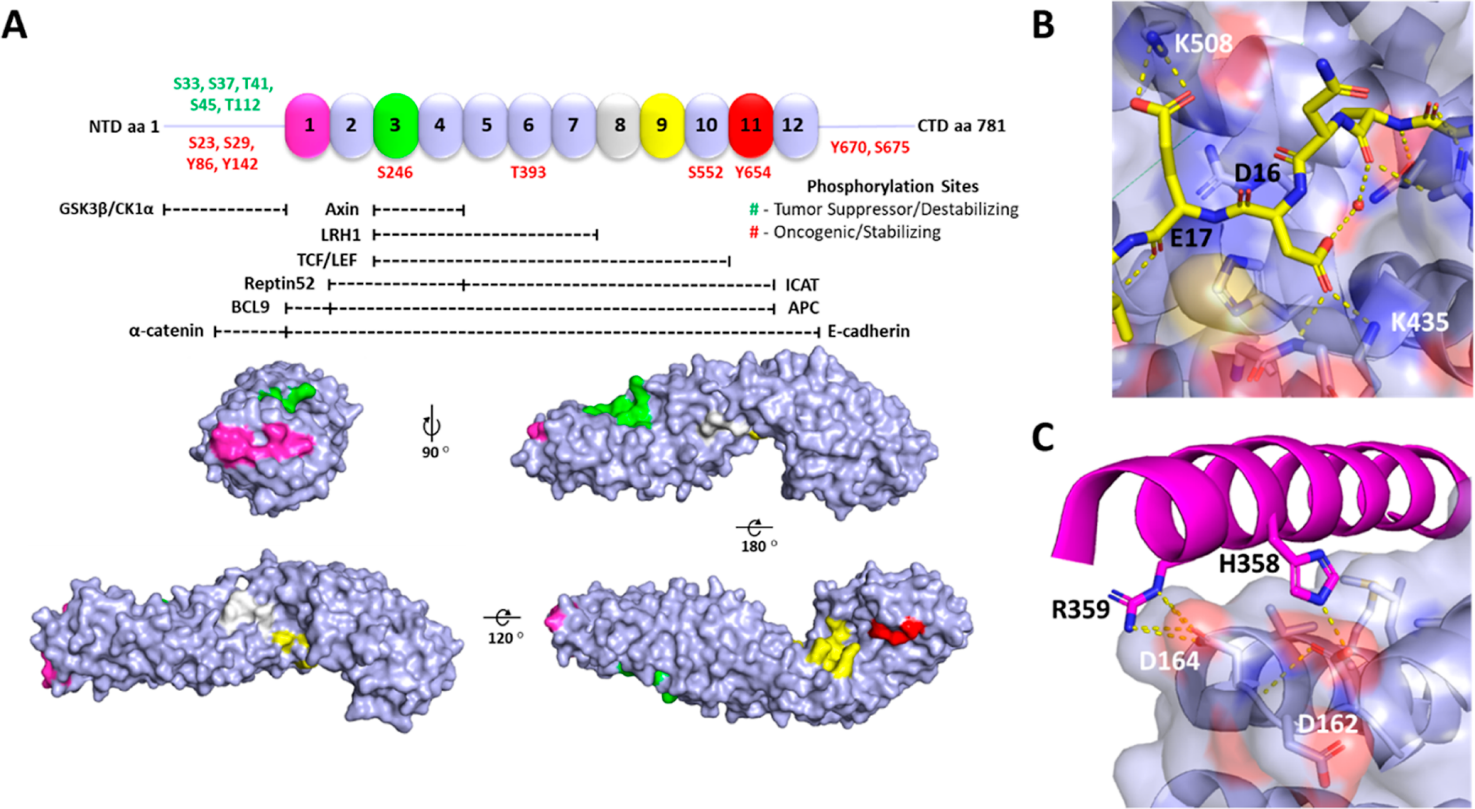
Structure of β-catenin with PPI interfaces and hotspots, and key PPI partners. (A) Schematic of β-catenin (aa 001–781) with the unstructured N-terminal domain (001–126), ARD (127–682), C-terminal domain (683–781), and phosphorylation sites (oncogenic—red or tumor suppressing—green). The 12 repeats are numbered. The five main interaction hotspots are highlighted in pink, green, white, yellow, and red.^42,45^ The color coding is consistent with that of Figure 1. (B) Shows key interactions between the TCF/LEF hotspot and the unstructured region of the TCF protein (yellow sticks). (C) Shows key interactions between the BCL9 hotspot and the BCL9 protein (purple cartoon/sticks). β-Catenin is shown with surface representation and key hotspot residues are shown in white; residues contributing to PPI in TCF4 and BCL9 are shown in black.

The direct targeting of β-catenin would conversely be highly desirable for the development of more specific chemical probes/drugs, as β-catenin is a specific component of the Wnt/β-catenin signaling pathway. Notably, the targeted/selective PPI disruption between β-catenin and its nuclear co-regulators downstream of the DC has attracted significant interest in inhibiting Wnt signaling and cancer cell proliferation.^25,28^ Full-length β-catenin is an 85 kDa protein, with residues 127–682 forming the structured “armadillo repeat domain” (ARD, 56 kDa, Figure 3A), while the C- and N-termini of the ARD are largely unstructured.^35^ ARD engages in complex interactions with a variety of cellular co-regulators within the plasma membrane (e.g., *E*-cadherin^36^), the cytoplasm (e.g., APC and^37,38^ Axin^39^), and the nucleus (e.g., Tcf4^40,41^). Its distinctive structure resembling that of a super helix is composed of 12 repeat units, which are themselves composed of three short α-helical segments (Figure 3A). Importantly, binding hotspots at the surface of the ARD (Figure 3A–C) have been identified.^42−44^ The binding pocket formed by N426, N430, K435, R469, H470, R474, and K508 (ARD-repeat 9, yellow, Figure 3A,B) has been well documented as it engages in key interactions with D16/E17 of Tcf4/Lef1 and contributes the most to the binding affinity (*K*_d_ ∼ 10 nM).^25,27,42,43,45,46^ The α-helical groove in ARD-repeat 1 (pink, Figure 3A,C) is engaged by the α-helical HD2 domain of BCL9 (aa, 349–377).^47^ This region can be divided into two key sites, the acidic knob (D162, E163, and D164) and the hydrophobic groove (L156, L159, V167, A171, M174, and L178).^48,49^ These hotspot residues contribute disproportionately to the binding affinity of Tcf4/Lef1 and BCL9 and, ultimately, the oncogenicity of these PPIs.

The presence of these well-characterized interaction hotspots at the surface of the ARD suggests that the modulation of these PPIs should be possible through the devising of selective and potent disruptors (e.g., small molecules) competing with β-catenin partners. However, several hotspots engage in overlapping and mutually exclusive PPIs with several high affinity co-regulators having either oncogenic (e.g., Tcf4, CBP, and BCL9) or tumor suppressor functions (e.g., AXIN, APC, and *E*-cadherin). In particular, the inhibition of the β-catenin/Tcf4 interaction has raised concerns as Tcf4 shares its binding surface with *E*-cadherin. The β-catenin/E-cadherin complex is a key regulator of cell adhesion and membrane integrity. This in part explains why most screening campaigns targeting β-catenin have relied on functional assays rather than focusing on specific binding and PPI inhibition. However, Cong and co-workers showed that the soluble, oncogenic, β-catenin pool can be effectively and selectively targeted for degradation without affecting the cadherin-bound pool, mainly through exploiting the lower affinity of the β-catenin/TCF4 PPI.^50^ This is interesting as it suggests that the increased sensitivity of oncogenic β-catenin can be exploited toward small-molecule inhibition without affecting cell adhesion. The development of potent chemical probes directly targeting β-catenin will be crucial not only for their potential therapeutic applications but equally importantly for the accurate functional elucidation of these PPIs in human biology. Overall, the high affinity of β-catenin PPIs (e.g., nanomolar *K*_d_), large interacting surfaces (e.g., >4000 Å^2^), and the high degree of overlap of the binding surfaces makes the development of small-molecule inhibitors a daunting task.

### Small Molecules

**Figure 4:**
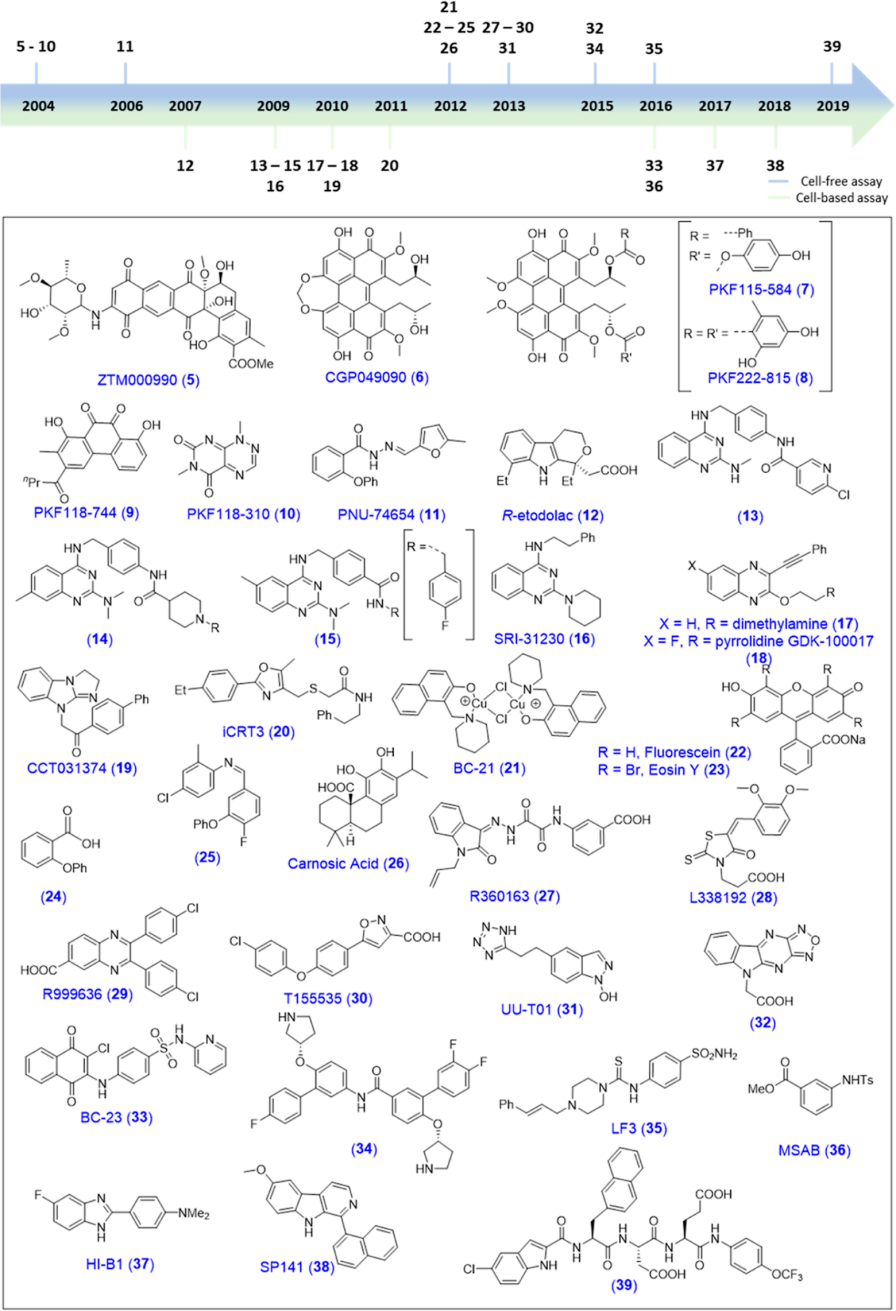
Structures of small molecules proposed to target Wnt/β-catenin signaling via direct engagement/binding of β-catenin. The year they were reported is indicated on the abovementioned timeline. Above arrow: discovered in cell free assays and below arrow: discovered by whole cell, functional assays.

Despite such challenges, a small number of compounds targeting the surface of β-catenin have been reported (Figure 4) and are currently under scrutiny.^24,25,27,46^ However, the majority of these compounds originate from functional screening assays and unambiguous evidence of target engagement through biophysical binding assessment and structural methods has not been reported. As a result, and despite the overwhelming case for direct β-catenin targeting in Wnt signaling modulation, relatively few such compounds have been validated and, to date, there is no clinically approved small-molecule β-catenin inhibitor. As will be discussed further in this article, a particular caveat in this area is the lack of biophysical and structural data associated with reported β-catenin inhibitors. As of 2020, not a single crystal structure of the ARD bound to a small molecule has been reported. Only recently, while writing this manuscript, has the first crystal structure of a truncated ARD (repeats 1–4) bound to a low affinity small molecule fragment been reported (vide infra). This lack of structural and biophysical knowledge has generally hampered further hit-to-lead optimization and perhaps provides an explanation for why none of these reported hits from high-throughput screening campaigns have advanced to clinical evaluation.

### Aim of This Study

The aim of this study is twofold. (i) In the first part, we are providing a comprehensive review of the small-molecule β-catenin inhibitors reported in the literature up to 2020, focusing on their proposed mode of action and potency, along with available biophysical/biological data on target engagement and their binding mode to β-catenin; and (ii) in the second part, we are investigating the molecular interactions mediating β-catenin inhibition by reported (and some putative) β-catenin ligands/inhibitors from the literature using orthogonal in vitro biophysical techniques, namely isothermal titration calorimetry (ITC) and differential scanning fluorimetry (DSF). ITC, in particular, is label free and considered one of the gold standards for studying molecular interactions in the solution phase. It allows direct and accurate measurement of the binding enthalpy (Δ*H*), equilibrium constant (*K*_a_ or *K*_d_), and stoichiometry (*n*) of a molecular association event, from which the Gibbs free energy (Δ*G*) and entropy of binding (Δ*S*) can also be deduced. Hence, both the binding affinity and its thermodynamic components can be accessed from a single experiment. Access to the thermodynamic fingerprints of β-catenin ligands will be an important milestone to understand the nature of the protein–ligand interactions at play. Ultimately, we anticipate that this study will benefit the Wnt community by clarifying the binding and mode of action of these compounds. We believe that this is a critical and necessary step toward future structural studies and binding mode elucidation, and ultimately developing suitable strategies for their synthetic optimization toward potent, first-in-class therapeutic agents targeting the Wnt signaling pathway. An analogous study of small-molecule Keap1-Nrf2 PPI inhibitors was undertaken in 2019 by Tran et al., using biophysical techniques [fluorescence polarization (FP), DSF, and surface plasmon resonance (SPR)], which highlighted that half of the reported inhibitors were in fact inactive or deviated from reported activities.^51^

Through an extensive survey of the literature, we here highlight that an important number of reported “direct” β-catenin inhibitors originate from functional and often whole cell screening assays, with limited evidence of target engagement through biophysical binding assessment and structural methods. Critically, we found that none of such inhibitors show in vitro binding to the recombinant β-catenin ARD (aa 148–662) by ITC and DSF. We also highlight that several of these molecules are chemically labile due to pan assay interference (PAIN) chemical functionalities in their core structures, including reactive quinones and α,β-unsaturated carbonyls, redox active systems, reactive 2-hydroxybenzylamine and heterocycles, and metal chelators. The promiscuity of these PAIN substructures in both biophysical and cellular assays has been extensively reviewed and raises significant concerns in regard to the proposed mode of action of these compounds.^52,53^ Last but not least, a subset of reported β-catenin inhibitors originate from proprietary screening libraries, and their syntheses have not been described. Here, we report the detailed synthetic routes developed in the process toward these compounds, along with their characterization and associated analytical data.

## Results and Discussion

### Overview of Reported “Direct” β-Catenin Inhibitors

In 2004, Lepourcelet reported a set of small-molecule inhibitors of the β-catenin (aa 134–668)/Tcf4 (aa 8–54) interaction using a high-throughput ELISA-based assay.^54^ Following a primary screen of 7000 natural products and 45,000 compounds from the Novartis collections, natural products **5**–**10** displayed concentration-dependent inhibition of complex formation. Interestingly, the three structurally related natural products CGP049090 (**6**), PKF115-584 (**7**), and PKF222-815 (**8**) could inhibit the β-catenin/Tcf4 interaction in protein extracts from HCT116 colon cancer cells (IC_50_s = 10–30 μM). In particular, **6** and **7** proved effective in inhibiting Wnt-specific reporter genes, colon cancer cell viability, and β-catenin-dependent axis duplication in *Xenopus* embryos. This is in line with later studies from Sukhdeo et al., highlighting the inhibitory effect of **7** in the xenograft models of human multiple myeloma.^55^ The ability of **6** and **7** to inhibit the β-catenin/APC and β-catenin/tcf4 interactions suggests they bind to β-catenin, however, the binding site and affinities of such interactions remain elusive. Planar polyaromatic compounds **7** and **8** also interfered with tcf4-DNA complexation, independent of β-catenin, whereas **9** and **10** selectively blocked the tcf4/β-catenin complex with DNA, without changes in β-catenin levels.

In 2006, Trosset and co-workers performed a computational pocket detection of the human β-catenin/tcf3 complex (PDB—1G3J) using PASS and FLO_QXP tools to identify potential hotspots for structure-guided drug design,^41,56−58^ and screened a library of 17,700 compounds from the Pharmacia and Upjohn collection against the K435 hotspot. Following docking score analysis and visual inspection of docking poses, 3 out of 22 candidates displayed binding and partial competition with Tcf4 (aa 1–53) in subsequent WaterLOGSY nuclear magnetic resonance (NMR) and ITC. PNU-74654 (**11**) was the most potent and displayed a *K*_d_ of 450 nM against the β-catenin ARD (aa 134–671) in VP-ITC experiments, although no structure–activity relationship (SAR) data were disclosed. On the basis of docking studies, the methyl group of **11** was proposed to bind to a narrow cleft lined by E571 and K508, while the hydrophobic phenoxybenzene was proposed to interact at the polar K435 hotspot. Unambiguous binding mode determination by X-ray crystallography and biological activity data were not reported. Of note, **11** displayed a highly entropic binding signature (Δ*H* = −8.4 kJ/mol, −*T*Δ*S* = 27.8 kJ/mol), raising concerns about its binding specificity.

*R*-etodolac (**12**) and brominated analogue SDX-308 were shown to inhibit β-catenin/TCF signaling (IC_50_ = 683 ± 76 and 46 ± 4.6 μM, respectively, in HEK293 cells) and induce apoptosis in drug-sensitive and drug-resistant multiple myeloma cell lines.^59^ SDX-308 was shown to decrease nuclear β-catenin levels without affecting total β-catenin levels in MM1S and OPM1 cells, thus inhibiting β-catenin/TCF transcriptional activity. A large number of 2,4-diaminoquinazolines were reported as Wnt/β-catenin inhibitors between 2009 and 2016.

In 2009, Chen and co-workers identified lead 2,4-diaminoquinazoline **14** (IC_50s_ = 0.22 and 0.19 μM in Tcf22C11 and Tcf33.13 cell lines, respectively) as a β-catenin/Tcf-4 PPI inhibitor in a cell-based luciferase reporter screening assay and subsequent SAR studies.^60^ However, **14** and its derivatives did not show in vivo efficacy in HT29 and HCT116 xenografts. In 2010, Dehnhardt and co-workers identified a range of 2,4-diaminoquinazoline analogues with improved in vivo profiles. Administration of representative lead molecule **13** (TOPFlash in Tcf33.13 cell line IC_50_ = 0.60 μM) at 150 mg/kg suppressed xenograft tumor growth in vivo by up to 66% in a β-catenin/RK3E mouse model; however, administration was intraperitoneal (IP) due to poor bioavailability.^61^ In 2012, Mao and colleagues reported a series of similar 2,4-diaminoquinazolines, identified using a HCT116 luciferase reporter screening assay and a growth inhibition assay against HCT116 and SW480 cell lines.^62^ Representative **15** inhibited Wnt signaling in HCT116 (IC_50_ = 2.0 μM) and inhibited growth of HCT116 and SW480 cell lines (IC_50s_ = 0.9 and 1.1 μM, respectively). The in vivo activity of **15** and its analogues was not reported. In 2016, Li and co-workers identified a family of 2,4-diaminoquinazolines as Wnt/β-catenin inhibitors using a TopFlash Wnt reporter assay in HCT116 cells, following screening of an in-house library of 500 quinazoline derivatives. SRI-31230 (**16**) was the most potent, inhibiting TOPFLASH (IC_50_ = 13 μM) and inducing dose-dependent reductions in Axin2/cyclinD1/Survivin protein levels while displaying consistent viability reduction in cancer lines HCT116 (IC_50_ = 6.2 μM), SW480 (IC_50_ = 5.9 μM), and SW620 (IC_50_ = 6.7 μM).^63,64^ Although the structural similarities between these molecules are worth noting, the molecular mechanisms and binding events underlying such activity remain unclear. It has been suggested that these compounds exert their effect via the inhibition of the β-catenin/Tcf4 protein–protein interaction, possibly highlighting the folded β-catenin ARD as a possible molecular target.^65^

In 2010, Lee and co-workers screened ∼1500 diverse heterocyclic compounds using a cascade of assays looking at the inhibition of cell proliferation followed by a cell-based reporter assay measuring β-catenin/Tcf transcriptional activity in A549/Wnt2 cells.^66^ Interestingly, primary screening identified 13 structurally related, tri-substituted quinoxalines which reproducibly reduced A439 cell proliferation, including representative derivative **17** (IC_50_ < 5 μM). This correlated with the inhibition of TOPflash reporter gene activity by 30–90% and decreased levels of nuclear β-catenin. A subsequent study identified GDK-100017 (**18**), which inhibited the proliferation of A549/Wnt2 and SW480 cells in a dose- and time-dependent manner (IC_50_ = 10 μM),^67^ while showing little effect in L132 human embryonic pulmonary epithelial cells. This effect in cancer cells correlated with the downregulation of Wnt target genes cyclin D1 and DKK1, while the expression levels of c-myc and survivin remained unaffected. **18** induced cell cycle arrest in A549 cells in combination with radiation compared with either treatment alone. Interestingly, several quinoxaline derivatives have been independently identified by Zhang as Wnt/β-catenin inhibitors, potentially suggesting a common molecular target (vide infra).^68^

In the same year, Ewan et al. used a primary luciferase-based assay to screen a library of ∼63k low molecular weight compounds.^69^ Hit compounds were subjected to a screening workflow that involved a secondary TOPFlash assay, de-convolution, and hit triage, which afforded three hits. CCT031374 (**19**) was among the three most promising compounds, with GI_50_ = 13.9 and 13.2 μM in HCT116 and SW480 cells, respectively. **19** was hypothesized to act at the β-catenin level, by blocking TCF-dependent transcription and increasing the degradation of endogenous wt-β-catenin in murine L-cells, was stabilized by the GSK3 inhibitor BIO. The molecular mechanism underlying inhibition was not elucidated, although it was hinted at possible binding of **19** to the ARD. The blocking of TCF-dependent transcription in SW480 colon cancer cells was not accompanied by the downregulation of β-catenin levels. Furthermore, the levels of stabilized β-catenin in HEK293 cells were not reduced when treated with **19**, although TCF-dependent transcription was blocked.

In 2011, Gonsalves employed a cellular RNA-based screening assay with a luciferase reporter to identify small-molecule inhibitors of β-catenin/Tcf4-mediated transcription downstream of the DC, following induction of reporter gene activity via RNAi-mediated Axin inhibition.^70^ This screen of ca. 15,000 compounds from various proprietary collections identified a family of structurally related heterocyclic compounds displaying >70% inhibition of signaling. Representative oxazole containing iCRT3 (**20**) was among the most potent analogues, selectively inhibiting nuclear β-catenin-mediated transcription (IC_50_ = 1.8 μM), β-catenin/Tcf4 complex formation in vitro and in HEK293 cell lysate. Conversely, **20** had no noticeable effect on β-catenin interactions with *E*-cadherin and α-catenin, or noncanonical Wnt signaling. **20** was hypothesized to bind the K435/R469 hotspot of the ARD based on docking and DSF experiments, although the exact molecular interactions involved and the associated affinity of such an interaction have not been discussed. Of note, **20** induced thermal shifts <1 °C in DSF experiments, suggesting that potential binding affinity to the ARD is likely to be rather weak (e.g., high μM), contrasting with its reported cellular activity (low μM).^68^ Of note, some analogues including iCRT-4, -9, -16, and -21 significantly interfered with several signaling pathways including notch, *Hedgehog* (*hh*), and Janus kinase/signal transducer and activator of transcription.

The same K435_βcat_-D16_tcf4_ interaction hotspot on the ARD was probed in 2012 by Tian and co-workers, by Autodock4 virtual screening of ca. 2000 compounds from a diversity set of the National Cancer Institute open database.^71,72^ Docking identified organocopper complex BC21 (**21**) as a potential ligand of β-catenin. Complex **21** decreased β-catenin-dependent TCF reporter activity in HCT116 and HEK293 cells at low micromolar concentrations and modestly downregulated Wnt target genes (c-Myc and cyclin D1) and reduced HCT116 viability (IC_50_ = 15 μM); while in FP competition experiments, **21** displayed modest displacement of a labeled tcf4 peptide (IC_50_ = 5 μM), the precise molecular mechanisms and binding specificity are unknown. Also noted by the authors, **21** had also been shown to inhibit protein phosphatase 2C, the proteasome, and to have cytotoxic effects in a range of human cancer cells.^73,74^ These potential off-target effects were not investigated in this study.

In the same year, Henen and co-workers employed a meta-structure approach combined with NMR to pinpoint potential β-catenin ligands.^75^ The meta-structure concept was proposed as a framework to identify potential ligands directly from a protein’s primary sequence. This identified fluorescein (**22**), eosin Y (**23**), 2-phenoxybenzoate (**24**), and related imine derivative (**25**) as potential ligands of the ARD. Both ligands showed binding to the β-catenin ARD (aa 134–671) in saturation transfer difference (STD) NMR experiments. However, the affinity and precise binding mode/site of these ligands were not reported. It is also worth noting that both aromatics of the biphenylether in **24** and **25** are unlikely to adopt a coplanar arrangement but rather give rise to a significant dihedral angle. This suggests that all four compounds may not share a unique binding mode/site. Although the authors highlighted their plan to exploit these results for the development of more potent β-catenin ligands, no follow-up study has been reported.

An ELISA-based screen of 47,500 LOPAC/PhytoPure/MRCT compounds by de la Roche identified the natural product carnosic acid (**26**) as a β-catenin/Bcl9 PPI inhibitor.^76^**26** selectively inhibited (*K*_i_ = 3 μM) the interaction between the N-terminal region of β-catenin (aa 134–671)^77^ and the BCL9 homology domain 2 (aa 343–396). **26** is thought to bind (STD/HSQC-NMR, *K*_d_ ∼ 20 μM) to a transient binding motif in part formed by the intrinsically labile helix 1 (H1, aa 141–149) of the ARD. The interaction of **26** with this region was shown to exacerbate the propensity of the ARD to aggregate in vitro and in cells, leading to its proteasome-mediated depletion. This proposed degradative mode of action was also correlated with the downregulation of β-catenin-dependent TOPFlash activity and the downregulation of the mRNA levels of Wnt targets AXIN2 and B9L in HeLa, HCT116, and SW480 cells. The peculiar degradative mode of action of **26** has so far hampered accurate biophysical and structural characterization, which rely on the presence of the folded protein.

In 2013, Zhang et al. using in-house AlphaScreen and FP assays to look at selective inhibitors of the β-catenin (aa 138–686)–Tcf4 (aa 7–51) interaction, screened a library of 250 commercially available carboxylic acid-containing compounds.^68^ Substituted acylhydrazide R360163 (**27**), rhodanine L338192 (**28**), quinoxaline R999636 (**29**), and biphenylether T155535 (**30**) were identified as modestly selective inhibitors showing moderate potency (*K*_i_ ∼ 8.8–77 μM) and selectivity (1.1–25 fold) in vitro for the β-catenin-Tcf4 interaction over APC and *E*-cadherin. These compounds have been proposed to engage the β-catenin K435 hotspot targeted by D16 of Tcf4, although direct evidence through structural methods or site-directed mutagenesis (SDM) has not been reported. Interestingly, **30** is yet another example of a biphenylether derivative. The Ji laboratory has used a structure-based design strategy toward several series of small-molecule peptidomimetics targeting the ARD. Isosteric replacement and conformational restriction of D16/E17 of Tcf4 led to the development of UU-T01 (**31**), the most potent member of a family of heterocyclic β-catenin/Tcf4 interaction in vitro inhibitors.^78^ The binding of **31** to the ARD (aa 142–686) was characterized by FP (*K*_i_ = 3 μM) and VP-ITC (*K*_d_ = 0.53 μM, Δ*H* = −3.4 kJ/mol, −*T*Δ*S* = −33 kJ/mol). Of note, this is an unusually high ligand efficiency (LE = 0.51), in addition to the binding being strongly driven by a favorable entropic term. Based on modeling studies, **31** was proposed to mimic D16/E17 of Tcf4 and engage in polar interactions with β-catenin hotspot residues K435, K508, and R469. This was further supported by its reduced binding affinity against the corresponding K435A, K508A, and R469A mutants. However, the authors did not report on the biological activity of **31**. A third publication from the Ji laboratory the following year reported disruptors of the β-catenin-Tcf4 PPI, utilizing a peptidomimetic strategy in conjunction with computational modeling.^79^ SiteMap and MCSS analyses assessed the druggability of the region of β-catenin that interacts with the GANDE sequence in Tcf4.^48,80^ Using the Tcf D16/E17 hotspot residues as a basic scaffold, diverse motifs were incorporated to probe and evolve the peptidomimetic. MCSS analysis suggested that a chloroindole motif would be tolerated in a narrow channel formed by residues R474 and R515 at the ARD surface, adjacent to the K435 hotspot. This channel is present in the crystal structures of the ARD bound to human TCF4 (PDB—1JDH, 1JDW) and xenopus Tcf3 (PDB—1G3J) and accommodates these regulators, but is not present in the ARD apo structure (PDB—2Z6H). Furthermore, a range of hydrophobic amino acids were examined as replacements for N15 to probe the adjacent hydrophobic pocket (Figure 3B) formed by residues K508, E568, and I569, among others. Iterative computational modeling (AutoDock 4.2) and SAR from FP experiments afforded **39** (*K*_i_ = 0.64 μM). AlphaScreen, VP-ITC, and SDM all supported the direct binding of **39** and derivatives to β-catenin (VP-ITC *K*_d_ ≈ 0.42 μM). The protein expression levels of cyclin D1 and c-Myc were reduced upon treatment with **39** in SW480 cells. Equally noteworthy, the binding of **34** is highly entropic, suggesting that it is primarily entropically driven (Δ*H* = −4.2 kJ/mol, −*T*Δ*S* = −33 kJ/mol). This suggests again that binding may be driven primarily by hydrophobic interactions and water displacement rather than polar and directional interactions (e.g., H-bond). This was not discussed by the authors.

The Ji laboratory reported the small molecule **32** in 2015 as an inhibitor of the β-catenin-Tcf4 interaction identified through dual AlphaScreen and FP assays and SAR analysis.^81^ One close derivative of **32** suppressed canonical Wnt signaling, downregulated the expression of Wnt target genes, and inhibited the growth of cancer cells.

Hoggard et al. used HippDB to data mine the PDB and identify α-helical peptidomimetics to target the β-catenin/BCL9 PPI.^82−86^**34** was designed by a bioisostere-based fragment hopping protocol to mimic hydrophobic side chains at positions *i*, *i* + 3 and *i* + 7 of an α-helix. **34** was subsequently overlayed with the BCL9 S352-F374 α-helix to design small-molecule inhibitors of the β-catenin-BCL9 PPI. Iterative design-synthesis-evaluation were performed to identify **34** as the most potent and selective inhibitor with *K*_iαS_ = 2.1 μM. **34** was further characterized by SDM and VP-ITC (*K*_d_ = 0.33 μM). Later in 2018, the derivatives of **34** were reported with *K*_iαS_ = 0.47 μM and selectivity >1900 (β-catenin-BCL9 vs β-catenin-E-cadherin, respectively).^87^ Along with carnosic acid **26**, **34** and its derivatives represent the only examples of small molecules targeting the ARD N-terminus. The inhibition of the β-catenin/BCL9 PPI has been proposed as an alternative strategy to achieve controlled Wnt signaling inhibition in a range of CRCs.^88^ The interaction of β-catenin with BCL9 in the nucleus is necessary for downstream signaling. BCL9 is one of the few partners interacting with the ARD N-terminus, suggesting that the development of selective PPI inhibitors may be possible. This may have important implications for overcoming the challenge of achieving selective inhibition of the β-catenin/Tcf4 oncogenic PPI. This is because Tcf4 shares overlapping PPI surfaces with a range of other regulators, notably *E*-cadherin. Wnt signaling activation and alteration of *E*-cadherin function are key factors in the epithelial–mesenchymal transition and metastasis.

Early in 2016, Fang and Birchmeier published 4-thioureido-benzenesulfonamide derivative LF3 (**35**) as an inhibitor of the β-catenin (aa 134–668) and Tcf4 (aa 1–79) PPI in an AlphaScreen high-throughput screening (HTS) of 16000 synthetic compounds from the ChemBioNet collection.^89^ Thiourea **35** exhibited an IC_50_ ca. 2 μM in AlphaScreen and ELISA assays, although the affinity and binding site/mode of **35** at the ARD surface were not reported. Preliminary SAR studies highlighted that the sulfonamide group is vital for potency, illustrated by a complete loss of potency upon its removal. The replacement of the styryl unit with other terminal aromatic groups such as phenyl and benzyl led to a 2–50 fold reduction in inhibitory activity. Immunoprecipitation experiments showed that **35** could inhibit β-catenin/Tcf4 interaction in HCT116 cell lysates in the micromolar range while showing no disruption of *E*-cadherin-mediated cell adhesion. This also correlated with the downregulated expression of a range of Wnt target genes (*Bmp4*, *Axin2*, *survivin*, *Bambi*, and *c-Myc*) and tumor growth reduction in NOD/SCID mice.

In the same year, Zhang and co-workers reported the small molecule BC-23 (**33**).^90^ The treatment of non-small cell lung cancer H1299 cells with low μM concentrations of **33** downregulated c-Myc and cyclin D1 expression, promoted the S phase arrest and increased levels of reactive oxygen species (ROS) to mediate increased radiation sensitivity. In FP experiments, **33** inhibited the β-catenin/Tcf4 (aa 8–30) PPI (IC_50_ = 1.7 μM) and inhibited TOPFlash activity in a dose-dependent manner (IC_50_ = 2.3 μM) in H1299 cells. Based on Autodock4 docking studies, the authors suggested that **33** competes with tcf4 through binding to the K435 hotspot, making hydrophobic contacts and H-bond interactions with the side chains of neighboring residues C429, N430, and K508. A key consideration is the chemical lability of the chloroquinone motif, which is highly reactive toward a range of nucleophiles, notably sulfur nucleophiles such as cysteine at physiological pH. We will discuss this later in this study. This raises important concerns regarding the specificity and mode of action of BC-23.

Hwang and Lee reported the small molecule benzenesulfonamide derivative methyl 3-{[(4-methylphenyl)sulfonyl]amino}benzoate (MSAB) (**36**) following a cell-based HTS luciferase assay of 22,000 compounds.^91^**36** selectively inhibited luciferase activity (IC_50_ ∼ 2–5 μM) and reduced viability (IC_50_ ∼ 2–6 μM) of 12 Wnt-dependent cancer cell lines, while showing comparatively little effect on Wnt-independent cell lines. **36** selectively induced size and weight reduction in a panel of Wnt driven HCT116, HCT115, and H23 tumors in xenografted mouse models. This was in line with apoptotic induction and elevated cleaved caspase-3 in mouse tumor tissues treated with **36**. This effect also correlated with dose-dependent reduction of mRNA levels of downstream Wnt target genes AXIN-2, c-MYC, cyclin D1, and BMP4 in a panel of Wnt-dependent cell lines, along with reduced levels of their respective proteins. Initial biophysical analysis using STD NMR, SPR, and pull-down experiments suggested that **36** binds to the ARD C-terminal region (aa 301–670). The authors suggested that the binding of **36** to β-catenin in the cellular environment mediates its proteasomal degradation, although this remains largely speculative.

Substituted benzimidazole HI-B1 (**37**) was reported in 2017 by Dong et al.^92^**37** inhibited β-catenin/Tcf4 luciferase activity in a dose-dependent manner (IC_50_ = 2.3 μM), downregulated the expression of cyclin D1 and Axin2, and induced growth reduction of DLD1, CACO2, and HCT116 cells. **37** also selectively inhibited the growth of patient-derived xenograft CRC model displaying elevated β-catenin levels. **37** conjugation to sepharose 4B-beads followed by affinity pull-down in cell lysates and western blotting (WB) selectively identified β-catenin as a direct molecular target (affinity unknown). Of note, **37** presents several structural similarities with GDK100017 (**18**), including a fluorinated nitrogen heterobicyclic system connected to a conformationally constrained phenyl ring. Glide docking suggested that **37** binds to the surface of the ARD, possibly through key H-bonding interactions with G307 and K312.

Several series of β-carbolines were independently reported as Wnt signaling inhibitors between 2014 and 2018. Wang and co-workers identified compound SP141 (**38**) as a dual inhibitor of MDM2 and β-catenin in pancreatic cancer.^93^**38** potently reduced the levels of active, unphosphorylated β-catenin and downstream c-Myc and cyclin D1 proteins in a concentration-dependent manner in Panc-1 and AsPC-1 pancreatic cancer cells (IC_50_s < 0.5 μM) and tumors (40 mg/kg), harboring elevated levels of β-catenin. Interestingly, WB and immunofluorescence experiments showed that **38** had comparatively low effects on the levels of phosphorylated β-catenin and the membrane bound β-catenin fraction, suggesting that it selectively targets the soluble, active pool of cytoplasmic and nuclear β-catenin. Pull-down experiments in Panc-1 and AsPC-1 cells with a biotinylated analogue of **38** preferentially precipitated β-catenin, suggesting it as a direct target (affinity unknown). The overall molecular mechanisms underlying such inhibition remain unclear, although it was suggested that binding of **38** to a yet to characterize region of the ARD could induce its aggregation and proteasome-mediated degradation. A similar degradative mode of action was suggested by de la Roche while studying carnosic acid (**26**).^76^ It is also worth mentioning that the structurally related beta-carboline derivatives have been reported to inhibit Wnt signaling through the activation of CK-1α rather than binding to β-catenin.^94^ Further clarification through direct binding assays will be critical to validate the molecular target(s) of beta-carbolines.

### PAINS Analysis and Biophysical Characterization

Pan Assay INterference compoundS (PAINS) contain substructural motifs which are notorious for their overrepresentation as hits across a diverse range of HTS assays.^52^ PAINS are well-known to medicinal chemists and often display unspecific chemical reactivity and adverse physico-chemical properties, leading to promiscuous, nonspecific activity in various in vitro biophysical, biochemical, and cellular assays. Those include a wide range of chemically reactive electrophiles, metal chelators, redox active or/and aggregation prone scaffolds, and extended aromatic systems displaying DNA intercalating or/and intrinsic photochromic properties that interfere with, for example, absorption/fluorescence assay readouts, and molecular scaffolds historically well-known for their intrinsic in vivo toxicity. Some PAINS can also cumulate several of such properties. To the despair of many medicinal chemists, they represent a significant proportion of false positive hits in screening campaigns, either lacking potency or specificity. PAINS have been extensively reviewed and reported since their popularization in the medicinal chemistry literature more than a decade ago, notably thanks to important ground work by Baell and co-workers.^53,95−97^ The increasing recognition of PAINS in screening libraries has promoted considerable development of computational filters for their early detection and removal, hence minimizing the time and resources invested in pursuing non-optimizable leads.^98^ There has been some exceptions and rarely PAINS can be optimized to promising candidates, such as the hit anthraquinone PARG inhibitor identified by AstraZeneca, which was successfully developed to a lead quinazolinedione with *K*_d_ = 1.45 nM.^99^ Brenk filters have also proven useful for the early detection and removal of promiscuous functionalities generally known to display unfavorable toxicity, poor pharmacokinetic properties, chemical reactivity, and metabolic instability.^100,101^

Here, we sought to investigate the occurrence of PAINS and/or Brenk alerts in reported small-molecule β-catenin inhibitors, which we believe is a critical first step to (i) discarding potentially inherently toxic/reactive “dead-end” compounds; and (ii) identifying potentially suitable starting points for future structural studies and lead optimization toward first-in-class chemical probes and therapeutic agents targeting β-catenin. First, we combined visual inspection and extensive PAINS filters,^53^ complemented with SwissADME computational analysis, to investigate potential inherent toxicity/reactivity alerts (Table 1). SwissADME has proven particularly useful and robust for the identification of problematic substructures, and combines the dual detection of PAINS and Brenk alerts into a single platform.^101^ Second, we assayed the majority of these compounds using biophysical techniques. We employed DSF and ITC to evidence potential binding through monitoring thermal stabilization and binding enthalpy, respectively. These methods are complementary as ITC focuses on detecting rather enthalpic binding events while DSF tends to underscore binding events carrying more important entropic contributions, especially for high melting temperatures (*T*_m_, vide infra).

**Table 1:** Literature and SwissADME PAINS/Brenk Analysis Reveal That Approximately Half of Reported β-catenin Inhibitors Contain Suspected Reactive and/or Toxic Substructures^a^

		SwissADME analysis			
ID	literature reactivity	PAINS	Brenk	alert	detergent additives in reported in vitro screen^d^
**5**, **9**	Rx, Pr Re	Y	Y^b^	quinone_A/D	0.05% Tween 20
**6–8**	Rx, Pr, D Re	N	Y	polycyclic_aromatic	0.05% Tween 20
**22–23**	Rx, Pr, D Re	N	Y	polycyclic_aromatic	none
**10**	Rx Cycler	N	N	toxoflavin^c^	0.05% Tween 20
**11**	PI, H, M	N	Y	Imine_1	none
**13**	E	N	Y	2-halo_pyridine	N/A, whole cell
**21**	Re, M, Cx	N	N	mannich_A	0.01% Triton X-100
**25**	PI, H	N	Y	Imine_1	none
**26**	Rx, Pr Re	Y	Y	catechol_A	0.05% Tween 20
**27**	E	Y	Y	imine_one_isatin	0.01% Triton X-100
**28**	E, M, Ag	Y	Y	ene_rhod_A	0.01% Triton X-100
**32**	E	N	N	diazox_A	0.01% Triton X-100
**33**	E, Rx, Pr Re	Y	N	quinone_A	0.01% Triton X-100
**35**	E	N	Y	thiocarbonyl_group	0.05% Tween 20

a Notes: Pr = protein, D = DNA, M = metal chelation, PI = photoisomerization, E = electrophilic, H = hydrolysis, Rx = redox, Re = reactive, Cx = cytotoxic, I = intercalation, and Ag = aggregation.

b **16** did not trigger Brenk alert.

c No alert was generated.

d Detergent additives in reported in vitro screens: identified from whole cell assays, **12**–**20**, **37**, **38**, no detergent used; identified from in vitro screens, **5**–**10**, **26**, **35**, in the presence of 0.05% Tween 20; **21**, **27**–**34**, **36**, **39**, in the presence of 0.01% Triton X-100; and **11**, **22**–**25**, no detergent in the screening assay.

### Quinone/Haloquinone and Extended Polyaromatics

**5**–**9**, **22**, **23**, and **33** all contain quinone, quinone methide, or polyaromatic motifs prone to redox activity, electrophilic reactivity, and singlet oxygen production.^102^ ALARM NMR experimentally demonstrated that **10** and pyrimidotriazinedione derivatives were intrinsically reactive along with displaying potent redox cycling in a phenol red-horseradish peroxidase screen (EC_50_ < 2.0 μM).^103−105^ The same two assays also unambiguously demonstrated the inherent thiol reactivity and redox cycling of a diverse range of substituted rhodanines, chloro- and/or benzo-quinones, catechols, benzofurazans, and other extended polyaromatic systems, reminiscent of the chemical structures of **5**–**9**, **26**, **28**, **32**, and **33**. The core scaffolds of **5**–**9** also share structural similarities with a number of anthracyclines, which are well-known for their DNA-damaging activity.

### Benzofurazans

The benzofurazan PAIN scaffold is known to react reversibly with thiols, including by electron transfer.^95^ Benzofurazans are widely employed as fluorescent-labeling reagents due to their high reactivity and long excitation/emission wavelengths.^106,107^ A number of electrophilic benzofurazans have also displayed modest inhibition of DNA and RNA synthesis.^108^ Among other electrophilic substructures (vide supra), an important number of benzofurazans exhibit intrinsic reactivity toward nucleophiles,^104^ raising questions about the specificity of electrophilic pyrazinofurazan **32**.

### Phenolic-Mannich Base

**21** contains a phenolic Mannich base motif prone to in situ benzylic ammonium elimination (p*K*_a_ ≈ 10) to the corresponding highly electrophilic *ortho*-quinone methide (QM).^109^ QMs have generally been shown to react indiscriminately with a number of biological nucleophiles, including amino acid side chains and DNA bases. Two publications have also highlighted **21** as an inhibitor of both PP2C and flavivirus MTase, suggesting other possible modes of action.^74,110^**21** also exhibited cytotoxicity in BHK-21 cells (CC_50_ = 11 μM), at concentrations lower than its IC_50_ = 17 μM.^110^ A range of phenolic Mannich base-containing compounds structurally similar to **21** have also been shown to covalently modify catalytic tautomerase via QM generation and subsequent proline N-alkylation.^111^ These stability issues, along with the potential for in situ metal decomplexation in the physiological environment, suggest that **21** may act by more than one mechanism.

### Acylhydrazone

Acylhydrazones similar to commercial **11** and **27** are known to undergo photoinduced *E*/*Z* isomerization^112^ and hydrolysis to their corresponding hydrazides and aldehydes. Acylhydrazones are generally less stable to hydrolysis than non-acylated hydrazones and oximes.^113^ Because of their facile hydrolysis under mildly acidic conditions, acylhydrazones have been employed as cleavable linkers for biotin-streptavidin biomolecule isolation.^114^

### Rhodanines

Despite the range of biological activities reported in the literature, rhodanines are regarded as one of the most prevalent sources of false positives in HTS campaigns. Prior to 2010, the rhodanine motif was prevalent in multiple drug discovery campaigns and reached a peak in 2010.^115−119^ However, since the introduction of systematic PAINS filters by Baell in 2010, there has been a significant decrease in reports highlighting rhodanine lead compounds.^53^ Rhodanines such as **28** have been shown to form aggregates, act as Michael acceptors, interfere with fluorescence assays due to their photochromic properties, and chelate a variety of metals. These have been extensively reviewed by Tomašić^119^ and Sink.^120^ Consistently, rhodanine containing **28** triggered both PAINS and Brenk alerts.

### Catechols

Catechols such as the natural product carnosic acid **26** are frequent hitters in screening assays such as AlphaScreen.^96^ They are notorious redox cyclers,^121^ metal chelators,^122^ and potent electrophiles in their oxidized form.^123^ For example, catechol containing gallic acid has been shown to induce apoptosis in HL-60RG cells by ROS generation.^124^**26** has been extensively investigated for its anti-bacterial, -cancer, -fungal, and -viral properties,^125^ and multiple reports have also demonstrated that **26** is easily oxidized in mild aerobic conditions. Although de la Roche provided supporting evidence for its binding to the ARD in vitro, it is still unclear whether **26** itself or one of its oxidized metabolites is responsible for its cytotoxicity.

The majority of the abovementioned known promiscuous scaffolds were identified as problematic by either PAIN or Brenk filtering, or both. Exceptions were toxoflavin (**10**), labile copper complex (**21**), and polycyclic furazan (**34**), which were not identified by the filters, despite their known chemical lability (vide supra). We found that the Brenk alert filter of SwissADME was generally more efficient at detecting potentially reactive and/or toxic substructures than more traditional/historical PAIN filters, and generated results more in line with the known promiscuity of these scaffolds. Last but not least, an important proportion of these molecules are based on chemotypes well known to aggregate and form colloids in aqueous buffers, interfering with a variety of screening assays. This was recently demonstrated in several excellent studies, highlighting compound aggregation as a major source of false positives in in vitro screening assays.^126,127^ Although the use of detergent additives can to some extent mitigate this behavior, the presence of aggregation prone chemotypes present in reported β-catenin ligands is another important consideration (Table 1).

Regardless of the presence of PAIN/Brenk motifs, we examined the binding of these compounds to the ARD (aa 148–662). A small number of compounds were available commercially (see Supporting Information Section), while a large proportion originated from proprietary compound libraries. We re-synthesized these in-house and here provide detailed synthetic routes and associated analytical data (Supporting Information, Schemes S1–S18). We postulated that any candidate displaying cellular activity at a low micromolar concentration is likely to exhibit a binding affinity in a similar concentration range or below, and should elicit a sharp response in our assay conditions (Experimental Section, Supporting Information). Positive control TCF4 (aa 13–27) and BCL9 (aa 348–376) peptides displayed *K*_d_s = 1.81 ± 0.11 and 1.25 ± 0.06 μM, respectively, in line with previous reports and demonstrating that our ARD construct is folded and functional (Table 2, Figure S5).^42,45^ However, to our disappointment, but in line with the abovementioned PAIN/Brenk analysis, none of these compounds did show a measurable binding enthalpy in ITC, and failed to induce significant thermal stabilization in DSF experiments (Tables 2 and S2–S9, Figure S4). For example, representative CGP049090 (**6**) did not show any binding enthalpy to the ARD in ITC, nor did fluorescein (**22**), eosin Y (**23**), or BC-23 (**33**). Also, in contrast with VP-ITC binding data reported by Trosset and suggesting submicromolar binding affinity to the ARD, in our hands, purified PNU-74654 **11** (both commercial and re-synthesized) surprisingly and consistently did not exhibit noticeable binding enthalpy in repeat ITC200 experiments, and consistently showed an absence of protein stabilization in DSF experiments. Interestingly and consistent with previous reports,^21^ MSAB (**36**) showed a positive STD response in our hands despite showing little enthalpy and protein stabilization. We also wondered whether β-catenin inhibition by **36** in cells may be mediated by the in situ hydrolysis of the methyl ester to generate a potentially more active carboxylic acid analogue. To test this hypothesis, we synthesized carboxylic acid analogue **96** by alkaline hydrolysis of **36** (Scheme S16). Although **96** was soluble in aqueous media at a higher concentration (>1 mM) compared to parent MSAB (**36**), it also failed to exhibit any binding affinity or thermal stabilization of the ARD, discarding in situ activation as a potential cause for engaging the ARD in cells. Based on these results, we propose that **36** is hydrophobic and that the observed STD signal is a result of weak and unspecific binding to hydrophobic patches of the ARD. Such effects have been extensively documented and recently discussed by Zega in an excellent review.^128^ Overall, none of the compounds scrutinized in this study showed consistent binding across the biophysical cascade we employed.

**Table 2:** Thermal Stabilization (Δ*T*_m_), Binding Affinities (*K*_d_), and Thermodynamic Parameters (Δ*G*, Δ*H*, and Δ*S*) Determined by DSF and ITC (298 K), Respectively, against the WT Human ARD (aa 148–662)^a^

compound ID	DSF Δ*T*_m_ (°C)	ITC binding (Y/N)	*N*	*K*_d_^e^ (μM)	Δ*G* (kcal/mol)	Δ*H* (kcal/mol)	–*T*Δ*S* (kcal/mol)
**TCF4**_**13–27**_	+0.1 ± 0.1	Y	0.94 ± 0.01	1.81 ± 0.11	–7.84	–18.7 ± 0.23	10.9
**BCL9**_**348–376**_	–0.5 ± 0.1	Y	1.09 ± 0.01	1.25 ± 0.06	–8.05	–11.3 ± 0.09	3.18
**6**	–0.7 ± 0.3	N		n.d.			
**10**	–0.5 ± 0.1	N		n.d.			
**11**	–0.5 ± 0.1	N		n.d.			
**12**	–0.5 ± 0.1	N		n.d.			
**15**	–0.7 ± 0.4	N		n.d.			
**16**	–0.6 ± 0.0	N^b^		n.d.			
**17**	0.0 ± 0.0	N		n.d.			
**18**	+0.1 ± 0.1	N		n.d.			
**19**	–0.1 ± 0.3	N		n.d.			
**20**	–0.7 ± 0.3	N		n.d.			
**21**	n.d.^b^	N^b^		n.d.			
**22**	+0.1 ± 0.1	N		n.d.			
**23**	n.d.^c^	N		n.d.			
**24**	–0.1 ± 0.3	N		n.d.			
**25**^d^	+0.1 ± 0.1	N^b^		n.d.			
**26**	–2.7 ± 0.7	N		n.d.			
**28**	–0.5 ± 0.1	N		n.d.			
**29**	–3.9 ± 0.4	N		n.d.			
**30**	+0.1 ± 0.1	N		n.d.			
**32**	–0.1 ± 0.3	N		n.d.			
**33**	n.d.^c^	N		n.d.			
**34**	–0.5 ± 0.1	N		n.d.			
**35**	–0.7 ± 0.3	N		n.d.			
**36**	–0.5 ± 0.1	N		n.d.			
**37**	–0.1 ± 0.3	N		n.d.			
**38**	–0.9 ± 0.3	N		n.d.			
**96**	–0.5 ± 0.1	N		n.d.			

a DSF experiments were performed in triplicate, using 8 μM ARD, 10× SYPRO Orange, and 125 μM compound, in 25 mM Tris (pH 7.4, 200 mM NaCl, 0.06% NaN_3_, 1 mM DTT, 5% v/v DMSO). ITC experiments were performed at 25 °C, using the same buffered conditions. Purified TCF4_13–27_ and BCL9_348–376_ peptides were used as positive controls.

b Could not be evaluated due to poor solubility.

c Interference from compounds inherent fluorescence.

d Partly hydrolyses in aqueous media.

e n.d.: no binding detected at the highest concentrations of the protein and ligands tested (Tables S2–S9).

## Conclusions

In this study, we provide a comprehensive overview of claimed (and some putative) “direct” small-molecule inhibitors of β-catenin reported in the literature, along with their binding properties and proposed molecular mode of action. However, we show that the majority of these molecules contain PAIN/Brenk structural motifs, notorious for their promiscuity in screening assays. Regardless, we assayed these compounds in orthogonal biophysical assays, looking at both binding affinity (ITC) and protein thermal stabilization (DSF). In line with their suspected promiscuity, we demonstrate that none of these molecules exhibit a direct measurable interaction with purified human ARD, at least not in the concentration range reported for their bioactivity. ITC, in particular, is a label-free technique, and while considered one of the gold standards to assess biomolecular interactions, it has not been systematically employed in the field. We also highlight that despite important progress in the development of PAIN filters in the last decade, a number of known problematic substructures are still escaping triage, potentially leading to the investigation of flat SAR and/or “dead-end” scaffolds. We believe that this has been an important contributor to the common view that β-catenin is an undruggable target and perhaps partly explains why none of the molecules presented above made it to advanced clinical evaluation. Altogether, our data evidence that apart from a small number of high molecular weight peptidomimetics, there is currently no unambiguously confirmed, potent small-molecule ligand of the β-catenin ARD. Although it is not impossible that some of these compounds identified in phenotypic screens may bind to β-catenin N- or C-terminal regions, it appears unlikely as there is sufficient evidence that they are unstructured and deprived of a well-defined, druggable pocket. One possibility is that some of these compounds target transient binding sites formed upon association of the N- or C- termini with co-regulators such as TCF4 and BCL9. Simonetta and co-workers^129^ recently reported a series of trifluoromethylated pyridone derivatives acting as “molecular glues” that stabilize the interaction of the β-catenin N-terminus (aa 16–50) with SCF^β-TrCP^ E3 ligase via binding to a narrow transient pocket at the PPI interface in vitro. However, it is not completely clear whether this pocket is present upon complex formation with FL β-catenin in cells. Recently, a number of anthraquinone derivatives, such as BC-2059 (Tegavivint), were shown to inhibit Wnt signaling through binding to transducin beta-like protein 1 (TBL1) and inhibiting its interaction with nuclear β-catenin, making the latter vulnerable to degradation.^130^ In the future, it would be interesting to see whether the compounds discussed above have any effect on the TBL1:β-catenin interaction. We also cannot discard that the binding of some of these compounds in cells may depend on specific, although yet to characterize post-translational modifications at the surface of β-catenin. However, the complete absence of binding to recombinant ARD makes it improbable, and more likely, they act through another, yet to be identified, molecular mechanism in cells. Here, we aim to draw attention and caution to the proposed cellular mode of action of these inhibitors, and suggest they may exert cytotoxicity and inhibit signaling via other, possibly β-catenin-independent mechanisms. These avenues will be the focus of future studies in our laboratories. These results are significant as many of these molecules are still sold as β-catenin ligands at a high price, and this study to a large extent debunks the gaps in knowledge regarding their binding properties and will inform the community on their suitability as chemical probes of Wnt signaling. This work will also likely have important implications in the (re)interpretation of a large number of published studies employing these molecules as β-catenin inhibitors. We believe that an important focus of future screening campaigns should be the choice of suitable assay cascades with lower susceptibility to false positives for identifying new direct β-catenin inhibitors. Critical to success will be the combination of orthogonal in vitro assays for hit enrichment and validation, to inform structural studies and future lead optimization. For example, representative methods such as DSF, NMR, and ITC seem well-positioned for that purpose and span a wide throughput versus accuracy range, and their combination has proven effective in the fragment-based ligand discovery area for targeting highly challenging PPIs previously thought undruggable.^131^ While preparing this manuscript, colleagues from Boehringer Ingelheim used a combination of NMR and microscale thermophoresis to cross-validate fragment-sized ligands of the ARD, and reported the first crystal structure of a fragment-sized molecule bound to a truncated ARD construct (repeats 1–4).^132^ Although these fragments display modest affinity (*K*_d_ = 915 μM, LE = 0.20) and do not overlap with relevant PPIs at the ARD surface, this provides support for such in vitro screening cascades. Overall, we believe this study provides compelling evidence that β-catenin remains a highly attractive target in cancer.

## Supporting Information Available

Synthetic procedures and compound characterization data, protocols for recombinant protein production and peptide synthesis, protocols for biophysical studies, ITC titrations of small molecules and peptides, representative NMR spectra and high-performance liquid chromatography traces, and selected DSF-generated protein melting curves (PDF)
Molecular formula strings (CSV)

## Abbreviations

Δ*G*: Gibbs free energy
Δ*H*: enthalpy change
Δ*S*: entropy change
APC: adenomatous polyposis coli
ARD: armadillo repeat domain
BCL9: B-cell CLL/lymphoma 9 protein
CBP: cyclic adenosine monophosphate response element binding protein binding protein
CK1α: casein kinase 1α
CRC: colorectal cancer
CTD: C-terminal domain
Da: Dalton
DC: destruction complex
DSF: differential scanning fluorimetry
EMT: epithelial–mesenchymal transition
FBLD: fragment-based ligand discovery
FP: fluorescence polarization
GI_50_: compound concentration for 50% of maximal inhibition of cell proliferation
GSK3β: glycogen synthase kinase 3 beta
HD2: homology domain 2
HRP: horseradish peroxidase
ICAT: inhibitor of β-catenin and TCF
IP: immunoprecipitation
ITC: isothermal titration calorimetry
*K*_a_: association constant
*K*_d_: dissociation constant
KEAP1: Kelch-like ECH-associated protein 1
LE: ligand efficiency
LEF: lymphoid enhancer-binding factor
LOGSY: ligand observed via gradient spectroscopy
LRP: low density lipoprotein receptor-related protein
MDM2: mouse double minute 2 homolog
MMP-7: matrix metallopeptidase 7
MSAB: methyl 3-{[(4-methylphenyl)sulfonyl]amino}benzoate
MST: microscale thermophoresis
n: stoichiometry
NRF2: nuclear factor erythroid 2-related factor 2
NTD: N-terminal domain
NSCLC: non-small cell lung cancer
PAINS: pan assay interference compounds
PPI: protein–protein interaction
RYK: related to receptor tyrosine kinase
SDM: site-directed mutagenesis
STAT: signal transducer and activator of transcription
STD: saturation transfer difference
TCF4: transcription factor 4
*T*_m_: protein melting temperature
WB: western blotting
Wnt: Wingless-related integration site
