# Supplementary material for: Biophysical Survey of Small-Molecule β-Catenin Inhibitors: A Cautionary Tale
Source: J Med Chem. 2022 May 17;65(10):7246–61. doi: 10.1021/acs.jmedchem.2c00228 (PMC9150122; doi:10.1021/acs.jmedchem.2c00228)
Supplement: Supplementary file 1 — jm2c00228_si_001.pdf [file jm2c00228_si_001.pdf]

Supporting Information

Biophysical Survey of Small Molecule β-catenin Inhibitors: A Cautionary Tale

Michael A. McCoy[a], Dominique Spicer[a], Neil Wells, Kurt Hoogewijs[b], Marc Fiedler[c], Matthias G. J. Baud[a*]

[a]School of Chemistry, University of Southampton, Southampton, SO17 1BJ, United Kingdom

[b]National University of Ireland, University Road, H91 TK33 Galway, Ireland

[c]Medical Research Council, Laboratory of Molecular Biology, Francis Crick Avenue, Cambridge, CB2 0QH, United Kingdom

*Correspondence: m.baud@soton.ac.uk

Table of Contents

General Experimental

General Procedures

Synthetic Chemistry

DSF curves

ITC traces

NMR spectra and HPLC traces

References

General Experimental. Chemicals and solvents were bought from commercial suppliers and used as supplied unless otherwise indicated. Reactions were performed using anhydrous solvents, under an inert atmosphere (dry N_2 or Ar). All reactions were evacuated using rotary vane pumps and back-filled with inert atmosphere three times, unless otherwise stated. Reactions were monitored by analytical thin layer chromatography (TLC), NMR ($^1H/^{19}F$) and/or liquid chromatography-mass spectrometry (LC-MS). TLC was performed using 5 cm × 3 cm aluminium TLC plates, silica gel 60 coated with fluorescent indicator F254 (Merck). TLC plates were visualized initially using UV light (254/350 nm) and non-destructive iodine staining. Finally, spots were visualised using an appropriate destructive stain (generally; potassium permanganate, vanillin, bromocresol green or ninhydrin), followed by heating. Solvents were removed by rotary evaporator below 40 ˚C and the compounds further dried using high vacuum pumps. Products were purified using extractions, precipitations, triturations, recrystallization or by flash column chromatography on silica gel (60 Å pore size, 35-60 mesh particle size) or using a Biotage Isolera One with Biotage® Sfär Silica D columns. ^1H, ^{13}C and ^{19}F NMR spectra were recorded on a Bruker Advance III HD FT-NMR spectrometer equipped with an Ascend™ 400 magnet at 400 MHz, 101 MHz and 376 MHz respectively. Chemical shifts (δ) are quoted in ppm (parts per million) and referenced to residual solvent signals ^1H δ = 7.26 ($CDCl_3$), 2.50 ($(CD_3)_2SO$), 3.31 (CD_3OD), 2.05 ($(CD_3)_2CO$), 2.04 (CD_3CO_2D); ^{19}F NMR spectra were referenced externally to $CFCl_3$; ^{13}C δ = 77.16 ($CDCl_3$), 39.51 ($(CD_3)_2SO$), 49.15 (CD_3OD), 29.32 ($(CD_3)_2CO$), 20.00 (CD_3CO_2D). Coupling constants (*J*) are given in Hz. High-resolution mass spectra were recorded using electrospray ionisation on a Bruker SolariX FT-ICR mass spectrometer equipped with a 4.7 T magnet or using electron impact ionisation on a LECO HRT+ time-of-flight mass spectrometer. All compounds were ≥ 95% pure by HPLC analysis.

Differential scanning fluorimetry (DSF). DSF experiments were performed on a Bio-rad CFX Connect Real-time qPCR system, using SYPRO Orange (Life Technologies) as a reporter dye to monitor protein denaturation. In brief: compounds were pre-plated in DMSO at a stock concentration of 2.5 mM and stored at -20°C when not in use. A "mastermix" was prepared with assay buffer (25 mM Tris pH 7.4, 200 mM NaCl, 0.06% NaN_3, 1 mM DTT), SYPRO orange (10.52x) and protein (8.42 µM). The mastermix (23.75 µL) was added to compound stocks in DMSO (1.25 µL) pre-plated on a 96-wp. Final concentrations: SYPRO orange (10x), protein (8 µM), compound (125 µM) and DMSO concentration 5% (v/v). The resulting samples (20 µL) were transferred to a Bio-Rad Hard-Shell® 96-Well PCR Plate (HSP9655), and sealed with Microseal 'B' PCR Plate Sealing Film (MSB1001). The temperature was raised (x°C to x°C over x minutes) and the time dependent fluorescence recorded. The time dependant fluorescence data were analysed using GraphPad Prism and the melting temperature (T_m) of individual sample wells was determine as the maximum of the first derivative of the data. The compound

induced thermal stabilisation (ΔT_m) values were calculated as $\Delta T_m = T_m$ (protein + compound) − T_m (protein). All DSF experiments were performed in triplicate.

Isothermal titration calorimetry (ITC). ITC experiments were conducted using a MicroCal iTC200 calorimeter. All forward titrations were carried out using flash frozen protein, which was thawed and diluted in gel filtration buffer to the required concentration (25 – 50 µM) with 5% (v/v) DMSO. The reference cell contained gel filtration buffer with 5% (v/v) final DMSO content. The titration syringe contained compound (0.125 – 5 mM) in the same gel filtration buffer with 5% (v/v) final DMSO content. Titrations were performed at 25 °C, with the reference power set to 6 µcal/sec, stirring speed set to 1000 rpm and an initial 60 second delay. Titrations were performed over 20 injections (1st injection; Volume = 0.5 µL, 1 sec duration, 120 sec spacing, 5 sec filter period. 2nd – 20th injection; Volume = 2 µL injection, 1 sec duration, 120 sec spacing, 5 sec filter period) and analyzed using MicroCal PEAQ-ITC Analysis software. The initial injection was discarded during data analysis. The data was fitted to a single binding site model to yield enthalpies of binding (ΔH) and binding/dissociation constants (K_as/K_ds). Further thermodynamic parameters i.e. changes in entropy ΔS, changes in free energy ΔG and dissociation constants (Kds) were calculated from these values.

Control Peptide Synthesis. For peptide **1** (TCF4 15-mer) and peptide **2** (BCL9 29-mer), synthesis was performed on 50 µmole ChemMatrix Rink amide resin (loading 0.4 – 0.6 mmol/g) using an Intavis Multipep RSi peptide synthesizer with DMF as solvent. Couplings were performed using 5 equivalents Fmoc-protected amino acid, 5 equivalents HBTU and 10 equivalents DIPEA (from 2M in NMP) for 2 × 40 min for peptide **1**. For peptide **2**, double couplings with 5 equivalents Fmoc-protected amino acid, 5 equivalents DIC, 5 equivalents Oxyma were performed. For DIC/Oxyma couplings, the amino acid was pre-activated for 5 minutes, and coupled for 30 minutes. After coupling of each amino acid, before Fmoc-deprotection, the resin was washed and capped with 5% acetic anhydride, 5% NMM in DMF for 5 min. Fmoc deprotection was performed using 40% piperidine for 4 min, and 20% piperidine for 12 minutes in case of peptide **1**. In case of peptide **2**, 20% piperidine with 0.1M Oxyma was used (to prevent aspartimide formation) for 4 and 10 min. After the final Fmoc-deprotection, peptides were capped, and the resin was washed with DMF (3x), DCM (3x) and Et_2O (3x) and dried *in vacuo*. The resin was subsequently treated with 20mL TFA/DODT/TIS/H_2O (92.5/2.5/2.5/2.5) for 3 h, filtered and the cleavage solution was concentrated to 2 mL. The peptide was precipitated using MTBE (-18 °C) and centrifuged for 5 min at 5000 RPM. The precipitate was washed 2 more times with MTBE and dried *in vacuo*. Finally, the crude peptide was dissolved in 30% MeCN/H_2O 0.1% TFA, and purified by RP-HPLC (Gilson LC2250, Waters Delta-Pak C18 100 Å column in a Waters 1000 PrepPAK Module, 60 mL/min 0 – 100% B gradient, eluent A = 0.1% TFA in H_2O, B = 0.1% TFA in MeCN), lyophilized and stored at -20 °C. Final analysis of the peptides was performed by LC-MS (Agilent 1100 Series instrument,

Phenomenex Kinetex C18 100 Å column (150 × 4.6 mm, 5 µm at 35 °C) connected to an ESMSD type VL mass detector with a flow rate of 1.5 ml/min, 0.1% formic acid in elution buffer, 1 min 0% MeCN, 0 – 100% MeCN in 6 minutes, 2 min 100% MeCN).

Peptide 1 – TCF 15-mer

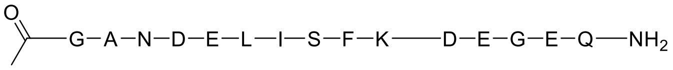

Chemical Formula: $C_{71}H_{109}N_{19}O_{29}$
Exact Mass: 1691.7639
Molecular Weight: 1692.7570

LC-MS. ESI-MS in neg mode

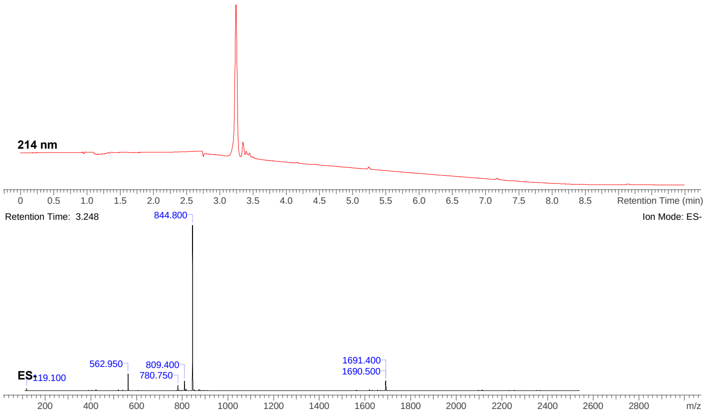

Figure S1 – Sequence of peptide 1 and corresponding HPLC-MS trace

Peptide 2 – BCL9 29-mer

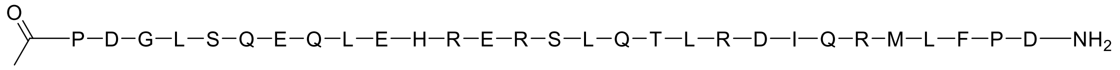

Chemical Formula: $C_{151}H_{246}N_{48}O_{49}S$
Exact Mass: 3547.7954
Molecular Weight: 3549.9760
m/z: 3548.7988 (100.0%), 3549.8021 (81.1%), 3547.7954 (61.2%)

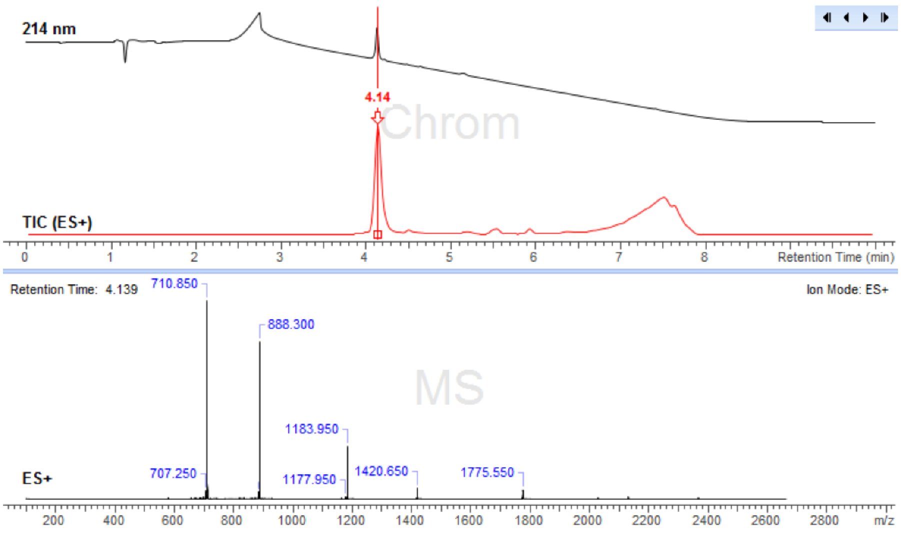

Figure S2 – Sequence of peptide 2 and corresponding HPLC-MS trace

Table S1 – Ionisation calculator for peptide 2

Calculated exact mass, 100% peak, z = 0 => 3548.80

Charge (z)	Calculated m/z	Measured m/z
5	710.76	710.85
4	888.20	888.30
3	1183.93	1183.95
2	1775.40	1775.55

Generation of plasmids, β-catenin-ARD protein expression and purification. Human β-catenin sequences were amplified by PCR (KOD DNA polymerase, Merck Millipore) from plasmid templates and cloned into bacterial expression vectors by restriction-free cloning. All plasmids were verified by sequencing. For protein production, bacterial expression vectors pLipK-ARD (150 – 662) and pLipK-ARDext (148 – 662) were used. 6xHisLip-tagged recombinant proteins were purified from BL21(DE3) pRARE2 E. coli bacterial strains. Bacteria were grown in LB media supplemented with appropriate antibiotic to OD_{600} 0.6, then dropped to a lower temperature (16 – 24 °C) and induced at OD_{600} 0.8 by addition of 0.4 mM isopropyl β-D-1-thiogalactopyranoside (IPTG). Proteins were expressed overnight and bacteria were subsequently harvested by centrifugation, cell pellets shock-frozen in liquid nitrogen and stored at -80°C until use. Cell pellets were re-suspended in lysis buffer (25 mM Tris-HCl pH 8, 200 mM NaCl, 20 mM imidazole, 10 µg/mL DNAse, EDTA-free protease inhibitor cocktail) and lysed with an Emulsiflex C-3 (Avestin). Lysates were cleared by ultracentrifugation (140,000x rcf, 30 minutes, 4°C) and mixed with Ni-NTA agarose. Beads were washed multiple times with lysis buffer, and 6xHisLip-tagged protein was eluted with lysis buffer supplemented with 500 mM imidazole. Each protein was further purified by SEC on a HiPrep26/60 G200 (GE Healthcare) into the final buffer of 200 mM NaCl, 25 mM Tris pH 7.4, 0.06% NaN_3, 1 mM DTT, and protein purity was assessed by SDS-PAGE. Pure protein fractions were concentrated using a 10 kDa MWCO Vivaspin 20 concentrator (Sartorius) to 17 – 28 mg/mL, then shock frozen as droplets in liquid nitrogen and stored at -80°C until use.

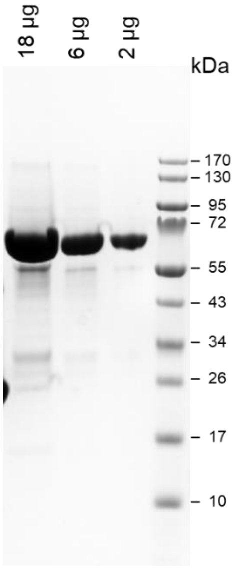

Figure S3 – SDS page gel of purified pLipK-ARD (150 – 662)

Synthetic Chemistry

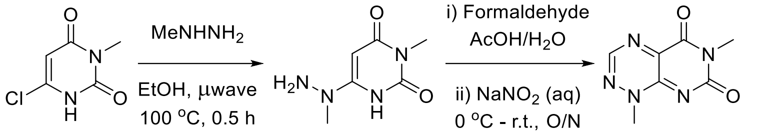

40 **41**, (500 mg, 47%) **10** (115 mg, 34%)

Scheme S1

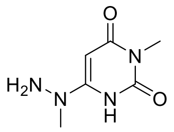

41

3-methyl-6-(1-methylhydrazineyl)pyrimidine-2,4(1*H*,3*H*)-dione, 41 – Following the procedure published by Todorovic *et al.*, **40** (1 g, 6.25 mmol, 1 eq.) was suspended in EtOH (10 mL) and *N*-methylhydazine (985 µL, 18.75 mmol, 3 eq.) in a sealed microwave vial.[1] The reaction was heated to 100 °C for 0.5 h in a microwave reactor. The reaction was cooled on ice for 1 hour and the resulting precipitate was isolated *via* vacuum filtration and washed with cold EtOH to afford **41** (500 mg, 47%) as a white solid which was used without further purification. **R_f** = 0.3; **^{1}H NMR (400 MHz, $(CD_3)_2SO$):** δ ppm 4.66 (s, 1H) 3.04 (s, 3H) 3.02 (s, 3H); **^{13}C{^{1}H} NMR (101 MHz, $(CD_3)_2SO$):** δ ppm 162.9, 153.9, 150.4, 72.3, 40.3, 26.0; **HRMS (m/z):** $[M+H]^+$ calcd. 171.0877 for $C_6H_{11}N_4O_2$; found 171.0877.

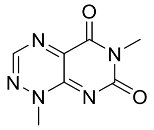

10

1,6-dimethylpyrimido[5,4-*e*][1,2,4]triazine-5,7(1*H*,6*H*)-dione, 10 – Following the procedure by Mao *et al.*, **41** (300 mg, 1.76 mmol, 1eq.) was degassed, purged with argon and dissolved in degassed AcOH:H_2O (9:1, 5 mL).[2] The reaction was cooled to 4 °C and aqueous formaldehyde (37%, 157 µL, 1.94 mmol, 1.1 eq) was added dropwise. Upon complete consumption of **41** (0.5 h, TLC), $NaNO_2$ (134 mg, 1.94 mmol, 1.1 eq) in degassed H_2O (2 mL) was added dropwise to the reaction mixture. The reaction instantly turned yellow and was stirred at 4 °C for a further 10 minutes before being warmed to r.t., and stirred O/N. The reaction mixture was concentrated under reduced pressure and purified *via* column chromatography to afford **10** (115 mg, 34%) as a yellow solid. **R_f** = 0.2 (DCM/IPA 96:4); **^{1}H NMR (400 MHz, $CDCl_3$):** δ 8.81 (s, 1H), 4.15 (s, 3H), 3.50 (s, 3H); **^{13}C{^{1}H} NMR (101 MHz, $CDCl_3$):** δ 158.6, 154.4, 150.6, 145.7, 145.4, 43.5, 29.3; **HRMS (m/z):** $[M+H]^+$ calcd. 194.0673 for $C_7H_8N_5O_2$; found 194.0675; $[M+Na]^+$ calcd. 216.0492 for $C_7H_7N_5NaO_2$; found 216.0494.

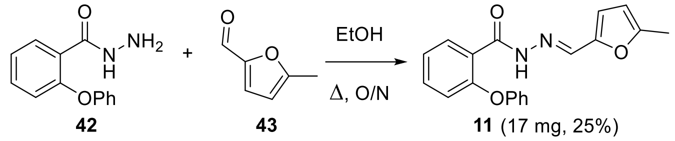

Scheme S2

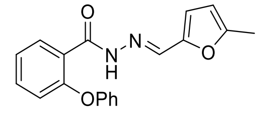

11

***N*'-((5-methylfuran-2-yl)methylene)-2-phenoxybenzohydrazide, 11 - 42** (100 mg, 0.44 mmol, 1 eq.) was dissolved in ethanol (5 mL) and purged with argon. **43** (44 µL, 0.44 mmol, 1 eq.) was added and the reaction was refluxed O/N. The reaction was monitored *via* TLC and concentrated under reduced pressure upon consumption of starting material. The crude product was purified *via* column chromatography to afford **11** (122 mg, 87%) as a white solid. **R_f** = 0.30 (DCM/EtOAc 95:5); **^{1}H NMR (400 MHz, $(CD_3)_2SO$, E/Z mixture):** δ ppm 11.65 – 11.56 (m, 1H), 8.13 – 7.76 (m, 1H), 7.58 – 7.32 (m, 3H), 7.31 – 7.00 (m, 5H), 6.99 – 6.79 (m, 1H), 6.79 – 6.59 (m, 1H), 6.28 – 6.15 (m, 1H), 2.36 – 2.28 (m, 3H); **$^{13}C\{^{1}H\}$ NMR (101 MHz, $(CD_3)_2SO$, E/Z mixture)** δ 168.7, 161.8, 156.5, 156.3, 154.6, 154.2, 153.7, 147.8, 147.4, 137.0, 134.0, 132.0, 130.8, 130.0 (2C), 129.9, 129.6, 129.0, 127.9, 127.4, 123.7, 123.6, 122.9, 119.3, 119.1, 118.6 (2C), 117. 5, 115.5, 115.4, 108.6, 108.4, 13.5 (2C); **HRMS:** (ESI-TOF, m/z) calcd. for $C_{19}H_{17}N_2O_3$ $[M+H]^+$ calc: 321.1236. found: 321.1234; for $C_{19}H_{16}N_2NaO_3$ $[M+Na]^+$ calc: 343.1058. found: 343.1053.

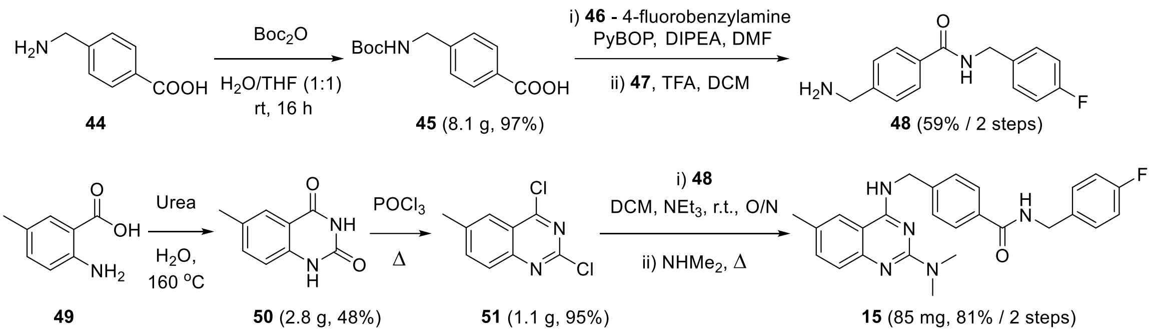

Scheme S3

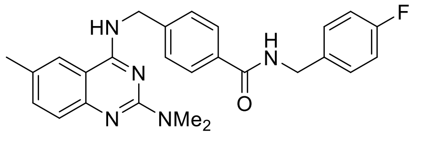

15

4-(((2-(dimethylamino)-6-methylquinazolin-4-yl)amino)methyl)-*N*-(4-fluorobenzyl)benzamide, 15 – Made according to the literature procedure.[3] **R_f** = 0.3 (DCM/MeOH/NH_4OH 95:4:1); **^{1}H NMR (400 MHz, $(CD_3)_2SO$):** δ ppm 8.99 (t, *J* = 6.1 Hz, 1H) 8.61 (br t, *J* = 5.3 Hz, 1H) 7.87 (s, 1H) 7.86 – 7.82 (m, 2H) 7.46 (d, *J* = 8.4 Hz, 2H) 7.37 – 7.31 (m, 3H) 7.23 (d, *J* = 8.6 Hz, 1H) 7.13 (t, *J* = 8.9 Hz, 2H) 4.73 (d, *J* = 5.7 Hz, 2H) 4.44 (d, *J* = 6.0 Hz, 2H) 3.05 (s, 6H) 2.36 (s, 3H); **^{19}F{^{1}H} NMR (376 MHz, $(CD_3)_2SO$):** δ ppm -116.1 (s, 1F); **^{13}C{^{1}H} NMR (101 MHz, $(CD_3)_2SO$):** δ ppm 166.1, 161.1 (d, *J* = 242 Hz), 159.1, 158.3, 143.6, 135.9 (d, *J* = 2.9 Hz), 134.1, 132.7, 129.3, 129.2 (d, *J* = 8.1 Hz, 2C), 127.2 (s, 2C) 127.1 (s, 2C), 124.3, 121.9, 114.9 (d, *J* = 21 Hz, 2C), 109.8, 69.8, 43.7, 41.9, 36.6 (s, 2C) 20.8; **HRMS (m/z):** $[M+H]^+$ calcd. 444.2194 For $C_{26}H_{27}FN_5O$; found 444.2205.

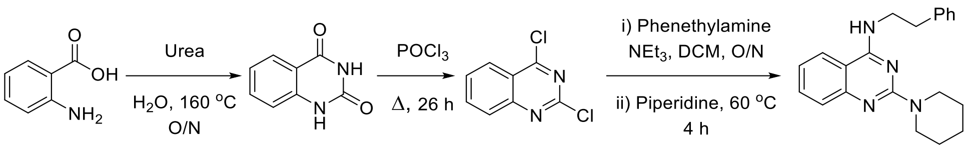

52 **53** (1.01 g, 43%) **54** (1.05 g, 91%) **16** (286 mg, 86%)

Scheme S4

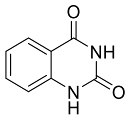

53

Quinazoline-2,4(1*H*,3*H*)-dione, 53 – Urea (9.1 g, 152 mmol, 10.4 eq.) was heated to 170 °C and **52** (2.0 g, 14.6 mmol, 1 eq.) was added in one portion. The reaction was stirred for 0.5 minutes after which it solidified. The reaction was cooled to 100 °C, diluted in water (20 mL) and stirred O/N. The resultant precipitate was collected *via* vacuum filtration, washed with water and dried under vacuum to afford **53** (1.01 g, 43%) as a white solid which was used without further purification. **^{1}H NMR (400 MHz, $(CD_3)_2SO$)** δ ppm 11.27 (br s, 1H) 11.12 (br s, 1H) 7.88 (dd, *J* = 8.1, 0.9 Hz, 1H) 7.66 – 7.59 (m, 1H) 7.20 – 7.14 (m, 2H); **^{13}C{^{1}H} NMR (101 MHz, $(CD_3)_2SO$)** δ ppm 162.8, 150.3, 140.7, 135.0, 127.0, 122.3, 115.3, 114.3; **HRMS (m/z):** $[M+H]^+$ calcd. 163.0502 For $C_8H_7N_2O_2$; found 163.0505.

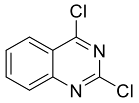

54

2,4-Dichloroquinazoline, 54 – **53** (943 mg, 5.82 mmol, 1.0 eq.) was suspended in $POCl_3$ (11 mL, 116 mmol, 20 eq.) and heated to reflux for 26 hours after which was cooled to r.t., and concentrated under reduced pressure. The crude residue was triturated in ice cold water (10 mL) and the precipitate collected *via* vacuum filtration to afford **54** (1.05 g, 91%) as an off-white solid which was used without further purification. **^{1}H NMR (400 MHz, $CDCl_3$)** δ ppm 8.26 (dt, *J* = 8.3, 1.0 Hz, 1H), 8.03 – 7.96 (m, 2H), 7.81 – 7.68 (m, 1H); **^{13}C{^{1}H} NMR (101 MHz, $CDCl_3$)** δ ppm 164.0, 155.1, 152.3, 136.1, 129.2, 128.0, 126.0, 122.3; **HRMS (m/z):** $[M+H]^+$ calcd. 198.9824 For $C_8H_5Cl_2N_2$; found 198.0923.

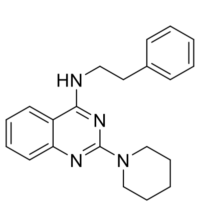

16

***N*-phenethyl-2-(piperidin-1-yl)quinazolin-4-amine, 16** – **54** (200 mg, 1.0 mmol, 1.0 eq.) was evacuated and filled with argon thrice. AHS DCM (10 mL) was added followed by NEt_3 (292 µL, 2.1

mmol, 2.1 eq.) and phenethylamine (132 µL, 1.05 mmol, 1.05 eq.). The reaction was stirred at r.t., O/N, then concentrated under reduced pressure. The residue was suspended in anhyd. THF (5 mL), piperidine (5 mL, 50 mmol, 50 eq.) was added and the reaction was heated to 60 °C and monitored by TLC. The reaction was complete within 3 h, concentrated and purified by column chromatography to afford **16** (286 mg, 86%) as a white solid. **R_f** = 0.2 (DCM/MeOH 95:5); **^{1}H NMR (400 MHz, CD_3OD):** δ ppm 8.05 – 8.02 (m, 1H), 7.77 (ddd, *J* = 8.5, 7.2, 1.3 Hz, 1H), 7.63 – 7.59 (m, 1H), 7.41 (ddd, *J* = 8.3, 7.2, 1.2 Hz, 1H), 7.30 – 7.16 (m, 5H), 3.93 – 3.86 (m, 6H), 3.04 (t, *J* = 7.3 Hz, 2H), 1.87 – 1.69 (m, 6H); **^{13}C{^{1}H} NMR (101 MHz, CD_3OD):** δ ppm 161.3, 153.0, 141.2, 140.4, 136.3, 130.0 (2C), 129.8 (2C), 127.7, 126.2, 124.6, 118.6, 111.4, 47.5 (2C), 44.6, 36.0, 27.0 (2C), 25.3; **HRMS (m/z):** $[M+H]^+$ calcd. 333.2074 For $C_{21}H_{25}N_4$; found 333.2076.

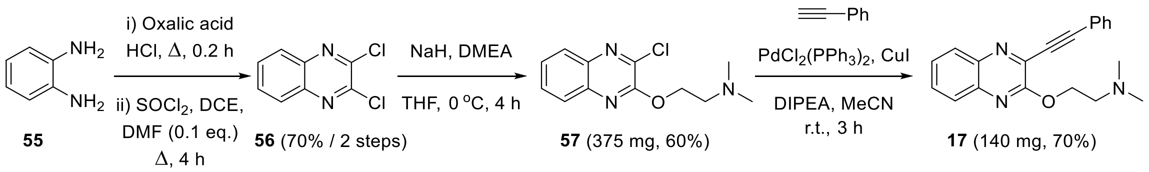

Scheme S5

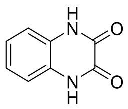

56i

1,4-dihydroquinoxaline-2,3-dione, 56i – H_2O (15 mL) was acidified with conc. HCl (2mL) and heated to 80 °C. Oxalic acid (1.4 g, 11.1 mmol, 1.2 eq.) was added in one portion followed by **55** (1.0 g, 9.3 mmol, 1.0 eq.) and the reaction was heated to 100 °C. After 0.2 h, a precipitate formed which was isolated *via* vacuum filtration and dried under high vacuum to afford **56i**, (1.0 g, 70%) as silver needles which were used without further purification. **R_f** = 0.2 (DCM/Acetone 9:1); **^{1}H NMR (400 MHz, $(CD_3)_2SO$):** δ 11.90 (s, 2H), 7.15 – 7.05 (m, 4H); **$^{13}C\{^1H\}$ NMR (101 MHz, $(CD_3)_2SO$):** δ 155.2 (2C), 125.6 (2C), 123.0 (2C), 115.1 (2C); **HRMS (m/z):** $[M+H]^+$ calcd. 163.0502 For $C_8H_7N_2O_2$; found 163.0498.

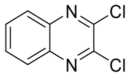

56

2,3-dichloroquinoxaline, 56 – **56i** (1.0 g, 6.2 mmol, 1 eq.) was suspended in anhydrous DCE (25 mL). $SOCl_2$ (0.9 mL, 12.4 mmol, 2 eq.) was added followed by DMF (48 µL, 0.62 mmol, 0.1 eq.). The reaction was purged with argon, heated to 90 °C for 4 hours, and then cooled to room temperature. The reaction was concentrated under reduced pressure to afford **56** (1.23 g, quant.) as white crystals which were used without further purification. **R_f** = 0.9 (DCM/MeOH 95:5); **^{1}H NMR (400 MHz, $(CD_3)_2SO$):** δ 8.09 (m, 2H), 7.95 (m, 2H); **$^{13}C\{^1H\}$ NMR (101 MHz, $(CD_3)_2SO$):** δ 144.6 (2C), 140.0 (2C), 131.8 (2C), 127.9 (2C).

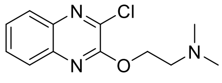

57

2-((3-chloroquinoxalin-2-yl)oxy)-*N*,*N*-dimethylethan-1-amine, 57 – NaH (104 mg, 2.6 mmol, 1.05 eq.) was evacuated and filled with argon thrice and dissolved in AHS THF (9 mL). The reaction was chilled to 0 °C and *N*,*N*-dimethylethanolamine (DMEA) (250 µL, 2.5 mmol, 1 eq.) was added dropwise. H_2 evolution was observed and the reaction was stirred at 0 °C for 0.5 h. **56** (500 mg, 2.5 mmol, 1 eq.) was evacuated and filled with argon thrice, dissolved in AHS THF (3 mL) and added to the reaction containing DMEA. The reaction was warmed to r.t., stirred for 4 hours and monitored *via* TLC. The

reaction was concentrated, suspended in H_2O (30 mL) and neutralised with sat. NH_4Cl. The aqueous layer was extracted with ethyl acetate (4 x 30 mL). The combined organic layers were washed with sat. brine (30 mL), dried with $MgSO_4$, filtered and concentrated under reduced pressure. The crude material was purified by column chromatography to afford **57** (375 mg, 60%) as a white solid. $\mathbf{R_f}$ = 0.2 (DCM/MeOH 95:5); **^{1}H NMR (400 MHz, $CDCl_3$):** δ 7.90 (dd, *J* = 8.2, 1.1 Hz, 1H), 7.80 (dd, *J* = 8.4, 1.0 Hz, 1H), 7.64 (ddd, *J* = 8.3, 7.0, 1.4 Hz, 1H), 7.55 (ddd, *J* = 8.3, 7.0, 1.5 Hz, 1H), 4.65 (t, *J* = 5.8 Hz, 2H), 2.85 (t, *J* = 5.8 Hz, 2H), 2.39 (s, 6H); **^{13}C{^{1}H} NMR (101 MHz, $CDCl_3$):** δ 153.1, 139.6, 138.4, 130.3, 128.1, 127.5, 126.9, 66.1, 57.7, 46.1 (2C); **HRMS (m/z):** $[M+H]^+$ calcd. 252.0898 For $C_{12}H_{15}ClN_3O$; found 252.0899.

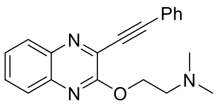

17

***N*,*N*-dimethyl-2-((3-(phenylethynyl)quinoxalin-2-yl)oxy)ethan-1-amine, 17** – **57** (150 mg, 0.6 mmol, 1 eq.), $PdCl_2(PPh_3)_2$ (21 mg, 0.03 mmol, 0.05 eq.), CuI (5.7 mg, 0.03 mmol, 0.05 eq.) were degassed and purged with argon in a sealed vial. Degassed anhydrous MeCN (2 mL) was added to the vial, followed by DIPEA (313 µL, 1.8 mmol, 3 eq.) and phenylacetylene (197 µL, 1.8 mmol, 3 eq.). The reaction was stirred at r.t., and monitored by TLC. After 3 hours, the reaction was filtered over celite, concentrated under reduced pressure and purified *via* column chromatography to afford **17** (140 mg, 70%) as a beige solid. $\mathbf{R_f}$ = 0.2 (DCM/MeOH 95:5); **^{1}H NMR (400 MHz, $(CD_3)_2SO$)**: ppm 8.02 (d, *J* = 7.9 Hz, 1H), 7.90 – 7.84 (m, 1H), 7.80 (t, *J* = 7.2 Hz, 1H), 7.74 – 7.66 (m, 3H), 7.60 – 7.47 (m, 3H), 4.87 (br s, 2H), 3.76 – 3.46 (m, 2H), 2.89 (br s, 6H); **^{13}C{^{1}H} NMR (101 MHz, $(CD_3)_2SO$)**: δ ppm 155.7, 138.5, 138.3, 132.0 (2C), 131.9, 131.3, 130.3, 129.1 (2C), 128.4, 127.8, 126.7, 120.7, 95.0, 84.9, 62.5, 55.2, 43.3 (2C); **HRMS (m/z)**: $[M+H]^+$ calcd. 318.1601 For $C_{20}H_{20}N_3O$; found 318.0602.

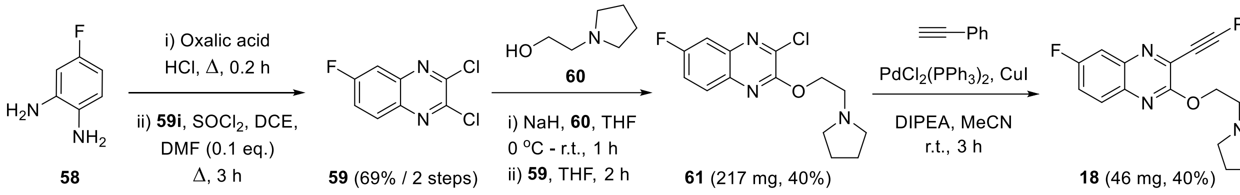

Scheme S6

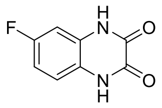

59i

6-fluoro-1,4-dihydroquinoxaline-2,3-dione, 59i – H_2O (15 mL) was acidified with conc. HCl (2 mL) and heated to 80 °C. Oxalic acid (1.4 g, 11.1 mmol, 1.2 eq.) was added in one portion followed by **58** (1.16 g, 9.3 mmol, 1.0 eq.). The reaction was heated to 100 °C. After 0.2 h, a precipitate formed which was isolated *via* vacuum filtration and dried under high vacuum to afford **59i**, (1.36 g, 81%) as grey needles which were used without further purification. **R*f*** = 0.2 (DCM/Acetone 9:1); **^{1}H NMR (400 MHz, $(CD_3)_2SO$):** δ ppm 11.95 (s, 1H) 11.91 (s, 1H) 7.11 (dd, *J* = 8.8, 5.3 Hz, 1H) 6.97 – 6.86 (m, 2H); **^{19}F{^{1}H} NMR (376 MHz, $(CD_3)_2SO$):** δ ppm -119.6 (s, 1F); **^{13}C{^{1}H} NMR (101 MHz, $(CD_3)_2SO$)**: δ ppm 157.7 (d, *J* = 238 Hz) 155.2, 154.6, 126.7 (d, *J* = 12 Hz), 122.4 (d, *J* = 2.2 Hz) 116.4 (d, *J* = 9.5 Hz) 109.8 (d, *J* = 24 Hz) 101.8 (d, *J* = 27 Hz); **HRMS (m/z):** $[M+H]^+$ calcd. 181.0408 For $C_8H_6FN_2O_2$; found 181.0408.

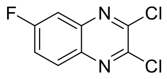

59

2,3-dichloro-6-fluoroquinoxaline, 59 – **59i** (556 mg, 3.1 mmol, 1 eq.) was suspended in anhydrous DCE (12 mL). $SOCl_2$ (0.45 mL, 6.2 mmol, 2 eq.) was added followed by DMF (24 µL, 0.31 mmol, 0.1 eq.). The reaction was purged with argon and heated to 90 °C for 3 hours. The reaction was cooled, unreacted **59i** was removed by filtration and the filtrate was diluted in MeOH (50 mL), and quenched with sat. $NaHCO_3$. The resulting precipitate was collected *via* vacuum filtration and washed with cold water to afford **59** (568 mg, 85%) as pink crystals which were used without further purification. **R*f*** = 0.2 (Hex/EA 9:1); **^{1}H NMR (400 MHz, $(CD_3)_2SO$)**: δ ppm 8.19 (dd, *J* = 9.2, 5.7 Hz, 1H), 7.97 (dd, *J* = 9.2, 2.8 Hz, 1H) 7.93 – 7.86 (m, 1H); **^{19}F{^{1}H} NMR (376 MHz, $(CD_3)_2SO$):** δ ppm -105.81 (s, 1 F); **^{13}C{^{1}H} NMR (101 MHz, $(CD_3)_2SO$):** δ ppm 163.3 (d, *J* = 252 Hz), 146.3, 144.6 (d, *J* = 3.7 Hz), 141.4 (d, *J* = 14 Hz), 137.9, 131.0 (d, *J* = 11 Hz), 122.2 (d, *J* = 26 Hz), 112.5 (d, *J* = 23 Hz).

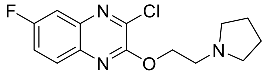

61

3-chloro-6-fluoro-2-(2-(pyrrolidin-1-yl)ethoxy)quinoxaline, 61 – NaH (77 mg, 1.93 mmol, 1.05 eq.) was evacuated and filled with N_2 thrice, cooled to 0 °C and suspended in anhydrous THF (5 mL). **60** (226 µL, 1.93 mmol, 1.05 eq.) was added dropwise and the reaction was stirred at 0 °C for 1 hour. **59** (400 mg, 1.84 mmol, 1 eq.) was evacuated and filled with N_2 thrice, cooled to 0 °C and dissolved in anhydrous THF (12 mL). The solution containing **59** was added dropwise to **60** at 0 °C. The reaction was warmed to r.t., monitored by TLC and complete after 2 hours. The reaction was quenched with AcOH (0.2 mL), concentrated under reduced pressure and purified by column chromatography to afford **61** (217 mg, 40%) as a beige solid. **R_f** = 0.3 (DCM/acetone 3:7); **^{1}H NMR (400 MHz, $CDCl_3$):** δ ppm 7.91 (dd, *J* = 9.2, 5.7 Hz, 1H), 7.47 (dd, *J* = 9.2, 2.8 Hz, 1H), 7.35 (ddd, *J* = 9.2, 8.2, 2.8 Hz, 1H), 4.82 – 4.79 (m, 2H), 3.33 – 3.29 (m, 2H), 3.13 – 3.04 (m, 4H), 1.97 (m, 4H); **^{19}F{^{1}H} NMR (376 MHz, $CDCl_3$):** δ ppm -108.0 (s, 1F); **^{13}C{^{1}H} NMR (101 MHz, $CDCl_3$)**: δ ppm 176.1, 163.2 (d, *J* = 252 Hz), 152.9, 140.5 (d, *J* = 13 Hz), 138.3 (d, *J* = 2.9 Hz), 129.8 (d, *J* = 10 Hz), 117.4 (d, *J* = 25 Hz), 111.3 (d, *J* = 23 Hz), 64.9, 54.1 (2C), 53.3, 23.3 (2C)**; HRMS (m/z):** $[M+H]^+$ calcd. 296.0960 For $C_{14}H_{16}ClFN_3O$; found 296.0952.

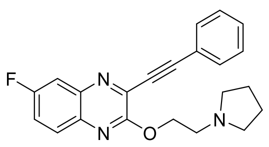

18

3-chloro-6-fluoro-2-(2-(pyrrolidin-1-yl)ethoxy)quinoxaline, 18 – **61** (95 mg, 0.32 mmol, 1 eq.), $PdCl_2(PPh_3)_2$ (11.3 mg, 0.016 mmol, 0.05 eq.) and CuI (3 mg, 0.016 mmol, 0.05 eq.) were evacuated and filled with Ar thrice. Anhydrous MeCN (3.2 mL) was added followed by DIPEA (168 µL, 0.966 mmol, 3 eq.) and phenylacetylene (106 µL, 0.966 mmol, 3 eq.). The reaction was stirred at r.t., and monitored for 24 h until completion. The reaction was concentrated, re-suspended in ethyl acetate (10 mL) and filtered through a celite pad. The filtrate was concentrated and purified by column chromatography to afford **18** (46 mg, 40%) as a beige solid. **R_f** = 0.2 (DCM/Acetone 1:1); **^{1}H NMR (400 MHz, $(CD_3)_2SO$):** δ ppm 8.03 (ddd, *J* = 8.5, 6.1, 1.0 Hz, 1H), 7.68 – 7.62 (m, 2H), 7.60 – 7.48 (m, 5H), 4.58 (t, *J* = 5.5 Hz, 2H), 2.93 (br t, *J* = 5.0 Hz, 2H), 2.64 (br s, 4H), 1.67 (dt, *J* = 6.7, 3.2 Hz, 4 H); **^{19}F{^{1}H} NMR (376 MHz, $(CD_3)_2SO$):** δ ppm -107.2 (s, 1F); **^{13}C{^{1}H} NMR (101 MHz, $(CD_3)_2SO$)**: δ ppm 163.0 (d, *J* = 249 Hz), 156.9, 140.0 (d, *J* = 14 Hz), 135.3, 131.9 (2C), 131.4 (d, *J* = 3.7 Hz), 130.7 (d, *J* = 11 Hz), 130.2, 129.0 (2C), 120.8, 116.9 (d, *J* = 25 Hz), 110.9 (d, *J* = 23 Hz), 95.1, 84.8, 66.7, 54.3 (2C), 53.6, 23.2 (2C); **HRMS (m/z):** $[M+H]^+$ calcd. 362.1663 For $C_{22}H_{21}FN_3O$; found 362.1668.

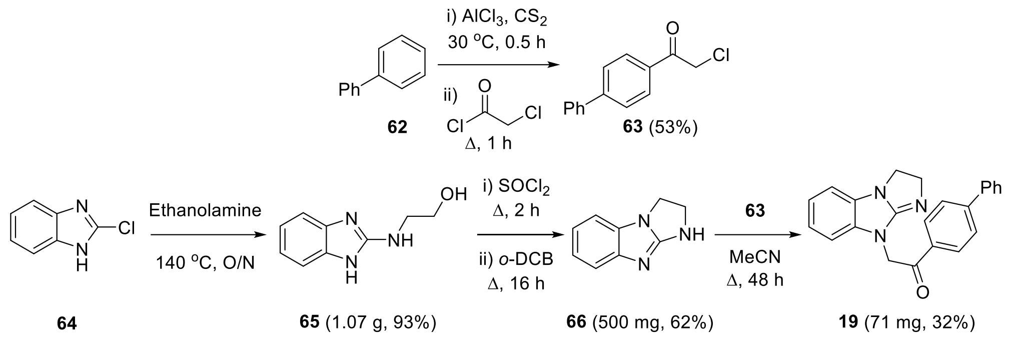

Scheme S7

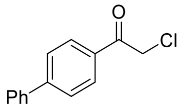

63

1-([1,1'-biphenyl]-4-yl)-2-chloroethan-1-one, 63 – **62** (1 g, 6.5 mmol, 1 eq.) and $AlCl_3$ (863 mg, 6.5 mmol, 1 eq.) were purged with argon and suspended in anhydrous CS_2 (10 mL). Chloroacetyl chloride (0.52 mL, 6.5 mmol, 1 eq.) was diluted in anhydrous CS_2 (10 mL) and added dropwise over 20 minutes *via* dropping funnel. The reaction was heated to reflux during addition and then refluxed for a further hour. The reaction was cooled and slowly added to ice acidified with 1M HCl. The precipitate was collected *via* vacuum filtration, washed with water and triturated in EtOH/H_2O (1:1) to afford **63** (794 mg, 53%) as a yellow solid which was used without further purification. **R_f** = 0.3 (DCM/MeOH 9:1); **^1H NMR (400 MHz, $(CD_3)_2SO$)**: δ ppm 8.09 – 8.05 (m, 2H), 7.88 – 7.83 (m, 2H) 7.79 – 7.73 (m, 2H) 7.55 – 7.49 (m, 2H), 7.48 – 7.40 (m, 1H), 5.23 (s, 2H); **$^{13}C\{^1H\}$ NMR (101 MHz, $(CD_3)_2SO$)**: δ ppm 191.2, 145.2, 138.7, 133.1, 129.10 (2C), 129.07 (2C), 128.5, 127.03 (2C), 126.98 (2C), 47.7; **HRMS (m/z):** $[M+H]^+$ calcd. 231.0571 for $C_{14}H_{12}ClO$; found 231.0570; $[M+Na]^+$ calcd. 253.0391 for $C_{14}H_{11}ClNaO$; found 253.0391.

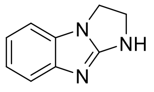

66

2,3-dihydro-1*H*-benzo[*d*]imidazo[1,2-*a*]imidazole, 66 – **64** (1 g, 6.5 mmol, 1 eq.) was purged with argon, suspended in ethanolamine (2 mL, 33 mol, 5 eq.) and heated to 140 °C O/N. The reaction was diluted in sat. $NaHCO_3$ and stirred for 10 minutes, after which the precipitate was collected *via* vacuum filtration and dried under vacuum to afford **65** (1.07 g, 93%) as a white solid which was used without further purification. **65** (900 mg, 5.1 mmol, 1 eq.) was purged with argon and suspended in anhydrous DCE (20 mL). $SOCl_2$ (0.5 mL, 6.9 mmol, 1.3 eq.) was added in one portion and the reaction was heated to reflux for 2 hours. The reaction was cooled, concentrated under reduced pressure, re-dissolved in *o*-dichlorobenzene and heated to reflux O/N. The reaction was concentrated under reduced pressure

and purified *via* column chromatography to afford **66** (500 mg, 62%) as a white solid. **R_f** = 0.3 (DCM/MeOH 9:1); **^{1}H NMR (400 MHz, $(CD_3)_2SO$)**: δ ppm 8.23 (br s, 1H), 7.30 – 7.20 (m, 2H) 7.09 – 7.03 (m, 2H), 4.23 – 4.17 (m, 2H) 4.12 – 4.05 (m, 2H); **^{13}C{^{1}H} NMR (101 MHz, $(CD_3)_2SO$)**: δ ppm 159.4, 142.0, 130.3, 121.4, 120.8, 114.3, 108.8, 48.23, 42.03; **HRMS (m/z):** $[M+H]^+$ calcd. 160.0869 for $C_9H_{10}N_3$; found 160.0871.

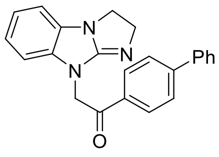

19

1-([1,1'-biphenyl]-4-yl)-2-(2,3-dihydro-9*H*-benzo[*d*]imidazo[1,2-*a*]imidazol-9-yl)ethan-1-one, 19 – **66** (100 mg, 0.63 mmol, 1 eq.) and **63** (188 mg, 0.82 mmol, 1.3 eq.) were purged with argon and dissolved in anhydrous MeCN (5 mL). The reaction was heated to reflux and stirred for 2 days. The reaction was monitored *via* TLC. The reaction was concentrated under reduced pressure and purified *via* column chromatography to afford **19** (71 mg, 32%) as a white solid. **R_f** = 0.3 (DCM/MeOH/NH_4OH 96:3:1); **^{1}H NMR (400 MHz, $CDCl_3$):** δ 8.11 (m, 2H), 7.72 (m, 2H), 7.64 (m, 2H), 7.48 (m, 2H), 7.42 (m, 1H), 6.97 (td, *J* = 7.6, 1.1 Hz, 1H), 6.89 (td, *J* = 7.7, 1.1 Hz, 1H), 6.76 (dd, *J* = 7.5, 0.7 Hz, 1H), 6.67 (dd, *J* = 7.8, 0.6 Hz, 1H), 5.24 (s, 2H), 4.29 (t, *J* = 8.3 Hz, 2H), 3.93 (t, *J* = 8.6 Hz, 2H); **^{13}C{^{1}H} NMR (101 MHz, $CDCl_3$):** δ 191.8, 162.1, 146.8, 139.7, 137.5, 133.3, 131.1, 129.1 (2C), 129.0 (2C), 128.6, 127.6 (2C), 127.4 (2C), 121.7, 120.7, 107.9, 107.2, 57.8, 49.1, 45.6; **HRMS (m/z):** $[M+H]^+$ calcd. 354.1601 For $C_{23}H_{20}N_3O$; found 354.1608.

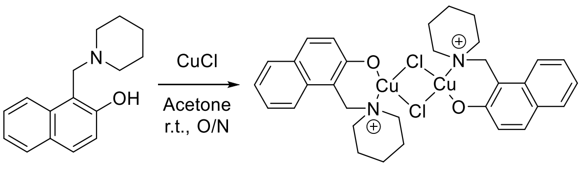

67 **21** (110 mg, 77%)

Scheme S8

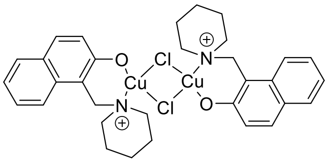

21

NSC109268, 21 – **67** (100 mg, 0.42 mmol, 1 eq.) and CuCl (42 mg, 0.42 mmol, 1 eq.) were evacuated and filled with argon thrice and cooled to 0 °C. Degassed acetone (5 mL) was added and reaction was stirred O/N. The precipitate was isolated *via* vacuum filtration and dried under vacuum to afford **21** (110 mg, 77%) as a brown solid which was used without further purification. **^{1}H NMR (400 MHz, CD_3CO_2D)**: δ ppm 8.23 – 7.39 (m, 12H), 5.36 – 4.62 (br s, 4H), 4.23 – 3.62 (br s, 4H), 3.21 - 3.55 – 3.21 (br s, 4H), 2.15 – 1.63 (m, 12H); **^{13}C NMR (101 MHz, CD_3CO_2D)**: δ ppm 157.1 (2C), 135.5 (2C), 134.2 (2C), 130.9 (2C), 130.7 (2C), 129.6 (2C), 129.2 (2C), 125.4 (2C), 124.7 (2C), 119.9 (2C), 55.9 (4C), 53.7 (2C), 25.4 (4C), 23.4 (2C).

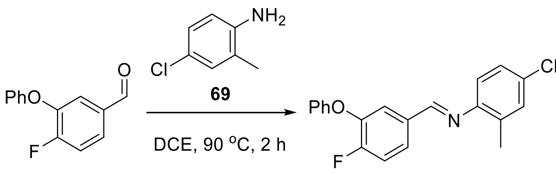

68 **25** (170 mg, 71%)

Scheme S9

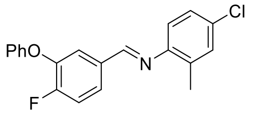

25

***N*-(4-chloro-2-methylphenyl)-1-(4-fluoro-3-phenoxyphenyl)methanimine, 25** – **68** (126 µL, 0.7 mmol, 1 eq.) was added to **69** (100 mg, 0.7 mmol, 1 eq.) and purged with argon. Anydrous DCE (5 mL) was added and the reaction was heated to reflux. The reaction was monitored *via* TLC and complete after 2 hours. The reaction was cooled to r.t., concentrated under reduced pressure and purified by column chromatography to afford **25** (170 mg, 71%) as an oil. $\mathbf{R}_f$ = 0.2 (Hex/EA 95:5); **^{1}H NMR (400 MHz, $CDCl_3$):** δ ppm 8.24 (s, 1H), 7.69 – 7.62 (m, 2H), 7.41 – 7.33 (m, 2H), 7.32 – 7.26 (m, 1H), 7.20 (d, *J* = 2.0 Hz, 1H), 7.18 – 7.10 (m, 2H), 7.07 – 7.02 (m, 2H), 6.83 (d, *J* = 8.3 Hz, 1H), 2.30 (s, 3H); **^{19}F{^{1}H} NMR (376 MHz, $CDCl_3$):** δ ppm -125.8 (s, 1F); **^{13}C{^{1}H} NMR (101 MHz, $CDCl_3$):** δ ppm 157.6 (d, *J* = 1.5 Hz), 156.9, 156.3 (d, *J* = 255 Hz), 149.0, 144.5 (d, *J* = 13 Hz), 134.0, 133.4 (d, *J* = 3.7 Hz), 131.1, 130.2, 129.9 (2C), 126.6, 125.4 (d, *J* = 7.3 Hz), 123.6, 121.4 (d, *J* = 1.5 Hz), 118.6, 117.6 (2C), 117.4, 17.7; **HRMS (m/z):** $[M+H]^+$ calcd. 340.0899 For $C_{20}H_{16}ClFNO$; found 340.0903.

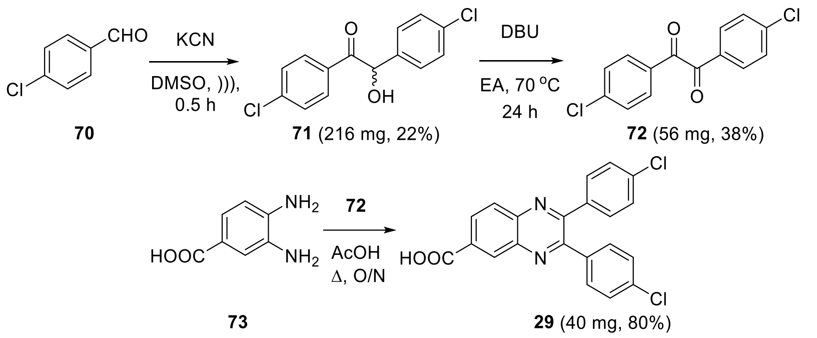

Scheme S10

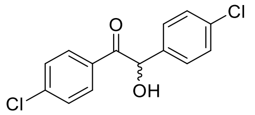

71

1,2-bis(4-chlorophenyl)-2-hydroxyethan-1-one, 71 – **70** (1.0 g, 7.1 mmol, 1 eq.) was dissolved in DMSO (5 mL). KCN (0.88 g, 13.5 mmol, 1.9 eq.) was added in one portion and the reaction was sonicated for 0.5 h at room temperature. H_2O (70 mL) was added, the reaction mixture was extracted with MTBE (3 × 40 mL) and the organic extract was concentrated under reduced pressure. The residue was re-dissolved in MTBE, washed with brine (2 × 30 mL) and dried over $MgSO_4$. The crude product was purified by column chromatography to afford **71** (216 mg, 22%) as a white solid.; **^{1}H NMR (400 MHz, $(CD_3)_2SO$)**: δ ppm 8.01 (d, J = 8.7 Hz, 2H), 7.55 (d, J = 8.7 Hz, 2H), 7.40 (m, 4H), 6.27 (d, J = 5.8 Hz, 1H), 6.06 (d, J = 5.8 Hz, 1H); **^{13}C{^{1}H} NMR (101 MHz, $(CD_3)_2SO$)**: δ ppm 197.9, 138.5, 138.26, 133.3, 132.4, 130.8 (2C), 129.0 (2C), 128.8 (2C), 128.5 (2C), 75.0.

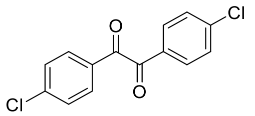

72

1,2-bis(4-chlorophenyl)ethane-1,2-dione, 72 – **71** (145 mg, 0.52 mmol, 1 eq.) was dissolved in ethyl acetate (8 mL) followed by the addition of DBU (20 µL, 0.13 mmol, 0.25 eq.). The reaction was heated to 70 °C and stirred for 24 h. The reaction was cooled and concentrated under reduced pressure. The crude product was purified by column chromatography to afford **72** (56 mg, 38%) as yellow needles. **R_f** = 0.4 (Hex:DCM / 60:40); **^{1}H NMR (400 MHz, $CDCl_3$):** δ ppm 7.92 (m, 4H), 7.50 (m, 4H); **^{13}C{^{1}H} NMR (101 MHz, $CDCl_3$):** δ ppm 192.4, 141.8, 131.3, 131.1, 129.5.

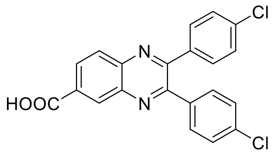

29

2,3-bis(4-chlorophenyl)quinoxaline-6-carboxylic acid, 29 – **72** (36 mg, 0.129 mmol, 1 eq.) and **73** (49 mg, 0.322 mmol, 2.5 eq.) were purged with argon, dissolved in AcOH (2 mL) and heated to reflux O/N. Reaction was monitored by TLC and upon completion was concentrated under reduced pressure and purified by column chromatography to afford **29** (40 mg, 80%) as a white solid. **R_f** = 0.4 (DCM/MeOH/AcOH 94.5:5:0.5); **^{1}H NMR (400 MHz, $(CD_3)_2SO$)**: δ ppm 8.66 (d, *J* = 1.8 Hz, 1H), 8.33 (dd, *J* = 8.8, 1.8 Hz, 1H), 8.24 (d, *J* = 8.8 Hz, 1H), 7.54 (m, 4H), 7.49 (m, 4H); **^{13}C{^{1}H} NMR (101 MHz, $(CD_3)_2SO$)**: δ ppm 166.6, 153.6, 153.0, 142.3, 139.8, 137.1 (2C), 134.2, 134.1, 132.6, 131.7 (2C), 131.6 (2C), 130.6, 129.8, 129.3, 128.3 (4C); **HRMS (*m/z*):** $[M+H]^+$ calcd. 395.0349 For $C_{21}H_{13}Cl_2N_2O_2$; found 395.0355.

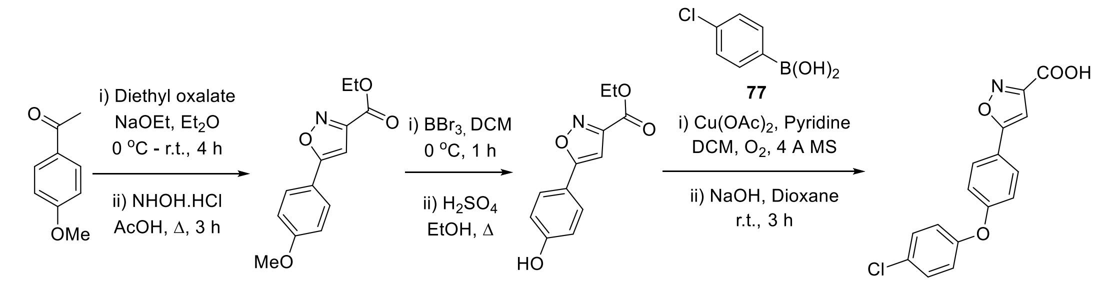

74 **75** (50% / 2 steps) **76** (59% / 2 steps) **30** (41% / 2 steps)

Scheme S11

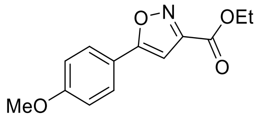

75

ethyl 5-(4-methoxyphenyl)isoxazole-3-carboxylate, 75 – **74** (3 g, 20 mmol, 1 eq.) and sodium ethoxide (1.6 g, 24 mmol, 1.2 eq.) were purged with argon and dissolved in anhydrous Et_2O (40 mL). The reaction was cooled to 0 °C and diethyl oxalate (2.7 mL, 20 mmol, 1 eq.) was added dropwise. After 4 hours the reaction was concentrated under reduced pressure and dried overnight to afford intermediate material **75i** (5.44 g, 20 mmol) which was used without further purification. **75i** (5.44 g, 20 mmol, 1 eq.) and NHOH.HCl (1.67 g, 24 mmol, 1.2 eq.) were dissolved in AcOH (70 mL) and heated to reflux for 3 h. The reaction was cooled and diluted in ice. The precipitate was collected *via* vacuum filtration to afford **75** (2.5 g, 50%) as a beige solid which was used without further purification. **^{1}H NMR (400 MHz, $(CD_3)_2SO$):** δ 7.90 (d, *J* = 8.9 Hz, 2H), 7.34 (s, 1H), 7.10 (d, *J* = 8.9 Hz, 2H), 4.39 (q, *J* = 7.1 Hz, 2H), 3.84 (s, 3H), 1.34 (t, *J* = 7.2 Hz, 3H); **$^{13}C\{^1H\}$ NMR (101 MHz, $(CD_3)_2SO$):** δ ppm 171.2, 161.3, 159.5, 156.7, 127.6 (2C), 118.7, 114.7 (2C), 99.2, 61.8, 55.4, 14.0; **HRMS (m/z):** $[M+H]^+$ calcd. 248.0917 for $C_{13}H_{14}NO_4$; found 248.0920; $[M+Na]^+$ calcd. 270.0737 for $C_{13}H_{13}NNaO_4$; found 270.0740.

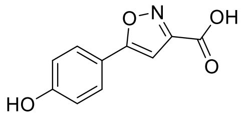

76i

5-(4-hydroxyphenyl)isoxazole-3-carboxylic acid, 76i – **75** (500 mg, 2 mmol, 1 eq.) was purged with argon dissolved in anhydrous DCM (10 mL) and cooled to 0 °C. BBr_3 (4.5 mL, 3 mmol, 1.5 eq.) was added dropwise and the reaction was stirred at 0 °C for 1 h. HPLC-MS analysis suggested substantial deprotection of ester. BBr_3 (9 mL, 6 mmol, 3 eq.) was added and reaction was stirred for 1 h at r.t. The reaction was slowly quenched with ice cold water and the precipitate was collected *via* vacuum filtration to afford **76i** (324 mg, 70%) as a white solid which was used without further purification. **^{1}H NMR (400 MHz, $(CD_3)_2SO$)**: δ 10.17 (br s, 1H), 7.76 (d, *J* = 8.7 Hz, 2H), 7.15 (s, 1H), 6.90 (d, *J* = 8.7 Hz, 2H); **$^{13}C\{^1H\}$ NMR (101 MHz, $(CD_3)_2SO$)**: δ ppm 171.3, 161.1, 159.9, 157.6, 127.2 (2C), 117.4, 116.1 (2C),

98.7; **HRMS (m/z):** [M+H]+ calcd. 206.0448 for $C_{10}H_8NO_4$; found 206.0446; [M+Na]+ calcd. 228.0267 for $C_{10}H_7NNaO_4$; found 228.0268.

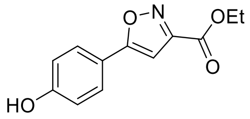

76

ethyl 5-(4-hydroxyphenyl)isoxazole-3-carboxylate, 76 – **76i** (250 mg, 1.2 mmol, 1 eq.) was dissolved in EtOH (15 mL) and acidified with the slow addition of H_2SO_4 (0.5 mL, 9.3 mmol, 8 eq.). The reaction was heated to reflux O/N and then concentrated under reduced pressure. The crude residue was taken up in sat. $NaHCO_3$ and the precipitate collected *via* vacuum filtration to afford **76** (233 mg, 84%) as a brown solid which was used without further purification. **^{1}H NMR (400 MHz, $(CD_3)_2SO$)**: δ ppm 10.17 (s, 1H) 7.80 – 7.75 (m, 2H) 7.23 (s, 1H) 6.93 – 6.88 (m, 2H) 4.38 (q, *J* = 7.2 Hz, 2H) 1.33 (t, *J* = 7.2 Hz, 3H); **^{13}C{^{1}H} NMR (101 MHz, $(CD_3)_2SO$):** δ ppm 171.6, 160.0, 159.6, 156.7, 127.7 (2C), 117.2, 116.0 (2C), 98.5, 61.8, 14.0; **HRMS (m/z)**: [M+Na]+ calcd. 256.0580 For $C_{12}H_{11}NNaO_4$; found 256.0579.

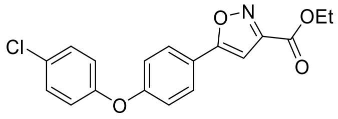

30i

ethyl 5-(4-(4-chlorophenoxy)phenyl)isoxazole-3-carboxylate, 30i – **76** (50 mg, 0.21 mmol, 1 eq.), **77** (100 mg, 0.64 mmol, 3 eq.) and $Cu(OAc)_2$ (38 mg, 0.21 mmol, 1 eq.) were dissolved in anhydrous DCM (5 mL) over 4Å MS. Pyridine (85 µL, 1.05 mmol, 5 eq.) was added and the reaction was stirred at room temperature overnight. **77** (200 mg, 1.28 mmol, 6 eq.) was further added and oxygen was bubbled through the reaction O/N. HPLC-MS confirmed consumption of **76**. The reaction mixture was filtered over a pad of silica with ethyl acetate (200 mL), concentrated and purified by column chromatography to afford **30i** (44 mg, 62%) as a white solid. **R_f** = 0.3 (Hexane/DCM 1:1); **^{1}H NMR (400 MHz, $CDCl_3$):** δ ppm 7.77 (d, *J* = 8.9 Hz, 2H) 7.35 (d, *J* = 9.0 Hz, 2H) 7.07 (d, *J* = 8.9 Hz, 2H) 7.01 (d, *J* = 9.0 Hz, 2H) 6.85 (s, 1H) 4.47 (q, *J* = 7.1 Hz, 2H) 1.44 (t, *J* = 7.2 Hz, 3H); **^{13}C{^{1}H} NMR (101 MHz, $CDCl_3$):** δ ppm 171.1 (s, 1C) 160.0 (s, 1C) 159.4 (s, 1C) 157.0 (s, 1C) 154.5 (s, 1C) 130.1 (s, 1C) 129.5 (s, 1C) 127.8 (s, 1C) 121.8 (s, 1C) 121.0 (s, 1C) 118.6 (s, 1C) 99.3 (s, 1C) 62.2 (s, 1C) 14.2 (s, 1C); **HRMS (m/z):** [M+H]+ calcd. 344.0684 for $C_{18}H_{15}ClNO_4$; found 344.0683; [M+Na]+ calcd. 366.0504 for $C_{18}H_{14}ClNNaO_4$; found 366.0506.

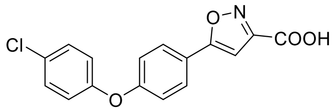

30

5-(4-(4-chlorophenoxy)phenyl)isoxazole-3-carboxylic acid, 30 – **30i** (50 mg, 0.145 mmol, 1 eq.) was dissolved in dioxane (0.58 mL) under an inert atmosphere. Aq. NaOH (1M, 0.58 mL, 0.58 mmol, 4 eq.) was added and the reaction was stirred at r.t., for 3 hours and monitored by TLC. Upon completion, the reaction was concentrated, suspended in H_2O (20 mL), acidified with 2M HCl (4 mL) and extracted with EA (4 x 40 mL). The combined organic extracts were washed with sat. brine (40 mL), dried with $MgSO_4$, filtered and concentrated under reduced pressure. The crude material was purified by column chromatography to afford **30** (30 mg, 66%) as a white solid. **R_f** = 0.2 (DCM/MeOH/AcOH 90:9:1); **^{1}H NMR (400 MHz, $(CD_3)_2SO$)**: δ ppm 8.00 – 7.91 (m, 2H), 7.53 – 7.44 (m, 2H), 7.30 (s, 1H), 7.20 – 7.08 (m, 4H); **^{13}C{^{1}H} NMR (101 MHz, $(CD_3)_2SO$):** δ ppm 169.9, 161.0, 158.5, 154.5, 130.3, 130.1 (2C), 128.2, 128.0 (2C), 121.3 (2C), 118.7 (2C), 117.5, 100.3; **HRMS (m/z):** $[M+H]^+$ calcd. 316.0371 for $C_{16}H_{11}ClNO_4$; found 316.0367; $[M+Na]^+$ calcd. 338.0191 for $C_{16}H_{10}ClNNaO_4$; found 338.0189.

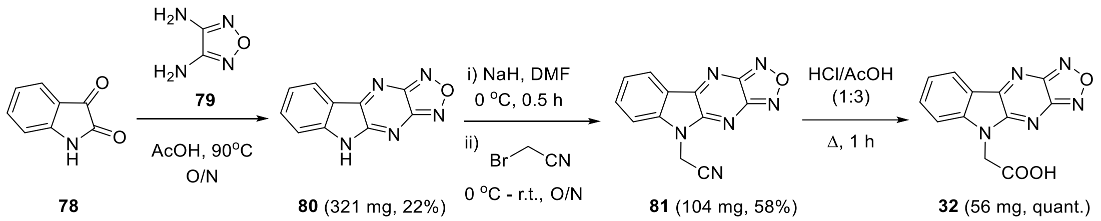

Scheme S12

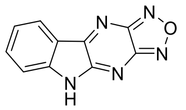

80

5*H*-[1,2,5]oxadiazolo[3',4':5,6]pyrazino[2,3-*b*]indole, 80 – **78** (1.04 g, 7.1 mmol, 1 eq.) and **79** (710 mg, 7.1 mmol, 1 eq.) were evacuated and filled with nitrogen thrice. Anhydrous AcOH (20 mL, 0.36 M) was added. The reaction was heated to 90 °C and stirred O/N. The reaction mixture was cooled to r.t., poured onto ice-cold water (150 mL). The resulting red precipitate was collected *via* vacuum filtration, washed with cold water and dried under reduced pressure. The crude material was purified by column chromatography to afford **80** (321 mg, 22%) as a red solid. **R_f** = 0.3 (Hexane/Ethyl acetate 50:50); **^{1}H NMR (400 MHz, $(CD_3)_2SO$)**: δ ppm 13.36 – 11.09 (br s, 1H) 8.23 (ddd, *J* = 7.8, 1.3, 0.7 Hz, 1H) 7.78 (ddd, *J* = 8.1, 7.4, 1.3 Hz, 1H) 7.42 (dt, *J* = 8.1, 0.8 Hz, 1H) 7.35 (td, *J* = 7.6, 0.9 Hz, 1H); **^{13}C{^{1}H} NMR (101 MHz, $(CD_3)_2SO$)**: δ ppm 153.4, 152.0, 151.9, 151.3, 148.2, 135.7, 124.7, 122.4, 118.2, 112.7; **HRMS (m/z)**: $[M+H]^+$ calcd. 212.0567 for $C_{10}H_6N_5O$; found 212.0566.

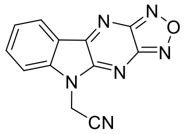

81

2-(5*H*-[1,2,5]oxadiazolo[3',4':5,6]pyrazino[2,3-*b*]indol-5-yl)acetonitrile, 81 – **80** (150 mg, 0.71 mmol, 1 eq.) was evacuated and filled with nitrogen thrice and dissolved in AHS DMF (10 mL). NaH (34 mg, 0.85 mmol, 1.2 eq.) was evacuated and filled with nitrogen thrice, dissolved in AHS DMF (10 mL) and cooled to 0 °C. The solution of **80** was added dropwise to the solution of NaH. The reaction was stirred at 0 °C for 0.5 h followed by the addition of bromoacetonitrile (59 µL, 0.85 mmol, 1.2 eq.). The reaction was warmed to r.t., and stirred O/N. The reaction was poured onto sat. brine (250 mL) and further diluted with H_2O (50 mL). The aqueous phase was extracted with Et_2O (5 x 150 mL), dried over $MgSO_4$ and concentrated under reduced pressure. The crude material was purified by column chromatography to afford **76** (104 mg, 58%) as a yellow solid. **R_f** = 0.3 (Hexane/Ethyl acetate 50:50); **^{1}H NMR (400 MHz, $(CD_3)_2SO$)**: δ ppm 8.36 – 8.32 (app. m, 1H) 7.95 (ddd, *J* = 8.3, 7.3, 1.3 Hz, 1H) 7.84 (d, *J* = 8.2 Hz, 1H) 7.51 (td, *J* = 7.5, 0.8 Hz, 1H) 5.63 (s, 2H); **^{13}C{^{1}H} NMR (101 MHz, $(CD_3)_2SO$)**: δ ppm

152.8, 151.8, 151.4, 151.0, 146.8, 135.8, 124.9, 123.9, 118.5, 115.1, 111.4, 29.5; **HRMS (m/z)**: $[M+H]^+$ calcd. 251.0676 for $C_{12}H_7N_6O$; found 251.0675.

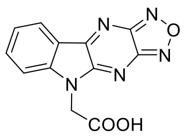

32

2-(5H-[1,2,5]oxadiazolo[3',4':5,6]pyrazino[2,3-b]indol-5-yl)acetic acid, 32 – **81** (45 mg, 018 mmol, 1 eq.) was purged with N_2. A solution of conc. HCl/AcOH (1:3, 30 mL) was added and the reaction was heated to 120 °C, monitored *via* HPLC-MS and complete within 1 h. The reaction was cooled to r.t., diluted with H_2O (250 mL) and adjusted to pH 5 with 2M NaOH. The aqueous phase was extracted with ethyl acetate (3 x 150 mL). The combined organic phases were washed with sat. acidic brine (50 mL), dried over $MgSO_4$, filtered and concentrated under reduced pressure. The crude material was purified by column chromatography to afford **32** (48 mg, quant.) as an orange solid. **R_f** = 0.2 (DCM/MeOH/AcOH 90:9:1); **^{1}H NMR (400 MHz, $(CD_3)_2SO$)**: δ ppm 8.31 (d, *J* = 7.7 Hz, 1H), 7.86 (t, *J* = 7.5 Hz, 1H), 7.70 (d, *J* = 8.2 Hz, 1H), 7.44 (t, *J* = 7.6 Hz, 1H), 5.10 (s, 2H); **$^{13}C\{^1H\}$ NMR (101 MHz, $(CD_3)_2SO$)**: δ ppm 168.6, 152.6, 151.6, 151.5, 151.0, 148.5, 135.8, 124.6, 123.22, 117.8, 111.7, 42.9; **HRMS (m/z)**: $[M+H]^+$ calcd. 270.0622 for $C_{12}H_8N_5O_3$; found 270.0620.

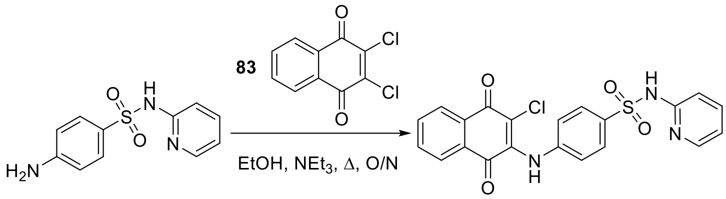

82 **33** (130 mg, 36%)

Scheme S13

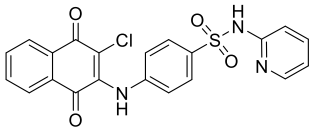

33

4-((3-chloro-1,4-dioxo-1,4-dihydronaphthalen-2-yl)amino)-N-(pyridin-2-yl)benzenesulfonamide, 33 – **83** (377 mg, 1.6 mmol, 2 eq.) and **82** (204 mg, 0.8 mmol, 1 eq.) were purged with argon and dissolved in EtOH (4 mL). NEt_3 (111.5 µL, 0.8 mmol, 1 eq.) was added and the reaction was refluxed O/N. The reaction was cooled to r.t., and the resultant precipitate was isolated *via* vacuum filtration, washed with cold EtOH and purified *via* column chromatography to afford **33** (130 mg, 36%) as a red solid. $\mathbf{R_{\mathit{f}}}$ = 0.3 (DCM/MeOH 96:4); **^{1}H NMR (400 MHz, $(CD_3)_2SO$)**; δ ppm 12.05 – 11.45 (br s, 1H), 9.49 (s, 1H), 8.06 – 7.99 (m, 3H), 7.87 (td, *J* = 7.4, 1.6 Hz, 1H), 7.82 (td, *J* = 7.4, 1.5 Hz, 1H), 7.79 – 7.75 (m, 2H), 7.71 (ddd, *J* = 8.7, 7.1, 1.9 Hz, 1H), 7.21 – 7.13 (m, 3H), 6.92 – 6.84 (m, 1H); **^{13}C{^{1}H} NMR (101 MHz, $(CD_3)_2SO$)**; δ ppm 179.9, 177.0, 152.9, 142.7, 142.7, 140.1, 135.8, 134.7, 133.5, 131.7, 130.5, 128.9, 126.8 (2C), 126.6, 126.2, 122.2 (2C), 118.3, 113.6, 112.4; **HRMS (m/z)**: $[M+H]^+$ calcd. 440.0466 for $C_{21}H_{15}ClN_3O_4S$; found 440.0458; $[M+Na]^+$ calcd. 462.0286 for $C_{21}H_{14}ClN_3NaO_4S$; found 462.0284.

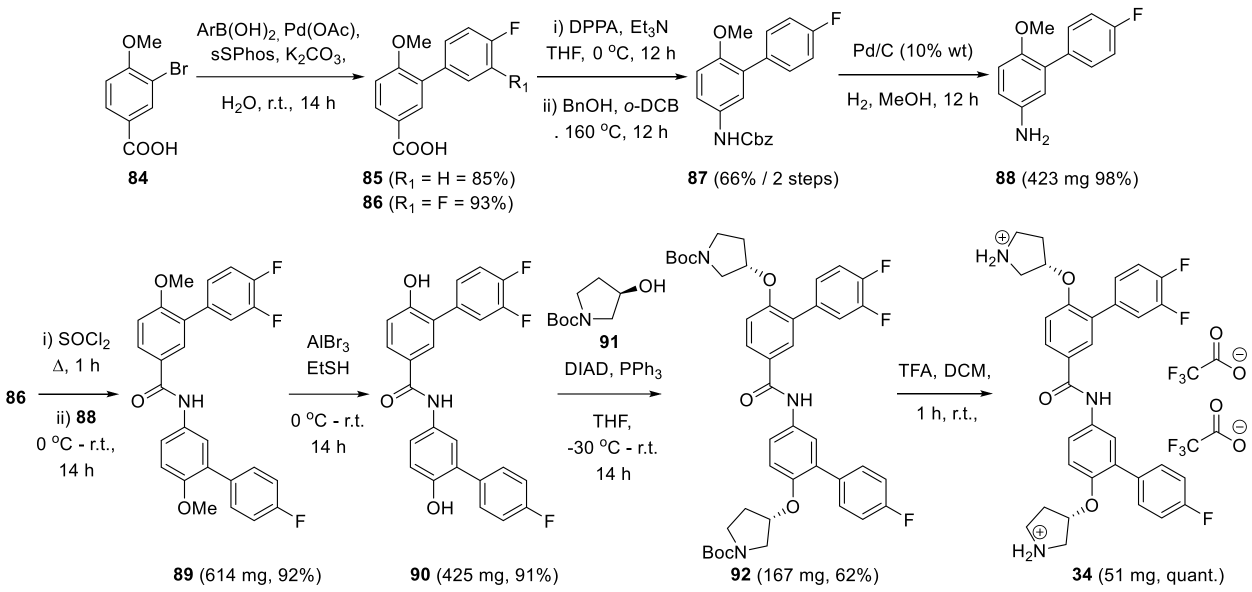

Scheme S14

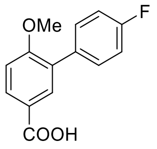

85

4'-fluoro-6-methoxy-[1,1'-biphenyl]-3-carboxylic acid, 85 – **84** (1.00 g, 4.33 mmol, 1.0 eq.), 4-fluorobenzenboronic acid (726 mg, 5.19 mmol, 1.2 eq.), Pd(OAc)$_2$ (38 mg, 0.17 mmol, 0.04 eq.), sSPhos (177 mg, 0.346 mmol, 0.08 eq.) and K_2CO_3 (1.80 g, 13 mmol, 3 eq.) were sealed in a microwave vial with magnetic stirrer, evacuated and filled with N_2 thrice. Degassed H_2O (15 mL, 0.29 M) was added and the reaction was stirred at r.t., O/N. The reaction was acidified with 2M HCl (10 mL) and the resulting precipitate was collected by vacuum filtration. The aq. filtrate was extracted with EA (3 x 30 mL) and combined with the precipitate. The combined organics were washed with sat. brine (30 mL), dried with $MgSO_4$ and filtered through a pad of celite, washing with EA (100 mL). The organic extract was concentrated under reduced pressure and purified by column chromatography to afford **85** (901 mg, 85%) as a white solid. **R_f** = 0.2 (DCM/EA/AcOH 95:5:1); **^{1}H NMR (400 MHz, (CD$_3$)$_2$SO)**: δ ppm 12.74 (br s, 1H), 7.95 (dd, *J* = 8.6, 2.2 Hz, 1H), 7.81 (d, *J* = 2.2 Hz, 1H), 7.56 – 7.49 (m, 2H), 7.29 – 7.19 (m, 3H), 3.85 (s, 3H); **^{19}F{^{1}H} NMR (376 MHz, (CD$_3$)$_2$SO):** δ ppm -114.9 (s, 1F); **^{13}C{^{1}H} NMR (101 MHz, (CD$_3$)$_2$SO):** δ ppm 166.9, 161.5 (d, *J* = 244 Hz) 159.6, 133.6 (d, *J* = 2.9 Hz) 131.5, 131.2 (d, *J* = 8.1 Hz, 2C) 130.8, 128.6, 123.1, 115.0 (d, *J* = 21 Hz, 2C) 111.5, 55.9; **HRMS (m/z):** [M-H]$^-$ calcd. 245.0619 for $C_{14}H_{10}FO_3$; found 245.0626.

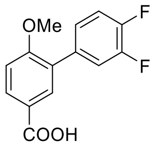

86

3',4'-difluoro-6-methoxy-[1,1'-biphenyl]-3-carboxylic acid, 86 – **84** (1.00 g, 4.33 mmol, 1.0 eq.), 3,4-difluorobenzenboronic acid (820 mg, 5.19 mmol, 1.2 eq.), $Pd(OAc)_2$ (38 mg, 0.17 mmol, 0.04 eq.), sSPhos (177 mg, 0.346 mmol, 0.08 eq.) and K_2CO_3 (1.80 g, 13 mmol, 3 eq.) were sealed in a microwave vial with magnetic stirrer, evacuated and filled with N_2 thrice. Degassed H_2O (15 mL, 0.29 M) was added and the reaction was stirred at r.t., O/N. The reaction was acidified with 2M HCl (10 mL) and the resulting precipitate was collected by vacuum filtration. The aq. filtrate was extracted with EA (3 x 30 mL) and combined with the precipitate. The combined organics were washed with sat. brine (30 mL), dried with $MgSO_4$ and filtered through a pad of celite, washing with EA (100 mL). The organic extract was concentrated under reduced pressure and purified by column chromatography to afford **86** (1.06 g, 93%) as a white solid. **R*f*** = 0.2 (DCM/EA/AcOH 95:5:1); **^{1}H NMR (400 MHz, $(CD_3)_2SO$)**: δ ppm 12.76 (br s, 1H), 7.97 (dd, *J* = 8.7, 2.2 Hz, 1H), 7.84 (d, *J* = 2.2 Hz, 1H), 7.57 (ddd, *J* = 12.1, 8.0, 2.1 Hz, 1H), 7.47 (dt, *J* = 10.8, 8.6 Hz, 1H), 7.36 – 7.30 (m, 1H), 7.23 (d, *J* = 8.8 Hz, 1H), 3.86 (s, 3H); **^{19}F{^{1}H} NMR (376 MHz, $(CD_3)_2SO$):** δ ppm -138.9 (d, *J* = 22.5 Hz, 1F) -140.3 (d, *J* = 22.5 Hz, 1F); **^{13}C{^{1}H} NMR (101 MHz, $(CD_3)_2SO$):** δ ppm 166.8, 159.5, 149.0 (dd, *J* = 245.0, 13 Hz), 148.8 (dd, *J* = 246, 13 Hz), 134.6 (dd, *J* = 6.6, 4.4 Hz), 131.5, 131.2, 127.4, 126.1 (dd, *J* = 6.6, 2.9 Hz), 123.2, 118.3 (d, *J* = 18 Hz), 117.2 (d, *J* = 17 Hz), 111.6, 56.0; **HRMS (m/z):** $[M+H]^+$ calcd. 265.0671 for $C_{14}H_{11}F_2O_3$; found 265.0673.

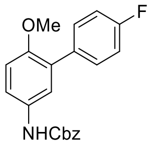

87

benzyl (4'-fluoro-6-methoxy-[1,1'-biphenyl]-3-yl)carbamate, 87 – **85** (832 mg, 3.38 mmol, 1 eq.) was evacuated and filled with N_2 thrice, dissolved in anhydrous THF (17 mL, 0.2 M) and cooled to 0 °C. NEt_3 (564 µL, 4.06 mmol, 1.2 eq.) was added, followed diphenylphosphoryl azide (728 µL, 3.38 mmol, 1 eq.). The reaction was stirred vigorously and warmed to r.t., O/N. The reaction was diluted with sat. $NaHCO_3$ (100 mL), H_2O (100 mL) and triturated for 0.5 h. The resultant precipitate was collected *via* vacuum filtration to afford intermediate acyl azide (727 mg, 80%) as a white solid which was used without further purification. Acyl azide (700 mg, 2.58 mmol, 1 eq.) was evacuated and filled with N_2 thrice, dissolved in anhydrous *ortho*-dichlorobenzene (55 mL, 0.05 M) and heated until gas evolution was observed (155 °C). Gas evolution ceased after 20 mins. The reaction was then cooled to 100 °C and anhydrous benzyl alcohol (533 µL, 5.16 mmol, 2 eq.) was added in one portion. The reaction was

left to stir at 100 °C overnight. The reaction was cooled, concentrated under reduced pressure and purified by column chromatography to afford **87** (744 mg, 82%) as a white solid. $\mathbf{R_f}$ = 0.25 (Hexane/EA 80:20); **^{1}H NMR (400 MHz, $(CD_3)_2SO$)**: δ ppm 9.64 (br s, 1H) 7.50 – 7.31 (m, 9H) 7.27 – 7.20 (m, 2H) 7.05 (d, *J* = 8.7 Hz, 1H) 5.14 (s, 2H) 3.71 (s, 3H); **^{19}F{^{1}H} NMR (376 MHz, $(CD_3)_2SO$):** δ ppm -115.4 (s, 1F); **^{13}C{^{1}H} NMR (101 MHz, $(CD_3)_2SO$):** δ ppm 161.8 (d, *J* = 244 Hz), 154.0, 152.1, 137.2, 134.8 (d, *J* = 3.7 Hz), 132.8, 131.5 (d, *J* = 8.1 Hz, 2C), 129.2, 128.9 (2C), 128.5 (2C), 128.5, 121.2, 119.3, 115.3 (d, *J* = 21 Hz, 2C), 112.8, 66.1, 56.3; **HRMS (m/z):** $[M+H]^+$ calcd. 352.1343 for $C_{21}H_{19}FNO_3$; found 352.1344; $[M+Na]^+$ calcd. 374.1163 for $C_{21}H_{18}FNNaO_3$; found 374.1164.

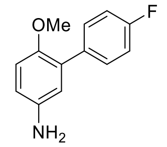

88

4'-fluoro-6-methoxy-[1,1'-biphenyl]-3-amine, 88 – **87** (700 mg, 1.99 mmol, 1 eq.) and Pd/C (211 mg, 0.2 mmol, 0.1 eq.) were evacuated and filled with N_2 thrice and dissolved in anhydrous methanol (20 mL, 0.1 M). A balloon of H_2 was bubbled through the solution for 10 minutes with vigorous stirring. The reaction was then left under an atmosphere of H_2 O/N. The reaction was concentrated, diluted in EA (50 mL), filtered through a pad of celite which was washed with extra EA (100 mL). The reaction mixture was concentrated under reduced pressure and purified by column chromatography to afford **88** (423 mg, 98%) as an amber oil. $\mathbf{R_f}$ = 0.3 (Hexane/EA 1:1); **^{1}H NMR (500 MHz, $(CD_3)_2SO$)** δ ppm 7.47 – 7.42 (m, 2H) 7.19 (tt, *J* = 9.0, 2.3 Hz, 2H) 6.82 (dt, *J* = 8.9, 1.3 Hz, 1H) 6.56 (dd, *J* = 8.8, 2.9 Hz, 2H) 4.72 (s, 2H) 3.60 (s, 3H); **^{19}F{^{1}H} NMR (471 MHz, $(CD_3)_2SO$):** δ ppm -116.2 (s, 1F); **^{13}C{^{1}H} NMR (126 MHz, $(CD_3)_2SO$):** δ ppm 161.1 (d, *J* = 243 Hz), 147.5, 142.7, 135.1 (d, *J* = 3.1 Hz), 130.9 (d, *J* = 8.1 Hz, 2C), 129.3, 116.3, 114.6 (d, *J* = 21 Hz, 2C), 114.0, 113.7, 56.2; **HRMS (m/z):** $[M+H]^+$ calcd. 218.0976 for $C_{13}H_{13}FNO$; found 218.0977.

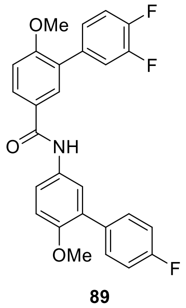

89

3',4'-difluoro-*N*-(4'-fluoro-6-methoxy-[1,1'-biphenyl]-3-yl)-6-methoxy-[1,1'-biphenyl]-3-carboxamide, 89 – **86** (380 mg, 1.44 mmol, 1 eq.) was evacuated and filled with N_2 thrice, suspended

in $SOCl_2$ (5 mL, 69 mmol, 48 eq.) and heated to reflux for 1 hour. The reaction was cooled and concentrated under reduced pressure to afford a white powder. The crude powder was evacuated and filled with N_2 thrice, dissolved in anhydrous DCM (3 mL, 0.48 M) and cooled to 0 °C. **88** (312 mg, 1.44 mmol, 1 eq.) was evacuated and filled with N_2 thrice, dissolved in anhydrous DCM (3 mL, 0.48 M) and added dropwise to the chilled solution containing **86**. A precipitate was instantly formed, and the reaction was stirred overnight. The reaction was diluted in EA (30 mL) and washed with sat. $NaHCO_3$ (30 mL). The aq. phase was further extracted with EA (2 x 30 mL). The combined organics were washed with sat. brine (30 mL), dried over $MgSO_4$, filtered and concentrated under reduced pressure to afford crude **89** (614 mg, 92%) as a white solid which was used without further purification. **R*f*** = 0.3 (Hexane/EA 3:2); **^{1}H NMR (400 MHz, $(CD_3)_2SO$)**: δ ppm 10.08 (s, 1H) 8.02 (dd, *J* = 8.7, 2.3 Hz, 1H) 7.98 (d, *J* = 2.3 Hz, 1H) 7.76 (dd, *J* = 8.8, 2.7 Hz, 1H) 7.70 (d, *J* = 2.7 Hz, 1H) 7.64 (ddd, *J* = 12.1, 8.0, 2.1 Hz, 1H) 7.55 – 7.49 (m, 3H) 7.45 – 7.39 (m, 1H) 7.29 – 7.22 (m, 3H) 7.11 (d, *J* = 8.9 Hz, 1H) 3.87 (s, 3H) 3.76 (s, 3H); **^{19}F{^{1}H} NMR (376 MHz, $(CD_3)_2SO$)**: δ ppm -115.4 (s, 1F) -139.0 (d, *J* = 22.5 Hz, 1F) -140.3 (d, *J* = 22.5 Hz, 1F); **^{13}C{^{1}H} NMR (101 MHz, $(CD_3)_2SO$)**: δ ppm 164.2, 161.3 (d, *J* = 244 Hz), 158.5, 152.2, 150.2 (dd, *J* = 245, 12.5 Hz), 148.8 (dd, *J* = 247, 12.5 Hz), 134.9 (dd, *J* = 6.6, 3.7 Hz), 134.3 (d, *J* = 2.9 Hz), 132.6, 131.1 (d, *J* = 8.1 Hz, 2C), 129.7, 129.6, 128.5, 127.1, 127.0, 126.3 (dd, *J* = 6.2, 3.3 Hz), 122.9, 120.9, 118.4 (d, *J* = 18 Hz), 117.1 (d, *J* = 17 Hz), 114.9 (d, *J* = 21 Hz, 2C), 111.9, 111.6, 56.0, 55.7; **HRMS (m/z):** $[M+H]^+$ calcd. 464.1468 for $C_{27}H_{21}F_3NO_3$; found 464.1458.

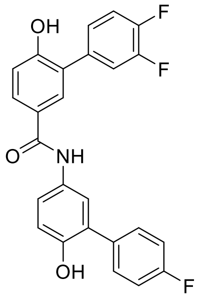

90

3',4'-difluoro-N-(4'-fluoro-6-hydroxy-[1,1'-biphenyl]-3-yl)-6-hydroxy-[1,1'-biphenyl]-3-carboxamide, 90 – **89** (500 mg, 1.08 mmol, 1 eq.) was evacuated and filled with N_2 thrice, suspended in EtSH (5 mL) and cooled to 0 °C. $AlBr_3$ (1.86 g, 6.97 mmol, 6.46 eq.) was evacuated and filled with N_2 for thrice, dissolved in EtSH (20 mL) and added to the cooled solution containing **89** (0.04 M). The reaction was stirred vigorously and warmed to r.t., O/N. The reaction was stopped by the slow addition of 0.5 M HCl (50 mL) and extracted with EA (4 x 40 mL). The combined organics were washed with sat. brine (50 mL), dried over $MgSO_4$, filtered and concentrated under reduced pressure. The crude solid was purified by column chromatography to afford **90** (425 mg, 91%) as a white solid. **R*f*** = 0.3 (Hexane/EA/AcOH 45:54:1); **^{1}H NMR (500 MHz, $(CD_3)_2SO$)**: δ ppm 10.92 – 10.06 (br s, 1H) 9.92 (s, 1H)

9.80 – 9.11 (br s, 1H) 7.98 (d, *J* = 2.3 Hz, 1H) 7.85 (dd, *J* = 8.5, 2.3 Hz, 1H) 7.74 – 7.67 (m, 1H) 7.65 (d, *J* = 2.6 Hz, 1H) 7.62 – 7.55 (m, 3H) 7.54 – 7.46 (m, 2H) 7.27 – 7.21 (m, 2H) 7.07 (d, *J* = 8.5 Hz, 1H) 6.92 (d, *J* = 8.7 Hz, 1H); **$^{19}F\{^1H\}$ NMR (376 MHz, $(CD_3)_2SO$):** δ ppm -115.8 (s, 1F) -139.1 (d, *J* = 22.5 Hz, 1F) -140.8 (d, *J* = 22.5 Hz, 1F); **$^{13}C\{^1H\}$ NMR (126 MHz, $(CD_3)_2SO$):** δ ppm 164.4, 161.1 (d, *J* = 243 Hz), 157.2, 150.4, 149.0 (dd, *J* = 244, 13 Hz), 148.5 (dd, *J* = 246, 13 Hz), 135.3 (dd, *J* = 6.4, 3.8 Hz), 134.8 (d, *J* = 3.1 Hz), 131.4, 130.9 (d, *J* = 7.9 Hz, 2C) 129.8, 129.2, 126.3, 126.1, 126.1 (dd, *J* = 6.2, 3.3 Hz), 124.9, 122.9, 121.3, 118.1 (d, *J* = 17 Hz), 117.1 (d, *J* = 17 Hz), 116.0, 115.9, 114.8 (d, *J* = 21 Hz, 2C); **HRMS (m/z):** $[M+H]^+$ calcd. 436.1155 for $C_{25}H_{17}F_3NO_3$; found 436.1159.

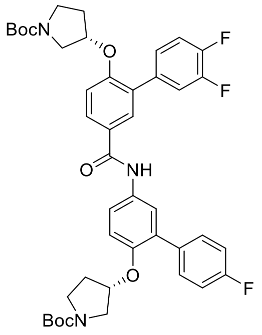

92

^{tert}butyl (S)-3-((5-(6-(((S)-1-(^{tert}butoxycarbonyl)pyrrolidin-3-yl)oxy)-3',4'-difluoro-[1,1'-biphenyl]-3-carboxamido)-4'-fluoro-[1,1'-biphenyl]-2-yl)oxy)pyrrolidine-1-carboxylate, 92 – **90** (150 mg, 0.35 mmol, 1 eq.), **91** (144 mg, 0.77 mmol, 2.2 eq.) and triphenylphosphine (230 mg, 0.88 mmol, 2.5 eq.) were evacuated and filled with N_2 thrice, cooled to -30 °C and dissolved in anhydrous THF (3.5 mL, 0.1 M). DIAD (172 µL, 0.88 mmol, 2.5 eq.) was added and the reaction was warmed to r.t., and stirred O/N. The reaction was diluted in EA (30 mL), anhydrous $ZnCl_2$ (238 mg, 1.75 mmol, 5 eq.) was added and the reaction was stirred for a further 1 h. The precipitate was removed by vacuum filtration and the filtrate was washed with sat. $NaHCO_3$ (2 x 30 mL). The aqueous phase was further extracted with EA (2 x 40 mL). The combined organics were washed with sat. brine (30 mL), dried with $MgSO_4$, filtered and concentrated under reduced pressure. The crude residue was purified by column chromatography to afford **92** (167 mg, 62%) as a white solid. **R_f** = 0.15 (Hexane/Ethyl acetate 35:65); **^1H NMR (400 MHz, $(CD_3)_2SO$)**: δ ppm 10.12 (s, 1H), 8.03 – 7.97 (m, 2H), 7.79 – 7.73 (m, 2H), 7.65 – 7.43 (m, 4H), 7.41 – 7.30 (m, 2H), 7.28 – 7.12 (m, 3H), 5.18 (br s, 1H), 4.96 (br s, 1H), 3.58 – 3.35 (m, 5H), 3.31 – 3.01 (m, 3H), 2.23 – 2.01 (m, 3H), 1.97 (m, 1H), 1.38 (s, 9H), 1.36 (s, 9H); **$^{19}F\{^1H\}$ NMR (376 MHz, $(CD_3)_2SO$):** δ ppm -115.3 (br d, *J* = 15.6 Hz, 1F) -138.9 (br dd, *J* = 31.2, 22.5 Hz, 1F) -140.26 (br dd, *J* = 22.5, 5.2 Hz, 1F);**$^{13}C\{^1H\}$ NMR (101 MHz, $(CD_3)_2SO$)**: δ ppm 164.7, 161.8 (d, *J* = 244 Hz), 156.4 (d, *J* = 12 Hz), 154.1, 150.0, 149.9, 149.4 (dd, *J* = 246, 14 Hz), 149.2 (dd, *J* = 246, 13 Hz), 135.2 (dd, *J* = 6.6, 3.7 Hz), 134.6 (d, *J* = 2.9 Hz), 133.7 (d, *J* = 5.1 Hz), 131.40 (d, *J* = 8.1 Hz, 2C), 130.7, 130.5, 129.9, 128.6 (d, *J* = 18 Hz),

128.1 (d, *J* = 10 Hz), 126.6 (dd, *J* = 5.9, 2.9 Hz), 123.4, 121.3, 118.7 (d, *J* = 17 Hz), 117.5 (d, *J* = 17 Hz), 115.8 (d, *J* = 26 Hz), 115.2 (d, *J* = 23 Hz, 2C), 114.4 (d, *J* = 24 Hz), 78.94 (2C), 78.86, 78.8, 77.6, 76.8, 76.7, 55.4 (2C), 51.8, 51.6, 51.3, 44.3, 44.2, 31.3, 30.5, 30.4, 28.6 (3C), 28.5 (3C); **HRMS (m/z):** $[M+H]^+$ calcd. 796.3180 for $C_{43}H_{46}F_3N_3NaO_7$; found 796.3165.

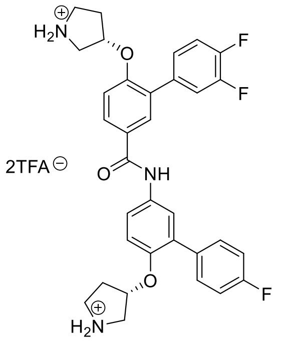

34

(S)-3-((3',4'-difluoro-5-((4'-fluoro-6-(((S)-pyrrolidin-1-ium-3-yl)oxy)-[1,1'-biphenyl]-3-yl)carbamoyl)-[1,1'-biphenyl]-2-yl)oxy)pyrrolidin-1-ium trifluoroacetate, 34 – **92** (50 mg, 0.064 mmol, 1 eq.) was dissolved in anhydrous DCM (500 µL) followed by the addition of TFA (277 µL, 3.62 mmol, 60 eq.). The reaction was monitored by TLC and stopped after 1 hour. The reaction was concentrated under reduced pressure to afford **34** (51 mg, quant) as an off white solid. **34** was further purified by HPLC; **^{1}H NMR ((CD$_3$)$_2$SO, 400 MHz)**: δ ppm 10.2 (s, 1H), 9.28 – 8.81 (m, 4H), 8.04 – 7.99 (m, 2H), 7.80 – 7.75 (m, 2H), 7.71 (ddd, *J* = 12.1, 8.0, 2.1 Hz, 1H), 7.61 – 7.54 (m, 2H), 7.53 – 7.42 (m, 2H), 7.32 – 7.22 (m, 3H), 7.15 (d, *J* = 8.9 Hz, 1 H), 5.26 (br s, 1H), 5.03 (br t, *J* = 4.6 Hz, 1H), 3.57 (dd, *J* = 13.3, 5.0 Hz, 1H), 3.48 (dd, *J* = 13.1, 5.0 Hz, 1 H), 3.42 – 3.33 (m, 4H), 3.21 – 3.04 (m, 2H), 2.35 – 2.19 (m, 1H), 2.18 – 1.98 (m, 3H); **^{19}F{^{1}H} NMR (471 MHz, (CD$_3$)$_2$SO):** δ ppm -73.5 (s, 6F), -115.4 (s, 1F) -139.02 (d, *J* = 22.9 Hz, 1 F), -140.5 (d, *J* = 22.9 Hz, 1F); **^{13}C{^{1}H} NMR (126 MHz, (CD$_3$)$_2$SO):** δ ppm 164.7, 161.9 (d, *J* = 244 Hz), 158.4 (q, *J* = 31 Hz), 156.2, 149.8, 149.5 (dd, *J* = 244.5, 13 Hz), 149.3 (dd, *J* = 246, 13 Hz), 135.1 (dd, *J* = 6.4, 3.8 Hz), 134.5 (d, *J* = 3.1 Hz), 133.9, 131.6 (d, *J* = 8.1 Hz, 2C), 130.7, 130.2, 129.9, 128.3, 128.1, 126.9 (dd, *J* = 5.8, 3.2 Hz), 123.7, 121.3, 119.00 (d, *J* = 18 Hz), 118.99, 117.6 (d, *J* = 17 Hz), 115.5 (d, *J* = 21 Hz, 2C), 115.2, 113.7, 76.9, 76.7, 50.5 (2C), 44.6, 44.4, 31.4, 31.3; **HRMS (m/z):** $[M-H]^-$ calcd. 574.2312 for $C_{33}H_{31}F_3N_3O_3$; found 574.2306.

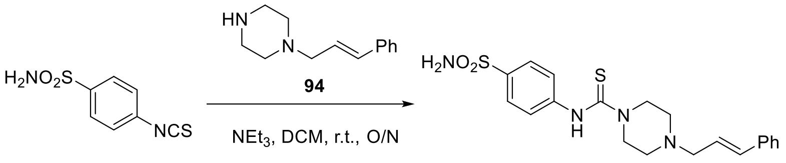

Scheme S15

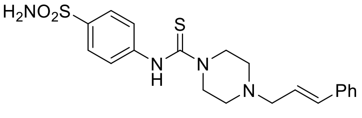

35

4-cinnamyl-*N*-(4-sulfamoylphenyl)piperazine-1-carbothioamide, 35 – **93** (58 mg, 0.22 mmol, 1 eq.) was purged with argon, dissolved in anhydrous DCM (3 mL) and cooled to 0 °C. **94** (50 mg, 0.22 mmol, 1 eq.) was added followed by NEt_3 (39 µL, 0.22 mmol, 1 eq.). The reaction was warmed to r.t., and left to stir O/N. The reaction was concentrated under reduced pressure and purified *via* column chromatography to afford **35** (63 mg, 65%), as a white solid. $\mathbf{R_f}$ = 0.20 (DCM/MeOH 94:6); **^{1}H NMR (400 MHz, $(CD_3)_2SO$)** δ ppm 9.55 (s, 1H), 7.76 – 7.68 (m, 2H), 7.51 – 7.42 (m, 4H), 7.38 – 7.29 (m, 2H), 7.29 – 7.20 (m, 3H), 6.58 (d, *J* = 15.9 Hz, 1H), 6.34 (dt, *J* = 15.9, 6.6 Hz, 1H), 3.93 (t, *J* = 4.9 Hz, 4H), 3.17 (dd, *J* = 6.6, 1.4 Hz, 2H), 2.51 (d, *J* = 10.7 Hz, 4H); **^{13}C{^{1}H} NMR (101 MHz, $(CD_3)_2SO$)** δ 181.1, 144.3, 138.8, 136.6, 132.4, 128.6 (2C), 127.5, 126.6, 126.3 (2C), 125.7 (2C), 123.9 (2C), 59.7, 52.3 (2C), 48.3 (2C); **HRMS (m/z)**: $[M+H]^+$ calcd. 417.1413 for $C_{20}H_{25}N_4O_2S_2$; found 417.1421; $[M+Na]^+$ calcd. 439.1225 for $C_{20}H_{24}N_4NaO_2S_2$; found 439.1233.

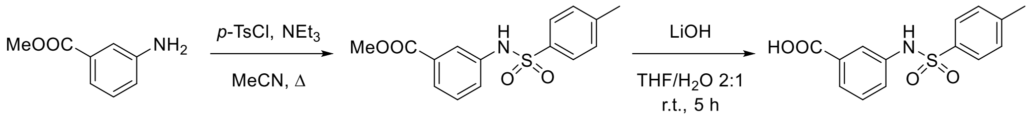

95 **36** (1.1 g, 55%) **96** (477 mg, quant.)

Scheme S16

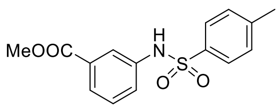

36

Methyl 3-((4-methylphenyl)sulfonamido)benzoate, 36 – **95** (1 g, 6.62 mmol, 1 eq.) and *p*-TsCl (2.53 g, 13.2 mmol, 2 eq.) were purged with argon and dissolved in anhydrous MeCN (30 mL). NEt_3 (0.92 mL, 6.62 mmol, 1 eq.) was added and the reaction was refluxed for 24 hours. The reaction was concentrated under reduced pressure, re-dissolved in DCM (100 mL), washed with sat. NH_4Cl (100 mL), sat. brine (100 mL), dried over $MgSO_4$ and concentrated under reduced pressure. The crude product was purified by column chromatography to afford **36** (1.12 g, 55%) as a white solid. **R_f** = 0.3 (Hex/EA 7:3); **^{1}H NMR (500 MHz, $(CD_3)_2SO$):** δ ppm 10.49 (br s, 1H), 7.71, (dt, *J* = 1.9, 0.9 Hz, 1H), 7.66 – 7.62 (m, 2H), 7.59 (dt, *J* = 6.9, 1.7 Hz, 1H), 7.41 – 7.31 (m, 4H), 3.81 (s, 3H), 2.32 (s, 3H); **^{13}C{^{1}H} NMR (126 MHz, $(CD_3)_2SO$):** δ ppm 165.7, 143.5, 138.3, 136.3, 130.5, 129.8 (2C), 129.7, 126.7 (2C), 124.5, 124.2, 120.0, 52.3, 20.9; **HRMS (*m/z*):** $[M+Na]^+$ calcd. 328.0614 For $C_{15}H_{15}NNaO_4S$; found 328.0619.

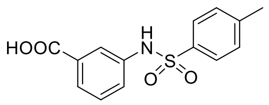

96

3-((4-methylphenyl)sulfonamido)benzoic acid, 96 – **36** (500 mg, 1.64 mmol, 1 eq.) was dissolved in THF (5 mL) and $LiOH.H_2O$ (276 mg, 6.56 mmol, 4 eq.) was dissolved in H_2O (2.5 mL) and added in dropwise. The reaction was stirred at r.t., for 5 h. and then concentrated under reduced pressure. The residue was suspended in ethyl acetate (50 mL) and washed with sat. NH_4Cl (50 mL) and the pH was adjusted to pH 3 with 1M HCl. The aqueous phase was extracted with ethyl acetate (3 x 50 mL). The combined organic phases were washed with sat. brine (50 mL), dried over $MgSO_4$ and concentrated under reduced pressure to afford **96** (477 mg, quant.) as a white solid. **^{1}H NMR (400 MHz, $(CD_3)_2SO$)**: δ ppm 11.00 – 10.28 (br s, 1H), 7.72 (t, *J* = 1.6 Hz, 1H), 7.65 (d, *J* = 8.3 Hz, 2H), 7.55 (dt, *J* = 7.3, 1.5 Hz, 1H), 7.38 – 7.27 (m, 4H); **^{13}C{^{1}H} NMR (101 MHz, $(CD_3)_2SO$):** δ ppm 167.0, 143.3, 138.2, 136.6, 129.7 (3C), 129.2, 126.7 (2C), 124.7, 123.7, 120.6, 21.0; **HRMS (*m/z*):** $[M+H]^+$ calcd. 292.0638 for $C_{14}H_{14}NO_4S$; found 292.0635; $[M+Na]^+$ calcd. 314.0457 for $C_{14}H_{13}NNaO_4S$; found 314.0453.

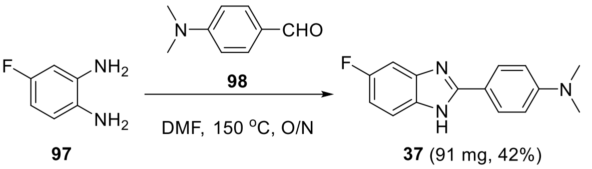

Scheme S17

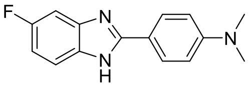

37

4-(5-fluoro-1*H*-benzo[*d*]imidazol-2-yl)-*N,N*-dimethylaniline, 37 – **97** (100 mg, 0.80 mmol, 1 eq.) and **98** (236 mg, 1.6 mmol, 2 eq.) were dissolved in anhydrous DMF (5 mL) and heated to 150 °C (open to air) and stirred O/N. The reaction was diluted in H_2O (50 mL) and the resultant precipitate collected *via* vacuum filtration and dried under high vacuum. The crude product was purified by column chromatography to afford **37** (91 mg, 42%) as a beige solid. **R_f** = 0.3 (DCM/MeOH/NH_4OH 96:3:1); **^{1}H NMR (400 MHz, CD_3CO_2D):** δ ppm 7.85 (d, *J* = 9.2 Hz, 2H), 7.56 (dd, *J* = 8.9, 4.4 Hz, 1H), 7.29 (dd, *J* = 8.4, 2.3 Hz, 1H), 7.13 (td, *J* = 9.3, 2.4 Hz, 1H), 6.65 (d, *J* = 9.2 Hz, 2H), 3.03 (s, 6H); **^{19}F{^{1}H} NMR (471 MHz, CD_3CO_2D):** δ ppm -116.57 (s, 1F); **^{13}C{^{1}H} NMR (101 MHz, CD_3CO_2D):** δ ppm 161.5 (d, *J* = 242 Hz), 154.5, 151.9 (d, *J* = 1.5 Hz), 133.0 (d, *J* = 13 Hz), 130.2 (2C), 129.1, 115.5 (d, *J* = 10 Hz), 114.3 (br d, *J* = 26 Hz), 112.6 (2C), 108.3, 101.1 (d, *J* = 29 Hz), 40.0 (2C); **HRMS (*m/z*):** $[M+H]^+$ calcd. 256.1245 For $C_{15}H_{15}FN_3$; found 256.1250.

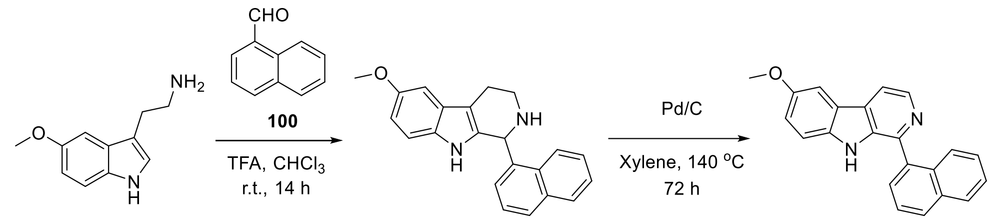

99 101 (430 mg, 50%) 38 (124 mg, 30%)

Scheme S18

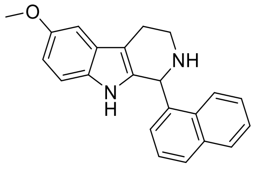

101

6-methoxy-1-(naphthalen-1-yl)-2,3,4,9-tetrahydro-1*H*-pyrido[3,4-*b*]indole, 101 – **99** (500 mg, 2.6 mmol, 1 eq.) was dissolved in anhydrous chloroform (5 mL) and purged with argon. **100** (0.36 mL, 2.6 mmol, 1 eq.) was added dropwise to the brown solution followed by TFA (0.35 mL, 4.6 mmol, 1.7 eq.) after which solution turned black. The reaction was stirred O/N at r.t. The crude organic phase was washed with H_2O (50 mL), dried over $MgSO_4$ and concentrated under reduced pressure. The crude residue was purified by column chromatography to afford **101** (430 mg, 50%) as an oil. **R*f*** = 0.4 (DCM/Acetone 60:40); **^{1}H NMR (400 MHz, CDCl$_3$):** δ ppm 8.12 (br s, 1H), 7.90 (br d, *J* = 8.1 Hz, 1H), 7.85 (br d, *J* = 8.2 Hz, 1H), 7.61 (br s, 1H), 7.5 (m, 2H), 7.35 (br t, *J* = 7.6 Hz, 1H), 7.26 (br s, 1H), 7.03 (br d, *J* = 8.7 Hz, 1H), 6.98 (d, *J* = 1.7 Hz, 1H), 6.80 (dd, *J* = 8.7, 2.0 Hz, 1H), 6.10 (br s, 1H), 3.87 (s, 3H), 3.02 (m, 4H), 2.78 (m, 1H); **^{13}C{^{1}H} NMR (101 MHz, CDCl$_3$):** δ 154.4, 134.2, 133.0, 131.9, 131.4, 131.3, 130.1, 129.1, 128.1, 127.4, 127.2, 126.4, 125.4, 122.9, 112.4, 111.9, 109.7, 100.6, 56.1, 31.0, 29.4, 20.5.

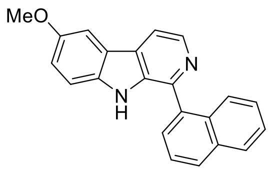

38

6-methoxy-1-(naphthalen-1-yl)-9H-pyrido[3,4-b]indole, 38 – **101** (430 mg, 1.3 mmol, 1.eq.) was dissolved in *o*-xylene (10 mL). Pd/C (140 mg, 0.13 mmol, 0.1 eq) was added in one portion and the reaction was heated to 140 °C for 3 days (open to air). The reaction was cooled, filtered over a pad of celite and concentrated under reduced pressure. The crude residue was purified *via* column chromatography to afford **38** (124 mg, 30%) as an amber solid. **R*f*** = 0.2 (DCM/Acetone 95:5); **^{1}H NMR (400 MHz, (CD$_3$)$_2$SO)**: δ ppm 10.94 (s, 1H), 8.47 (d, *J* = 5.3 Hz, 1H), 8.19 (d, *J* = 5.3 Hz, 1H), 8.11 (d, *J* = 7.5 Hz, 1H), 8.07 (d, *J* = 8.2 Hz, 1H), 7.85 (d, *J* = 2.6 Hz, 1H), 7.75 (dd, *J* = 7.1, 1.6 Hz, 1H), 7.72 (d, *J* = 7.8 Hz, 1H), 7.68 (d, *J* = 7.6 Hz, 1H), 7.56 (ddd, *J* = 8.2, 6.9, 1.2 Hz, 1H), 7.44 (ddd, *J* = 8.4, 6.9, 1.2 Hz, 1H),

7.40 (d, *J* = 8.9 Hz, 1H), 7.16 (dd, *J* = 8.8, 2.6 Hz, 1H), 3.88 (s, 3H); **$^{13}C\{^1H\}$ NMR (101 MHz, $(CD_3)_2SO$)**: δ ppm 153.4, 142.8, 137.4, 135.8, 135.62, 135.2, 133.6, 131.2, 128.6, 128.34, 128.28, 127.4, 126.3, 126.0, 125.7, 125.6, 121.1, 118.2, 114.0, 113.1, 103.5, 55.7; **HRMS (*m/z*):** $[M+H]^+$ calcd. 325.1335 For $C_{22}H_{17}N_2O$; found 325.1343.

DSF

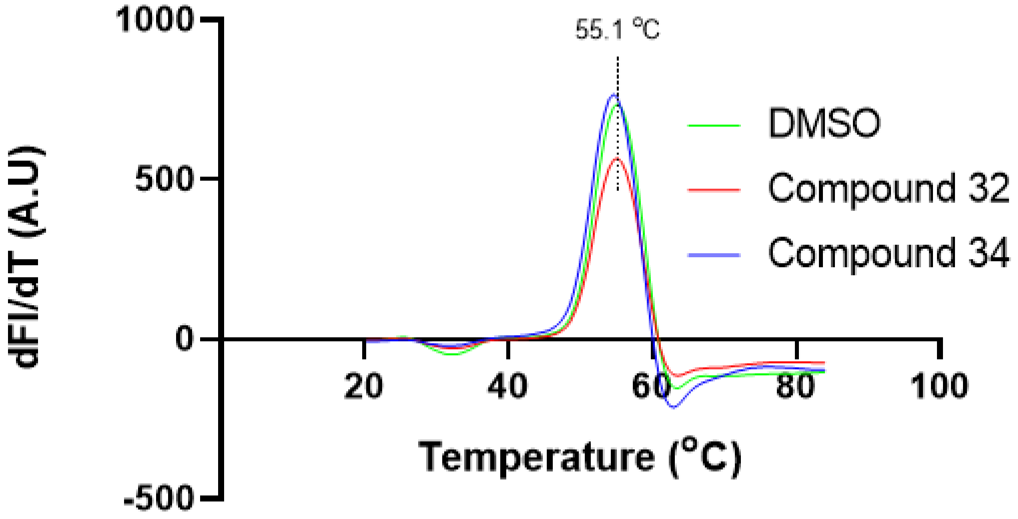

Figure S4 – Assessment of compound induced protein stabilisation by DSF. First derivative of temperature dependent fluorescence, representative curves of DMSO control, compounds **32** and **34**. β-catenin $ARD_{148\text{-}662}$ (8 μM), SYPRO orange dye reporter (10x), DMSO (5% v/v), no small molecule (control) or small molecule (125 μM). β-catenin control melting temperature T_m = 55.1°C.

ITC

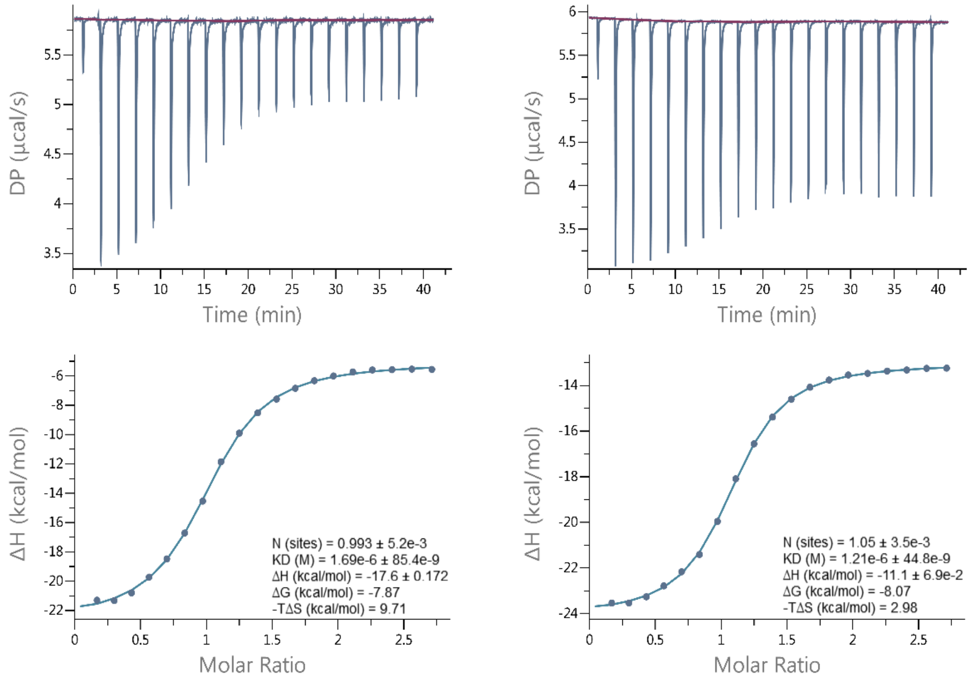

Figure S5 – Positive control ITC trace showing binding of known peptides TCF4$_{13-27}$ (left), BCL9$_{348-376}$ (right), concentration = 400 µM vs β-catenin ARD$_{148-662}$ concentration = 30 µM

Table S2 – ITC trace of small molecules **6**, **10** and **11** vs β-catenin ARD$_{148-662}$ at given concentrations

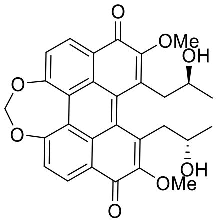 **CGP049090 - 6**	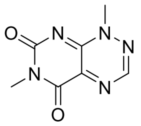 **PKF118-310 - 10**	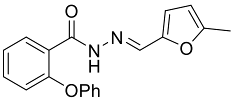 **PNU74654 - 11**
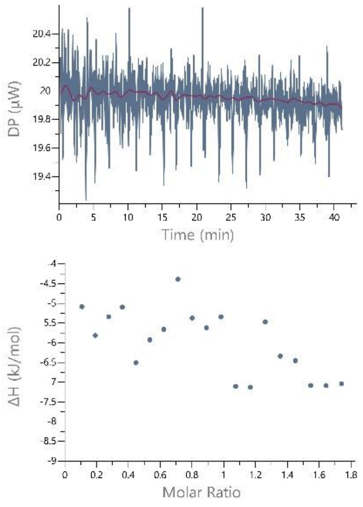	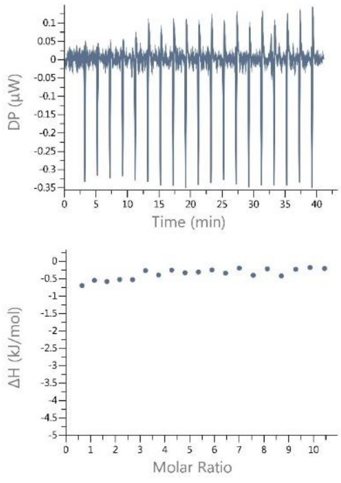	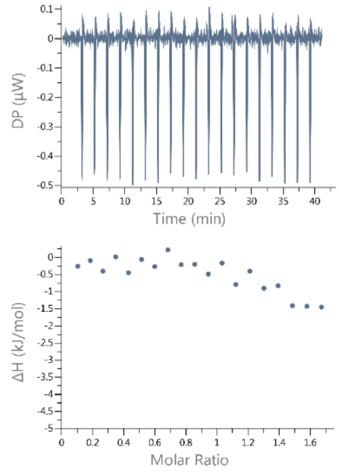
[**6**] = 250 µM; [β-catenin] = 30 µM	[**10**] = 1 mM; [β-catenin] = 30 µM	[**11**] = 240 µM; [β-catenin] = 30 µM

Table S3 – ITC trace of small molecules **12**, **15** and **17** vs β-catenin $ARD_{148\text{-}662}$ at given concentrations

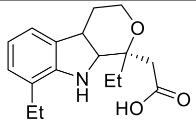 **SDX-101 - 12**	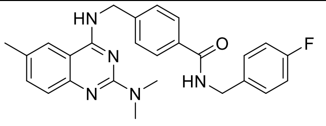 **15**	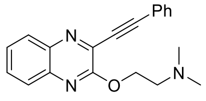 **17**
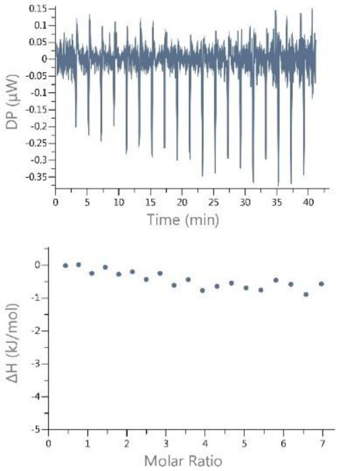	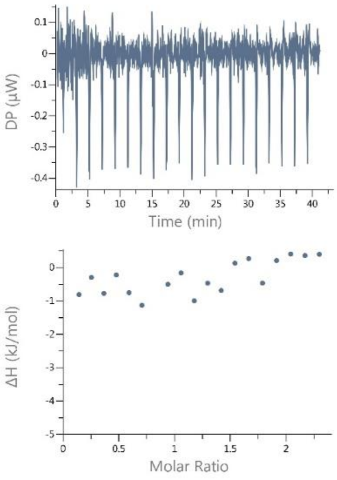	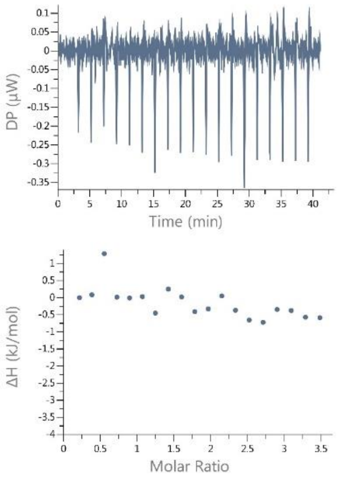
[**12**] = 500 µM; [β-catenin] = 30 µM	[**15**] = 250 µM; [β-catenin] = 20 µM	[**17**] = 500 µM; [β-catenin] = 30 µM

Table S4 – ITC trace of small molecules **18** - **20** vs β-catenin $ARD_{148\text{-}662}$ at given concentrations

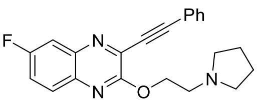 **18**	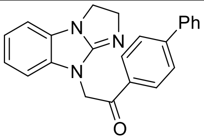 **CCT031374 - 19**	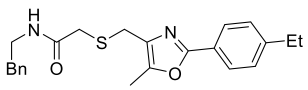 **iCRT3 - 20**
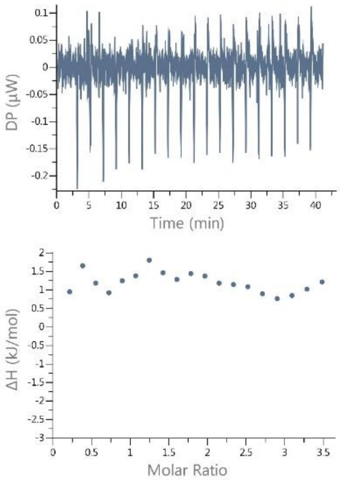	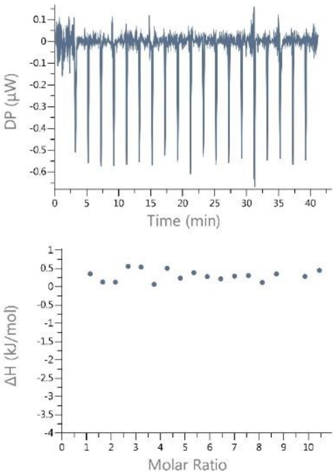	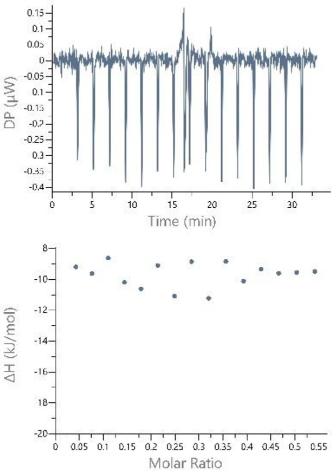
[**18**] = 500 µM; [β-catenin] = 30 µM	[**19**] = 1 mM; [β-catenin] = 30 µM	[**20**] = 100 µM; [β-catenin] = 30 µM

Table S5 – ITC trace of small molecules **22** - **24** vs β-catenin $ARD_{148\text{-}662}$ at given concentrations

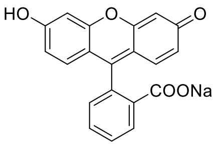 **Fluorescein - 22**	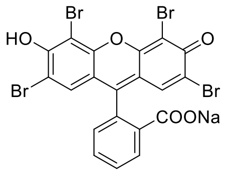 **Eosin Y - 23**	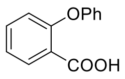 **24**
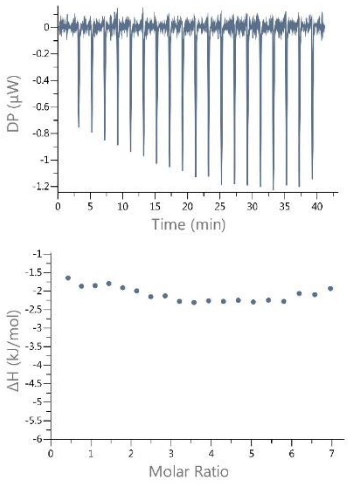	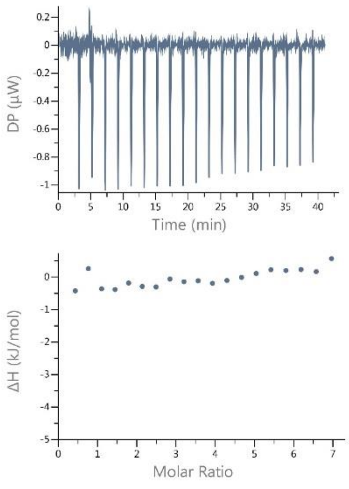	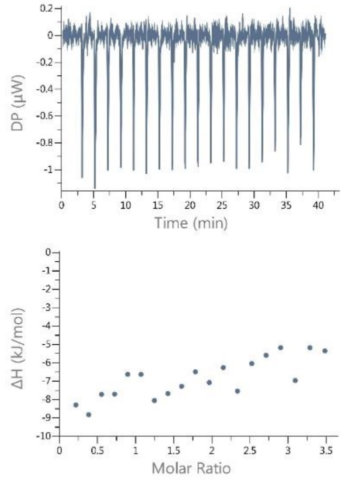
[**22**] = 300 μM; [β-catenin] = 30 μM	[**23**] = 300 μM; [β-catenin] = 30 μM	[**24**] = 1 mM; [β-catenin] = 30 μM

Table S6 – ITC trace of small molecules **26**, **28** and **29** vs β-catenin $ARD_{148\text{-}662}$ at given concentrations

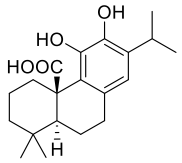 **Carnosic Acid - 26**	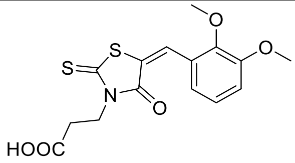 **L338192 - 28**	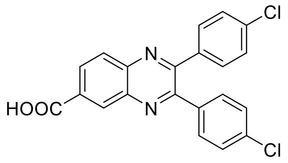 **R999636 - 29**
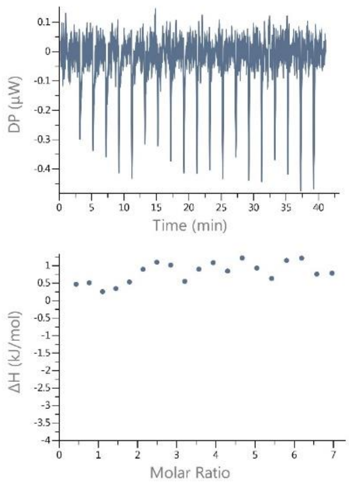	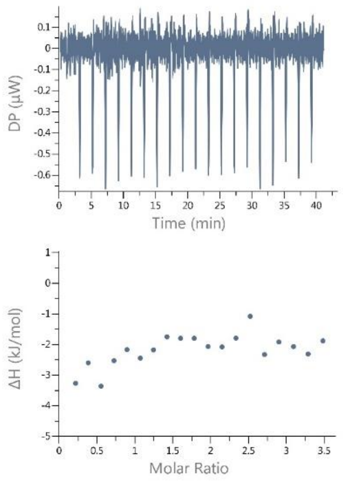	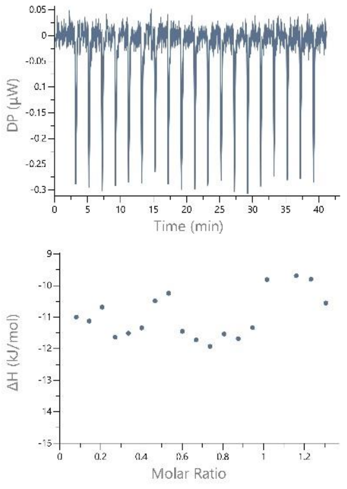
[**26**] = 1 mM; [β-catenin] = 30 μM	[**28**] = 500 μM; [β-catenin] = 30 μM	[**29**] = 125 μM; [β-catenin] = 30 μM

Table S7 – ITC trace of small molecules **30**, **32** and **33** vs β-catenin $ARD_{148\text{-}662}$ at given concentrations

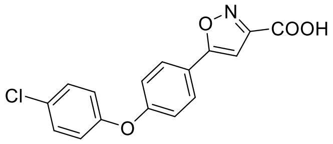 **T155535 - 30**	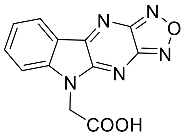 **32**	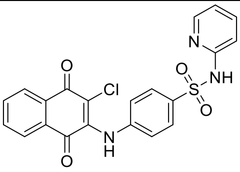 **BC-23 - 33**
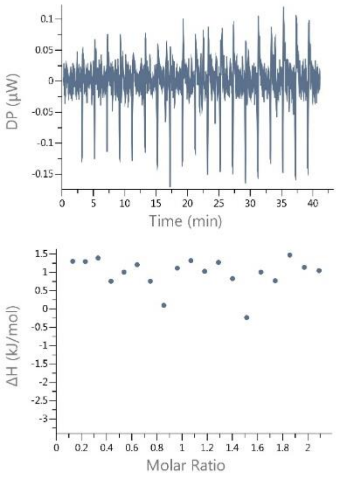	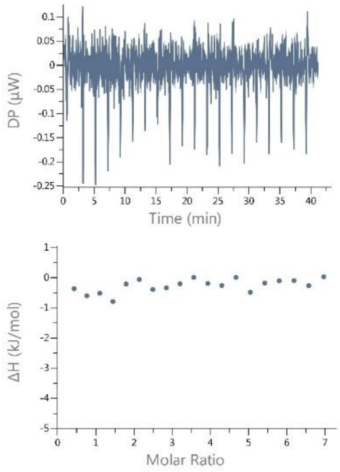	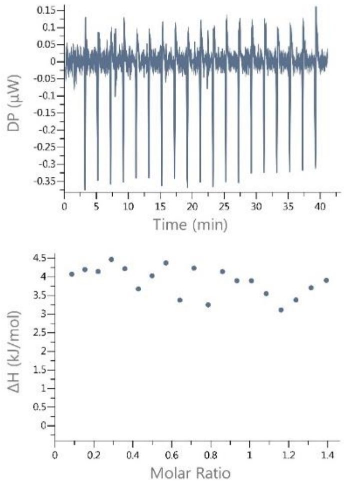
[**30**] = 330 µM; [β-catenin] = 30 µM	[**32**] = 300 µM; [β-catenin] = 30 µM	[**33**] = 200 µM; [β-catenin] = 30 µM

Table S8 – ITC trace of small molecules **34** - **36** vs β-catenin ARD$_{148-662}$ at given concentrations

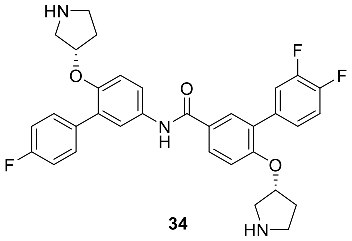 **34**	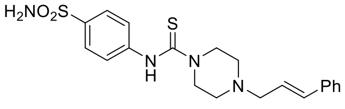 **LF3 - 35**	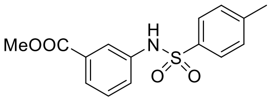 **MSAB - 36**
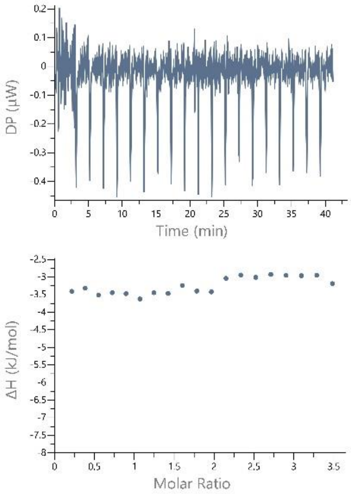	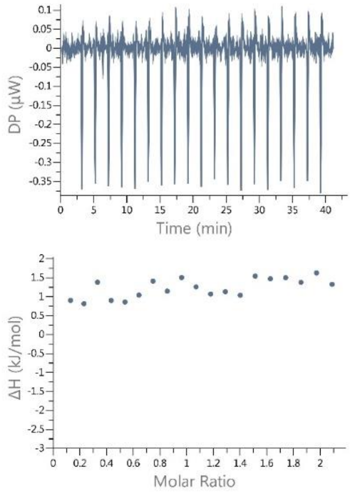	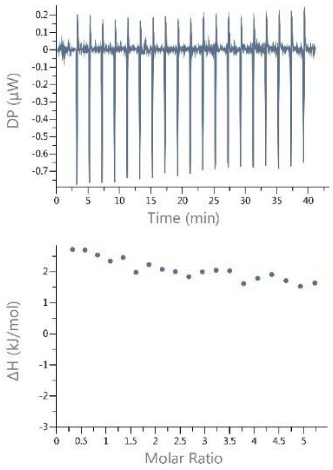
[**34**] = 500 µM; [β-catenin] = 30 µM	[**35**] = 300 µM; [β-catenin] = 30 µM	[**36**] = 500 µM; [β-catenin] = 30 µM

Table S9 – ITC trace of small molecules **96**, **37** and **38** vs β-catenin ARD$_{148-662}$ at given concentrations

<table>
<tr>
<td>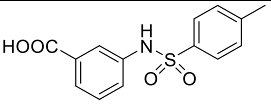

96</td>
<td>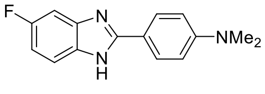

HI-B1 - 37</td>
<td>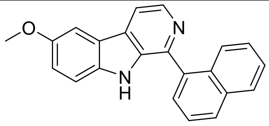

SP141 - 38</td>
</tr>
<tr>
<td>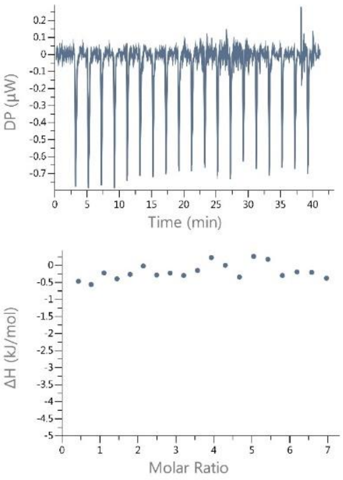
</td>
<td>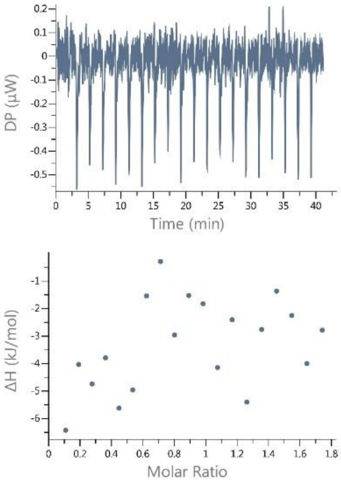
</td>
<td>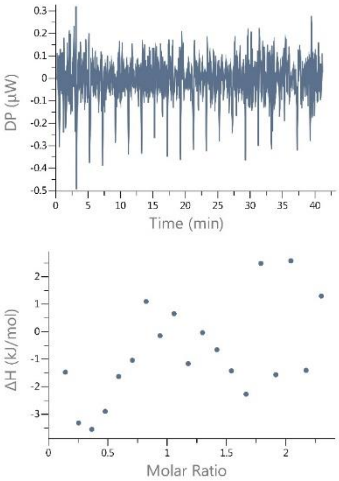
</td>
</tr>
<tr>
<td>[96] = 1 mM; [β-catenin] = 30 μM</td>
<td>[37] = 250 μM; [β-catenin] = 30 μM</td>
<td>[38] = 330 μM; [β-catenin] = 30 μM</td>
</tr>
</table>

Figure S6 – NMR spectra and HPLC traces

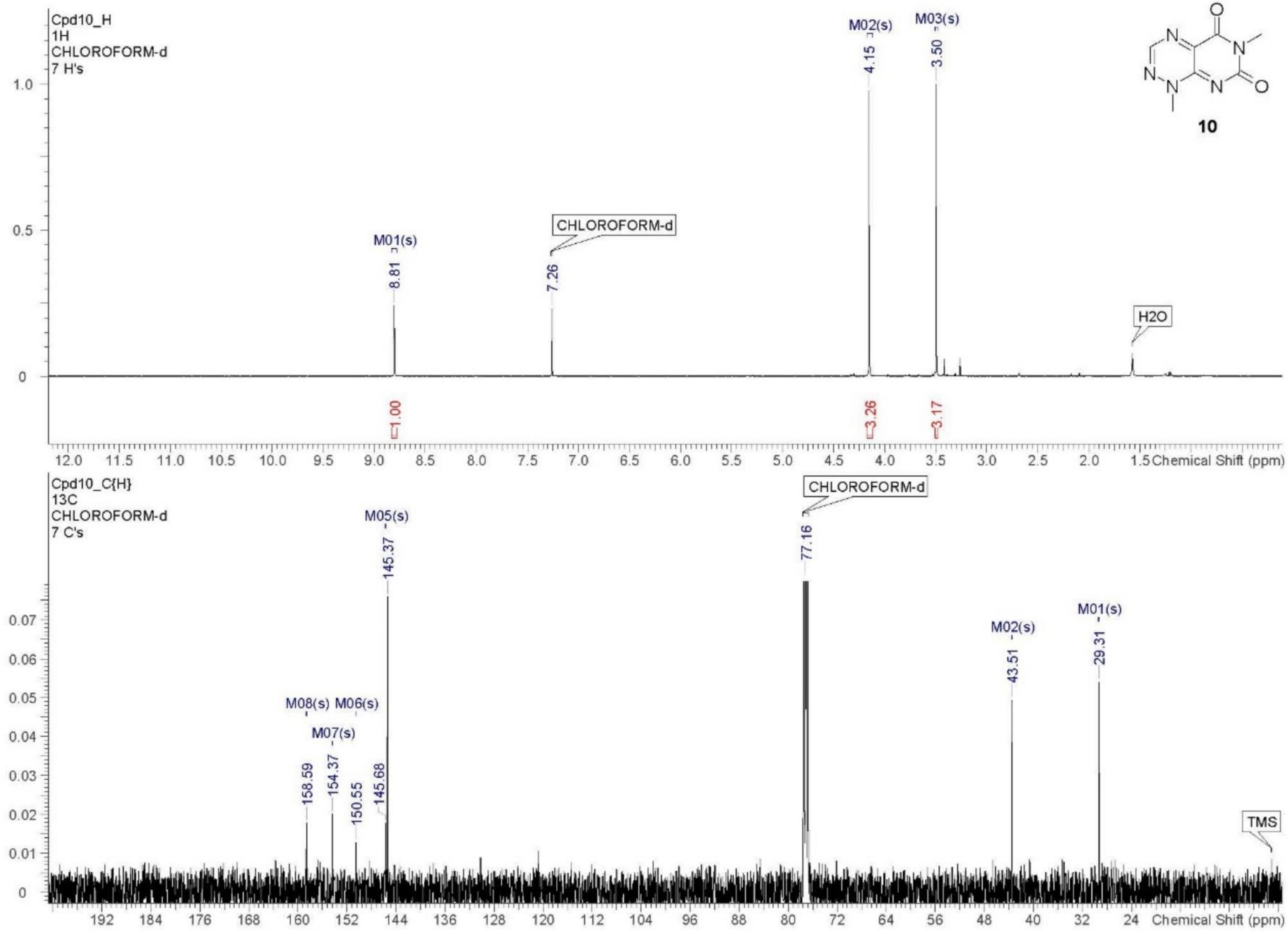

Top 5 Peak Report - UV

Sample ID: MAM4-025 Column
Group: Baud, M
Acquisition Date: 27/11/2019 17:25:21
Experiment: BLUE ESIPOSNEG C18 5 min
Filename: MAM4_025_Column__Michael_McCoy__Baud_M__72913.pdf

Submitter: Michael McCoy
Project: RP LC C18 custom
Instrument: Blue RP UHPLC-MS (B30:1023)

Absorbance, NL 2.920E06

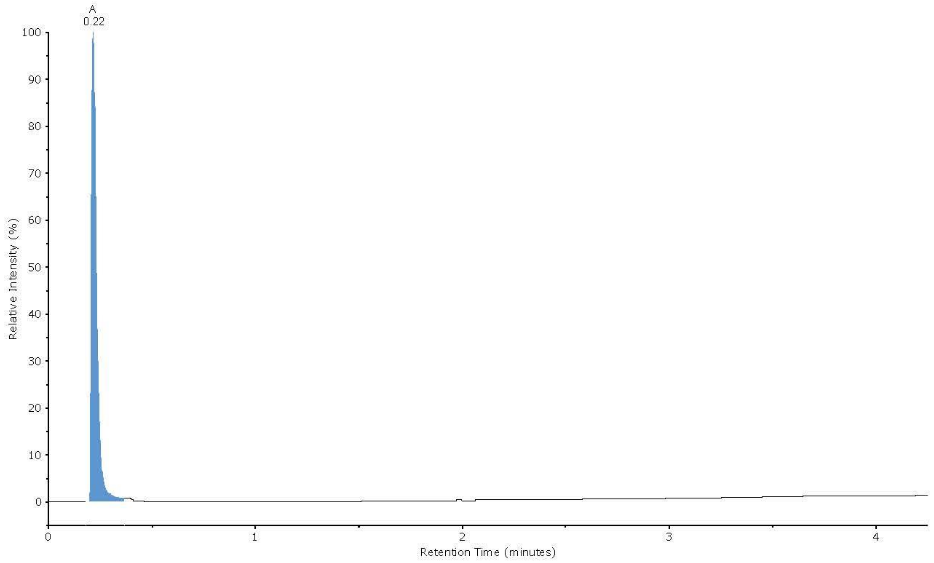

	RT Mins	Height	Height %	Area	Absolute Area %	Relative Area %
A	0.22	2910032	100.00	5158137	100.00	100.00

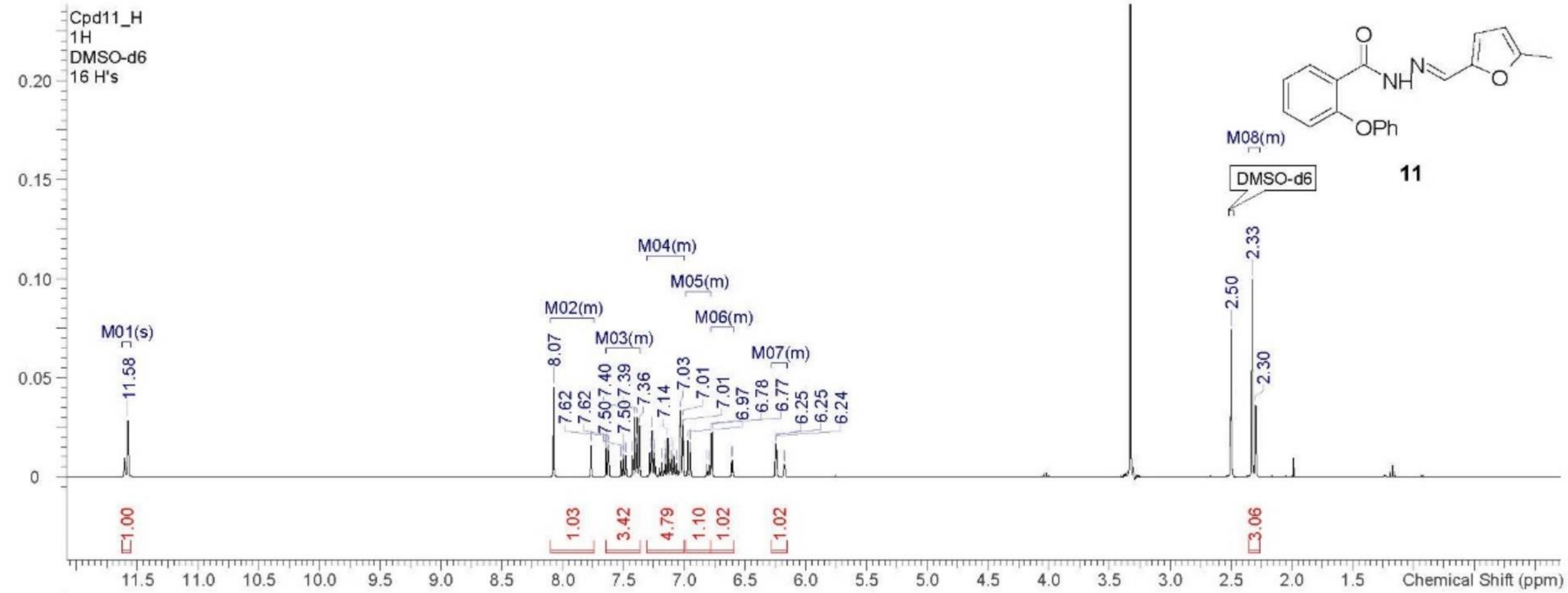
Cpd11_H
1H
DMSO-d6
16 H's
M01(s)
11.58
M02(m)
8.07
M03(m)
M04(m)
M05(m)
M06(m)
M07(m)
M08(m)
DMSO-d6
2.50
2.33
2.30
11
OPh
Chemical Shift (ppm)

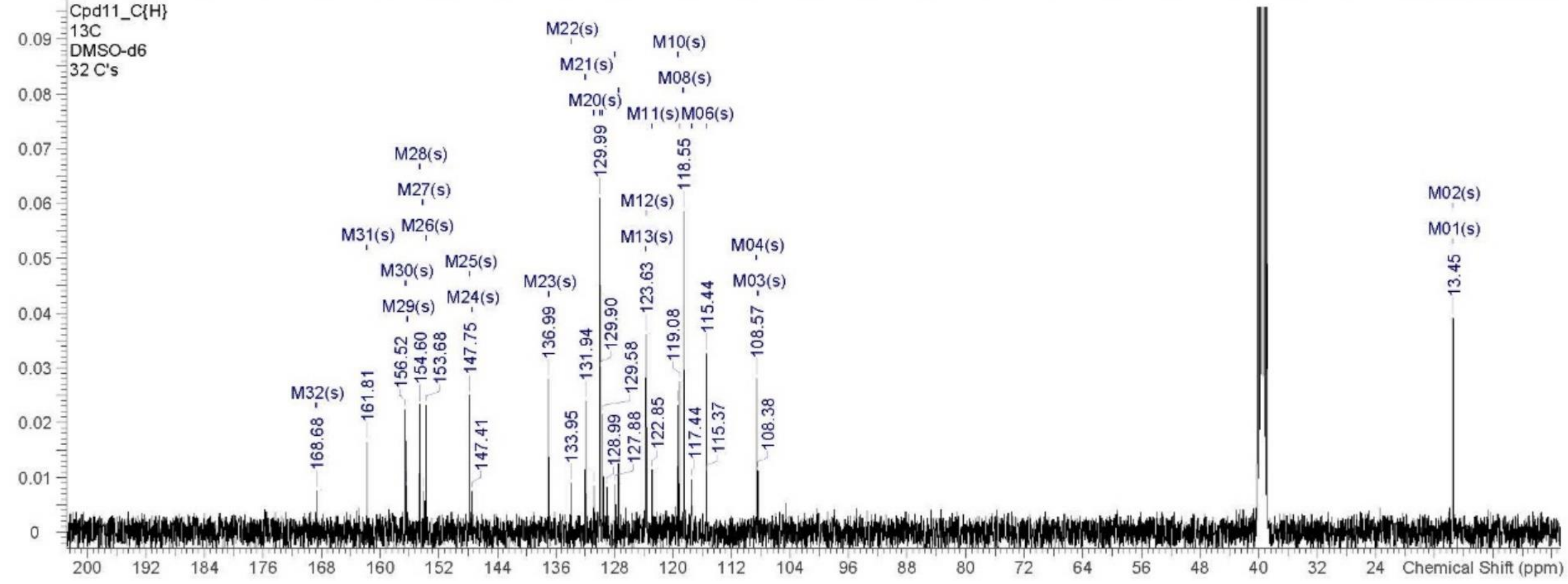
Cpd11_C{H}
13C
DMSO-d6
32 C's
168.68
161.81
156.52
154.60
153.68
147.75
147.41
136.99
133.95
131.94
129.99
129.90
129.58
128.99
127.88
123.63
122.85
119.08
118.55
117.44
115.44
115.37
108.57
108.38
13.45
Chemical Shift (ppm)

Chemistry - maXis HPLC-ESI Accurate Mass Report

Analysis Info

Analysis Name	D:\Data\Chemistry\2019\Apr\MAM2-DS-033_GB3_01_38374.d	Acquisition Date	03/04/2019 16:33:16
Method	soton lcms pos 120 to 1500.m	Operator	MSWEB@SOTON.AC.UK
Sample Name	MAM2-DS-033	Instrument / Ser#	maXis 17
Comment	Analyst: JMH		

Acquisition Parameter

Source Type	ESI	Ion Polarity	Positive	Set Nebulizer	2.0 Bar
Focus	Not active	Set Capillary	4000 V	Set Dry Heater	230 °C
Scan Begin	120 m/z	Set End Plate Offset	-500 V	Set Dry Gas	6.0 l/min
Scan End	1500 m/z	Set Collision Cell RF	300.0 Vpp	Set Divert Valve	Waste

Cmpd 1, 2.7 min

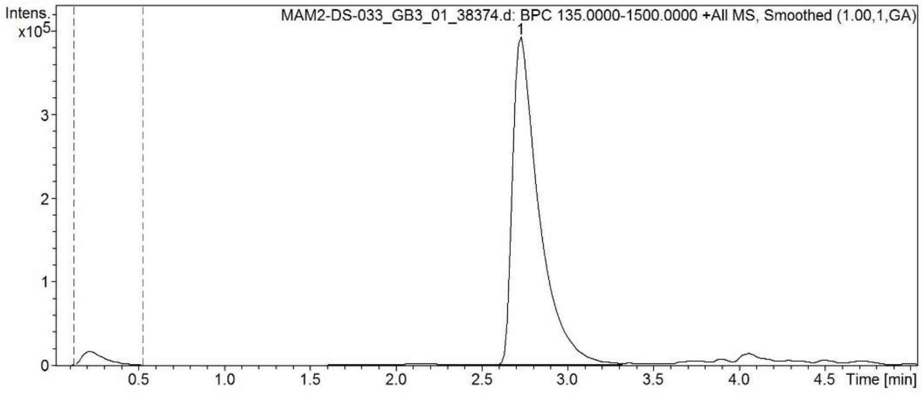

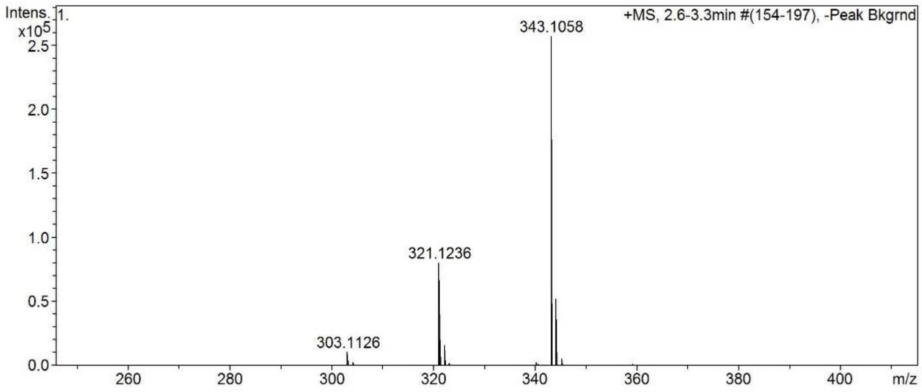

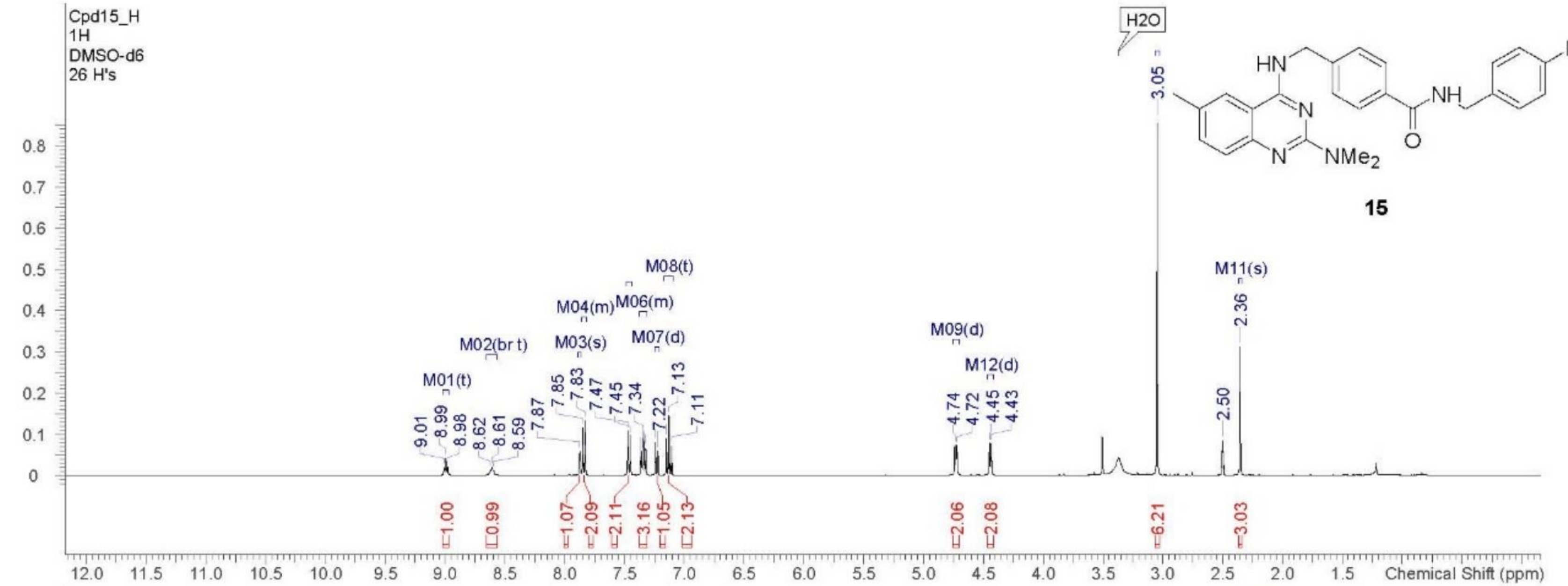
Cpd15_H
1H
DMSO-d6
26 H's
H2O
HN
N
N
NMe2
NH
O
F
15
M01(t)
M02(br t)
M03(s)
M04(m)
M06(m)
M07(d)
M08(t)
M09(d)
M12(d)
M11(s)
9.01 8.99 8.98 8.62 8.61 8.59 7.87 7.85 7.83 7.47 7.45 7.34 7.22 7.13 7.11
4.74 4.72 4.45 4.43
3.05
2.50
2.36
1.00 0.99 1.07 2.09 2.11 3.16 1.05 2.13 2.06 2.08 6.21 3.03
Chemical Shift (ppm)

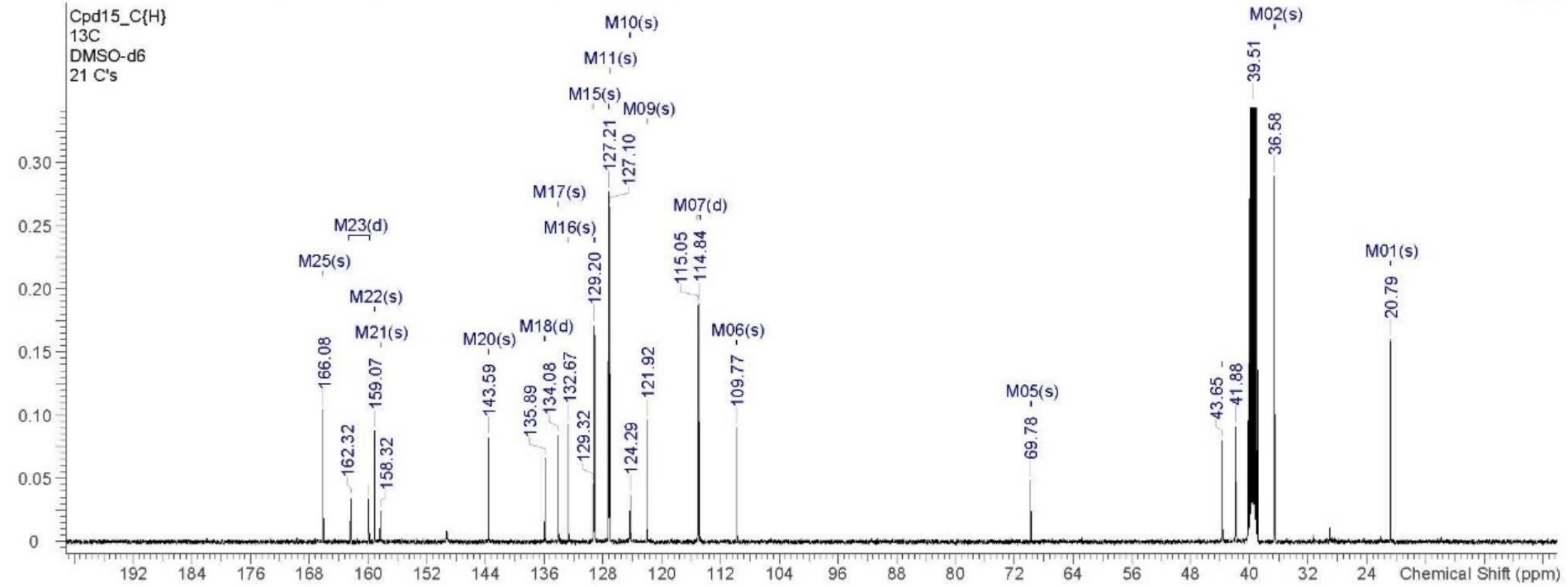
Cpd15_C{H}
13C
DMSO-d6
21 C's
M25(s)
M23(d)
M22(s)
M21(s)
M20(s)
M18(d)
M17(s)
M16(s)
M15(s)
M11(s)
M10(s)
M09(s)
M07(d)
M06(s)
M05(s)
M02(s)
M01(s)
166.08 162.32 159.07 158.32 143.59 135.89 134.08 132.67 129.32 129.20 127.21 127.10 124.29 121.92 115.05 114.84 109.77
69.78
43.65 41.88 39.51 36.58
20.79
Chemical Shift (ppm)

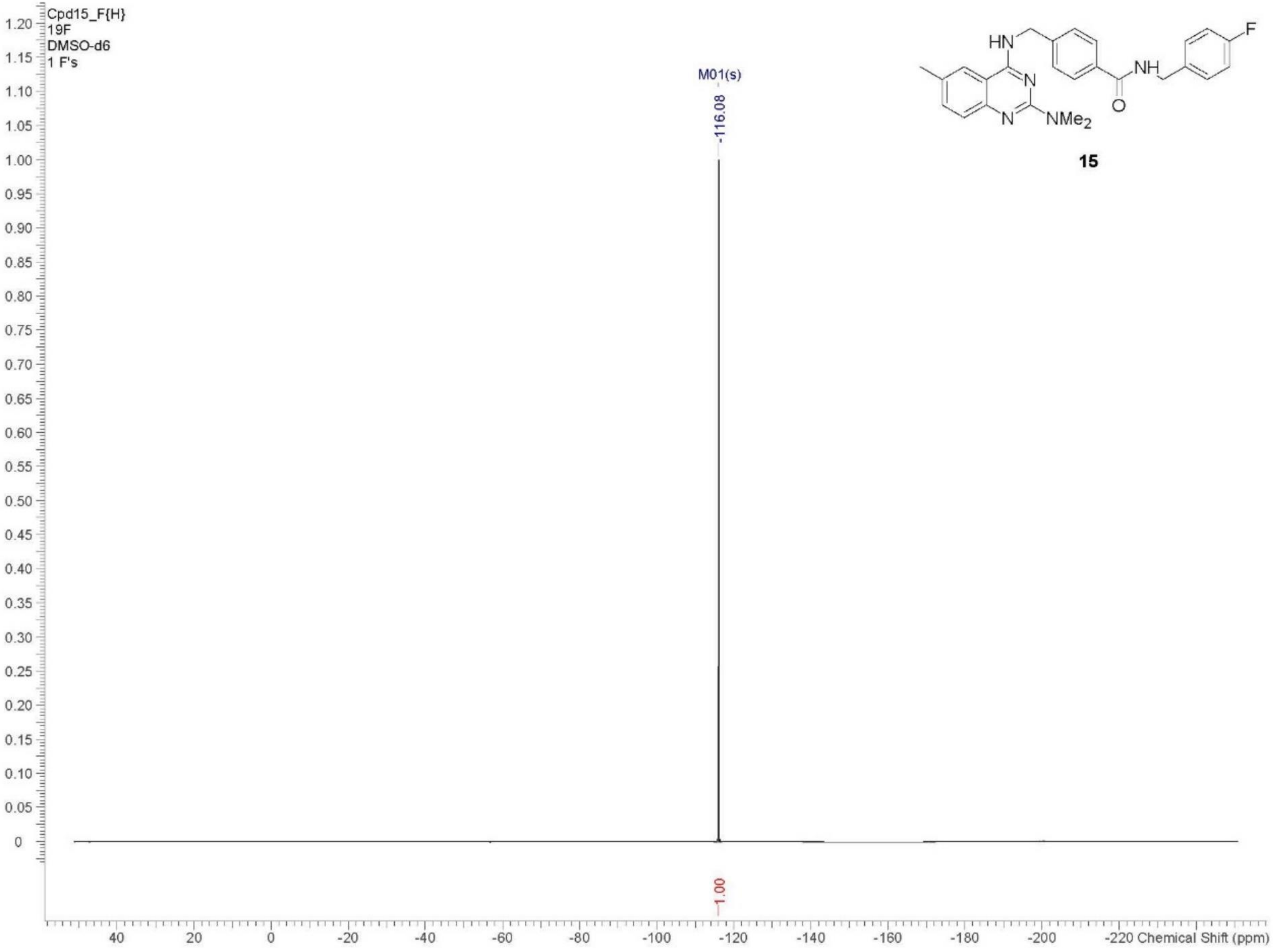
Cpd15_F{H}
19F
DMSO-d6
1 F's
M01(s)
-116.08
1.00
HN
N
NMe2
NH
O
F
15
Chemical Shift (ppm)

Chemistry - maXis HPLC-ESI Accurate Mass Report

Analysis Info

Analysis Name	D:\Data\Chemistry\2020\Jan\MAM4-027_RC6_01_39774.d	Acquisition Date	22/01/2020 17:42:10
Method	soton lcms pos 120 to 1500.m	Operator	MSWEB@SOTON.AC.UK
Sample Name	MAM4-027	Instrument / Ser#	maXis 17
Comment	Analyst: JMH		

Acquisition Parameter

Source Type	ESI	Ion Polarity	Positive	Set Nebulizer	2.0 Bar
Focus	Not active	Set Capillary	4000 V	Set Dry Heater	230 °C
Scan Begin	120 m/z	Set End Plate Offset	-500 V	Set Dry Gas	6.0 l/min
Scan End	1500 m/z	Set Collision Cell RF	300.0 Vpp	Set Divert Valve	Waste

+MS, 2.1min #(124)

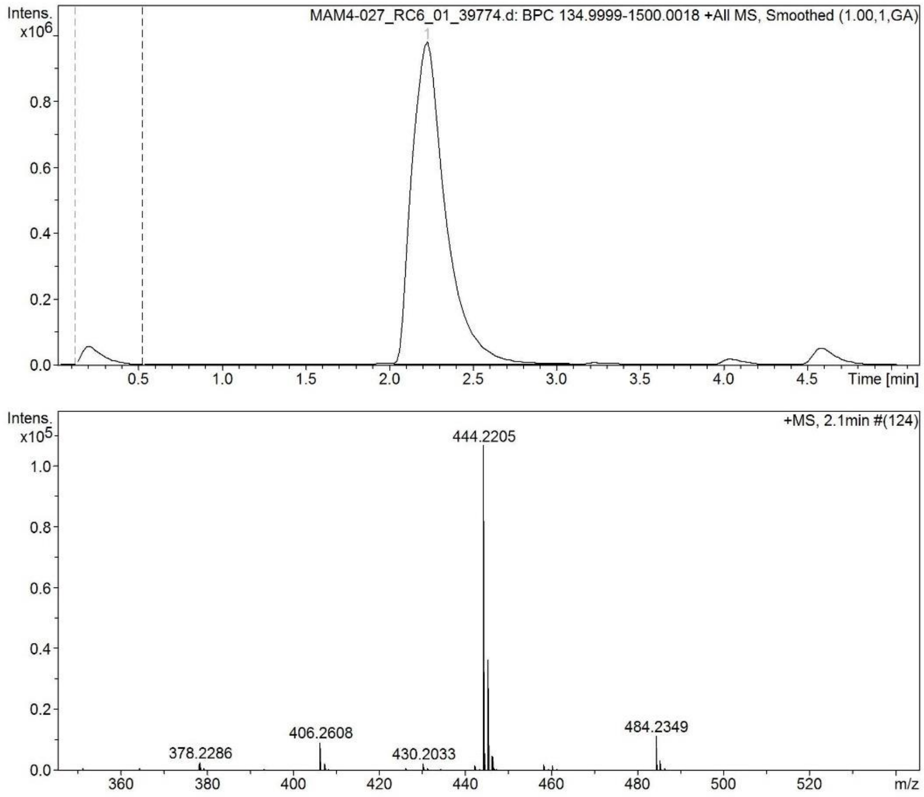

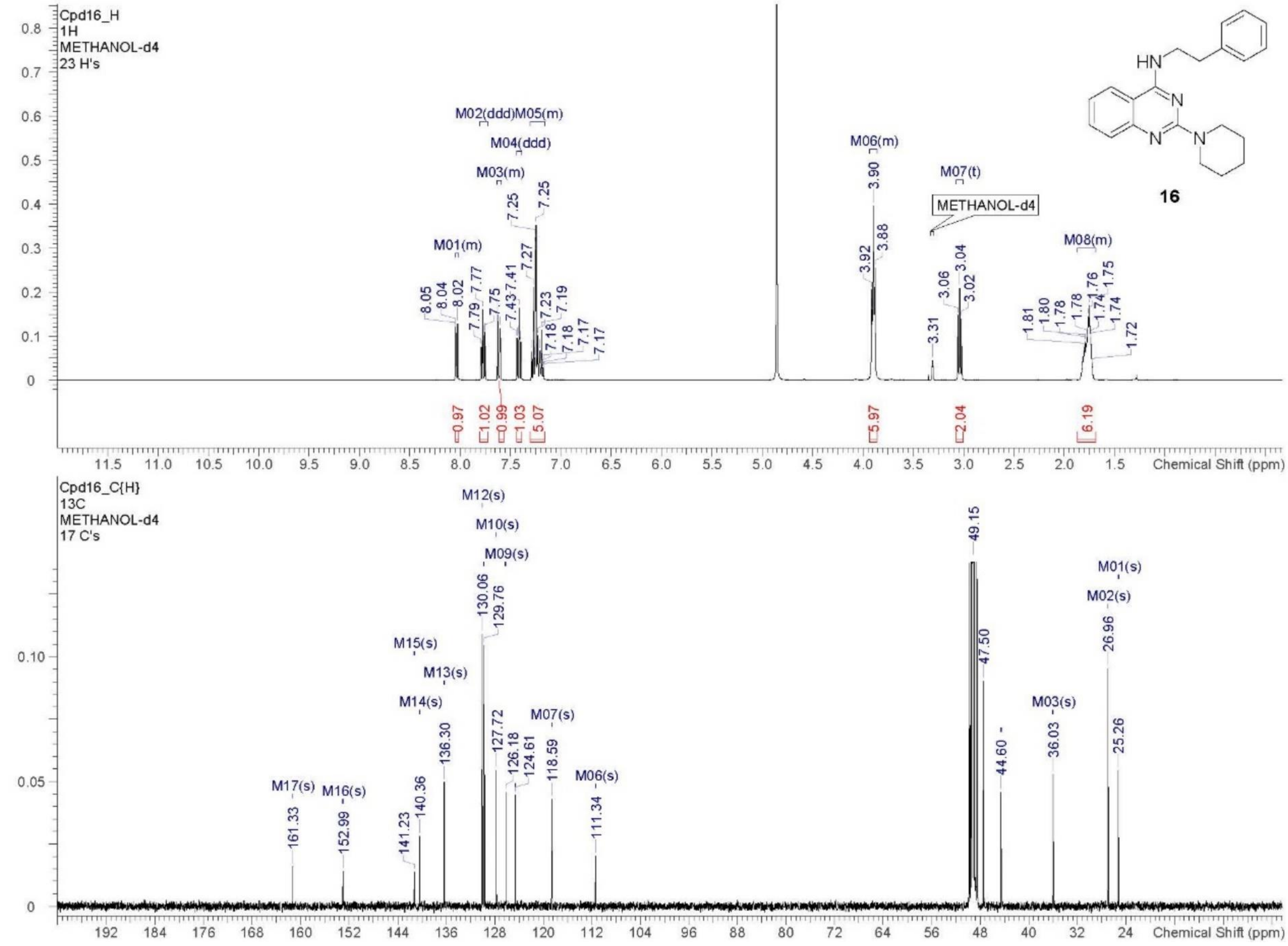
Cpd16_H
1H
METHANOL-d4
23 H's
M01(m)
M02(ddd)
M03(m)
M04(ddd)
M05(m)
M06(m)
M07(t)
M08(m)
METHANOL-d4
8.05
8.04
8.02
7.79
7.77
7.75
7.43
7.41
7.27
7.25
7.25
7.23
7.19
7.18
7.18
7.17
7.17
3.92
3.90
3.88
3.31
3.06
3.04
3.02
1.81
1.80
1.78
1.78
1.76
1.75
1.74
1.74
1.72
0.97
1.02
0.99
1.03
5.07
5.97
2.04
6.19
HN
N
N
N
16
Chemical Shift (ppm)
Cpd16_C{H}
13C
METHANOL-d4
17 C's
M01(s)
M02(s)
M03(s)
M06(s)
M07(s)
M09(s)
M10(s)
M12(s)
M13(s)
M14(s)
M15(s)
M16(s)
M17(s)
161.33
152.99
141.23
140.36
136.30
130.06
129.76
127.72
126.18
124.61
118.59
111.34
49.15
47.50
44.60
36.03
26.96
25.26
Chemical Shift (ppm)

Top 5 Peak Report - UV

Sample ID: MAM8-015 LRMS
Group: Baud, M
Acquisition Date: 01/10/2021 15:46:18
Experiment: BLUE ESIPOSNEG C18 5 min
Filename: MAM8_015_LRMS__Michael_McCoy__Baud_M__101007.pdf

Submitter: Michael McCoy
Project: RP LC C18 custom
Instrument: Blue RP UHPLC-MS (B30:1023)

Absorbance, NL 1.566E06

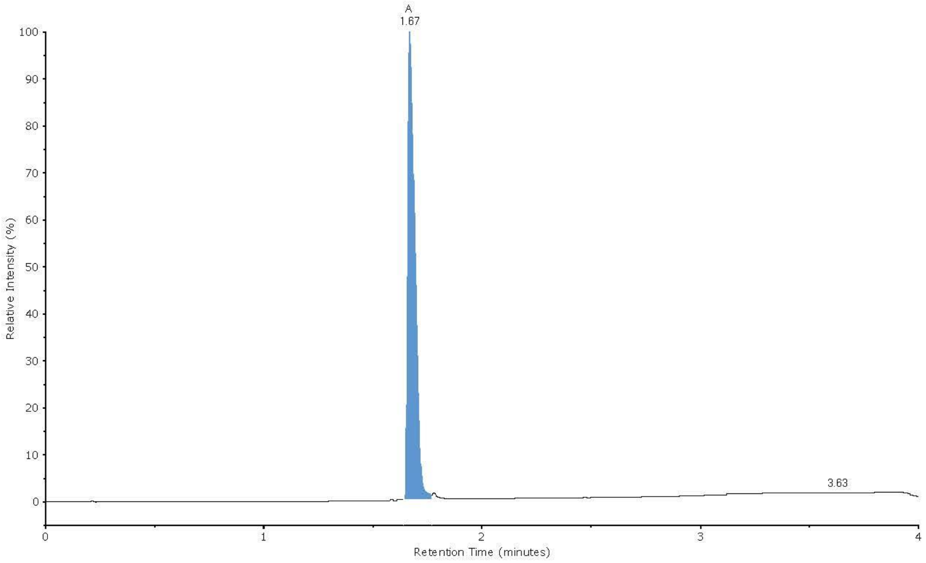

	RT Mins	Height	Height %	Area	Absolute Area %	Relative Area %
A	1.67	1551818	100.00	3727582	100.00	100.00

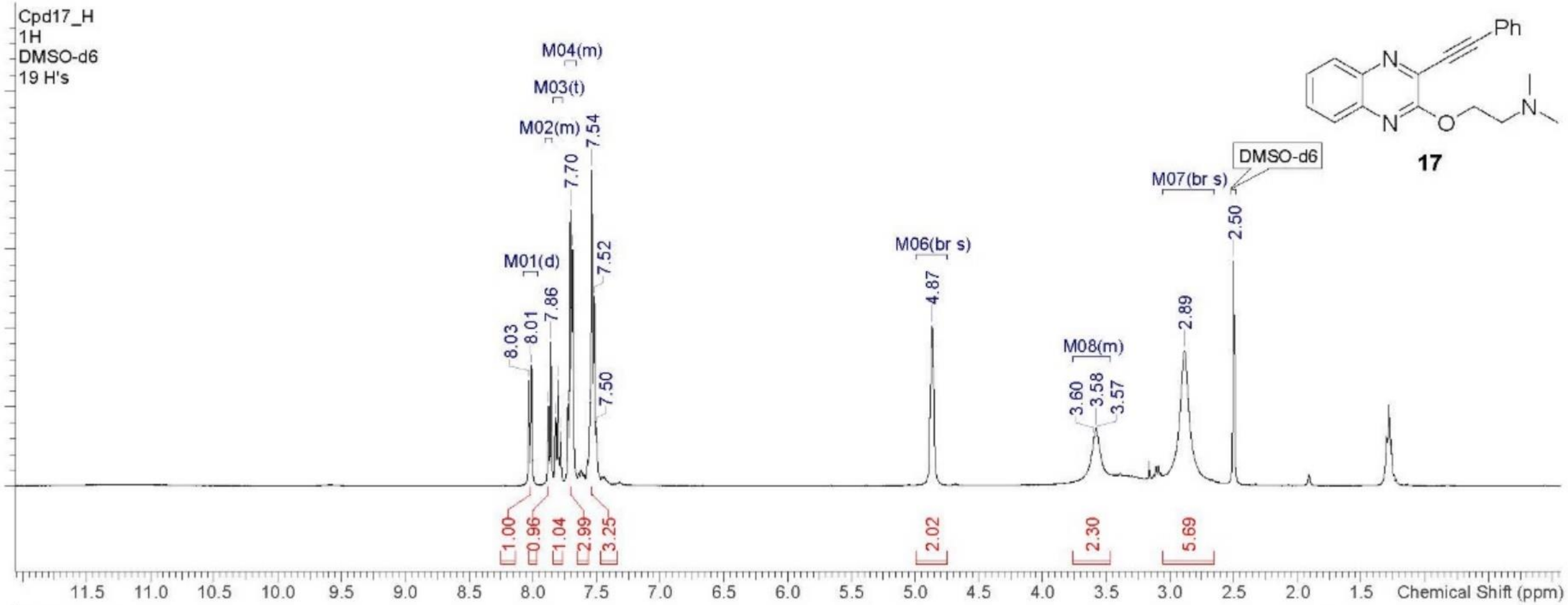
Cpd17_H
1H
DMSO-d6
19 H's
M01(d)
M02(m)
M03(t)
M04(m)
M06(br s)
M07(br s)
M08(m)
DMSO-d6
8.03
8.01
7.86
7.70
7.54
7.52
7.50
4.87
3.60
3.58
3.57
2.89
2.50
1.00
0.96
1.04
2.99
3.25
2.02
2.30
5.69
Chemical Shift (ppm)
Ph
N
N
O
N
17

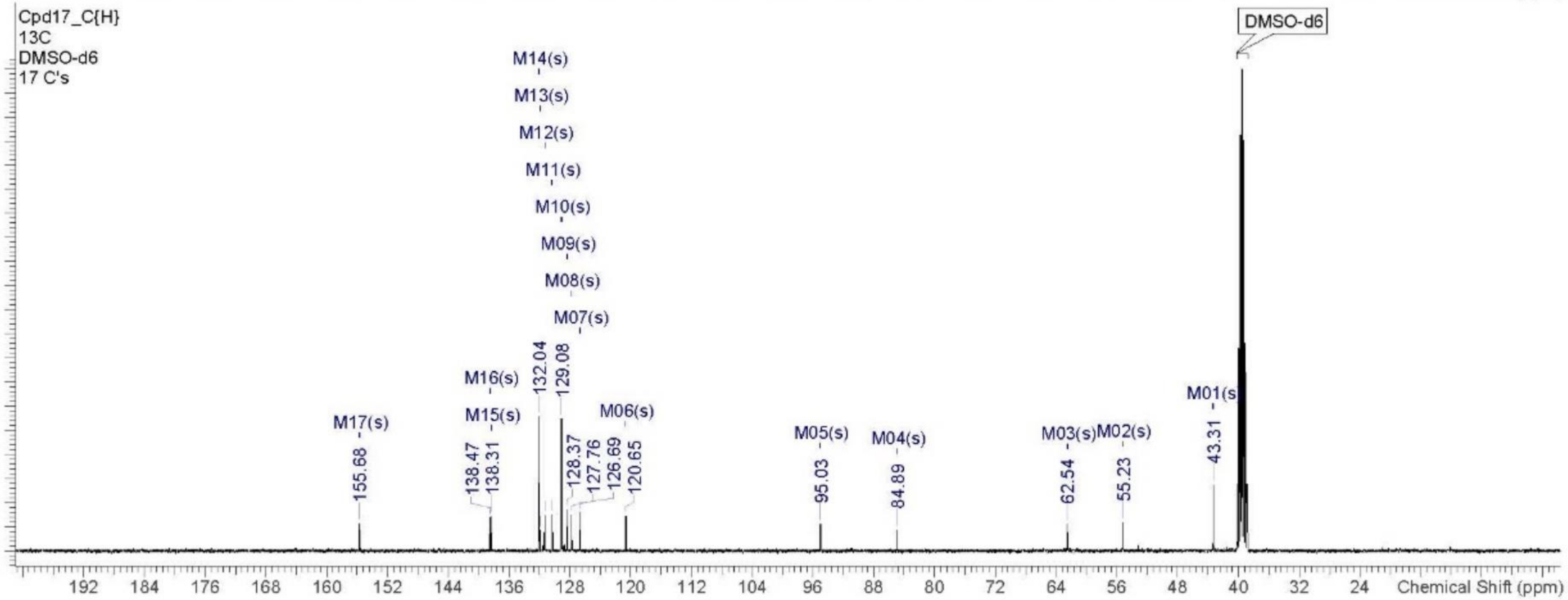
Cpd17_C{H}
13C
DMSO-d6
17 C's
DMSO-d6
M14(s)
M13(s)
M12(s)
M11(s)
M10(s)
M09(s)
M08(s)
M07(s)
M16(s)
M15(s)
M17(s)
M06(s)
M05(s)
M04(s)
M03(s)
M02(s)
M01(s)
155.68
138.47
138.31
132.04
129.08
128.37
127.76
126.69
120.65
95.03
84.89
62.54
55.23
43.31
Chemical Shift (ppm)

Chemistry - maXis HPLC-ESI Accurate Mass Report

**Analysis Info**		Acquisition Date	23/01/2020 09:21:23
Analysis Name	D:\Data\Chemistry\2020\Jan\MAM4-028_RC2_01_39788.d		
Method	soton lcms pos 120 to 1500.m	Operator	MSWEB@SOTON.AC.UK
Sample Name	MAM4-028	Instrument / Ser#	maXis 17
Comment	Analyst: JMH		

Acquisition Parameter

Source Type	ESI	Ion Polarity	Positive	Set Nebulizer	2.0 Bar
Focus	Not active	Set Capillary	4000 V	Set Dry Heater	230 °C
Scan Begin	120 m/z	Set End Plate Offset	-500 V	Set Dry Gas	6.0 l/min
Scan End	1500 m/z	Set Collision Cell RF	300.0 Vpp	Set Divert Valve	Waste

Cmpd 1, 2.2 min

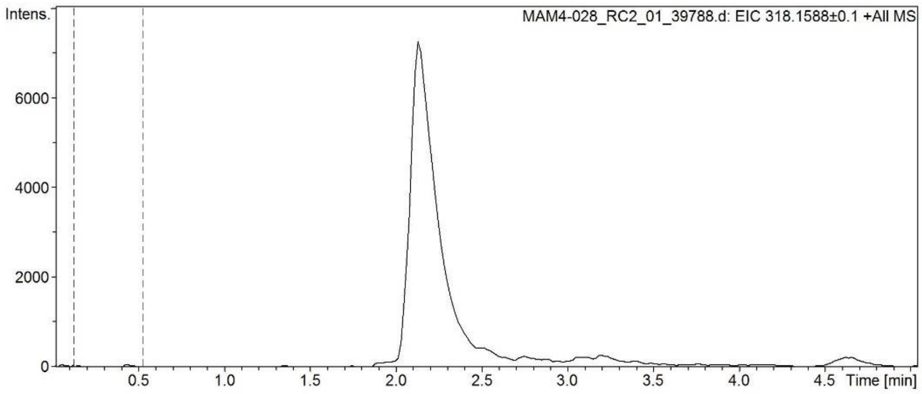

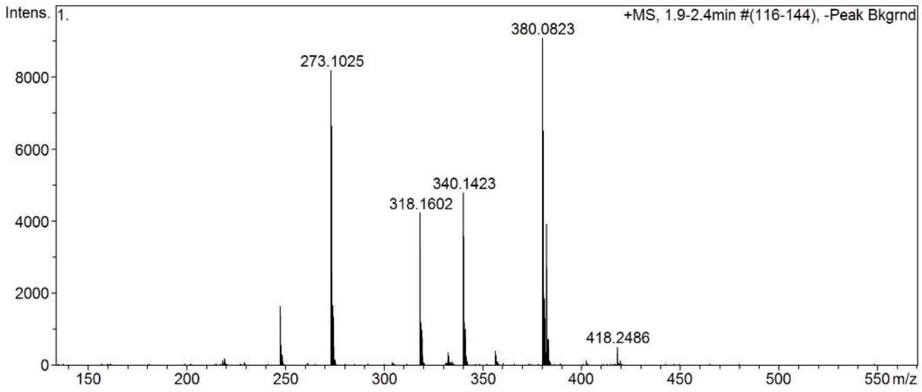

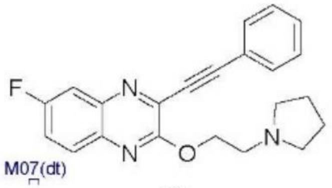
F
N
N
O
N

18

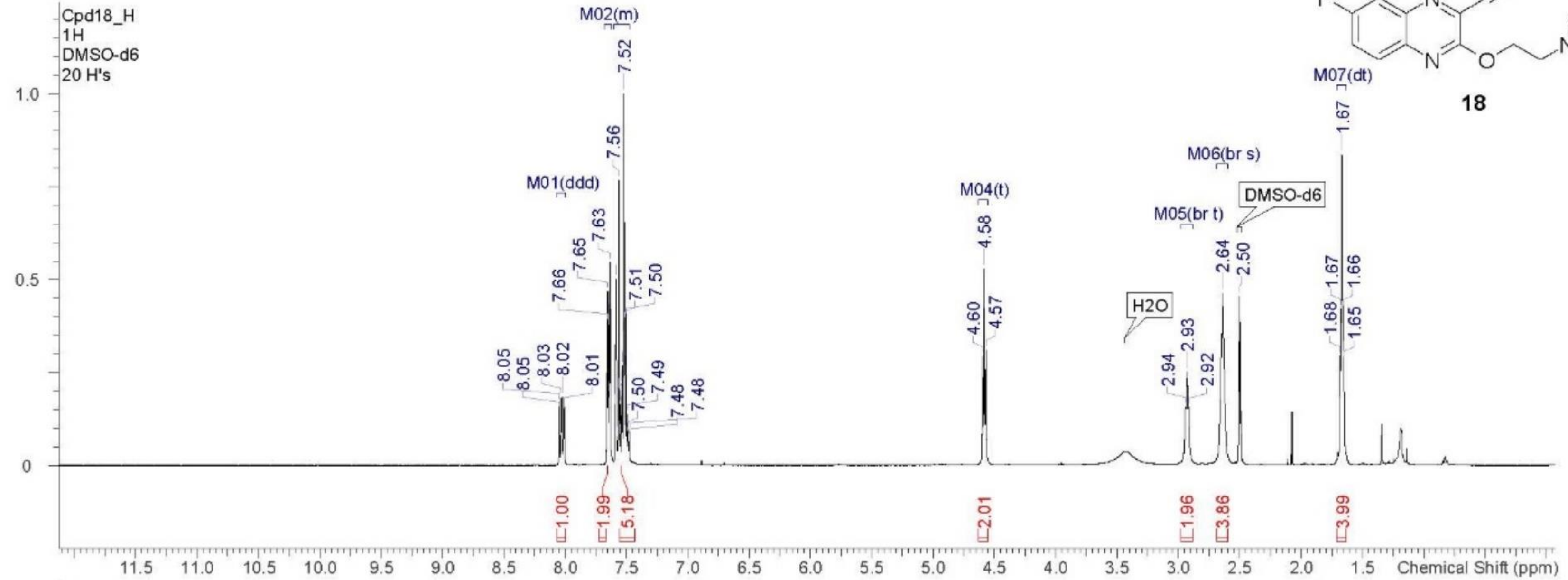
Cpd18_H
1H
DMSO-d6
20 H's
M01(ddd)
M02(m)
M04(t)
M05(br t)
M06(br s)
M07(dt)
DMSO-d6
H2O
8.05
8.05
8.03
8.02
8.01
7.66
7.65
7.63
7.56
7.52
7.51
7.50
7.50
7.49
7.48
7.48
4.60
4.58
4.57
2.94
2.93
2.92
2.64
2.50
1.68
1.67
1.67
1.66
1.65
1.00
1.99
5.18
2.01
1.96
3.86
3.99
1.0
0.5
0
11.5 11.0 10.5 10.0 9.5 9.0 8.5 8.0 7.5 7.0 6.5 6.0 5.5 5.0 4.5 4.0 3.5 3.0 2.5 2.0 1.5
Chemical Shift (ppm)

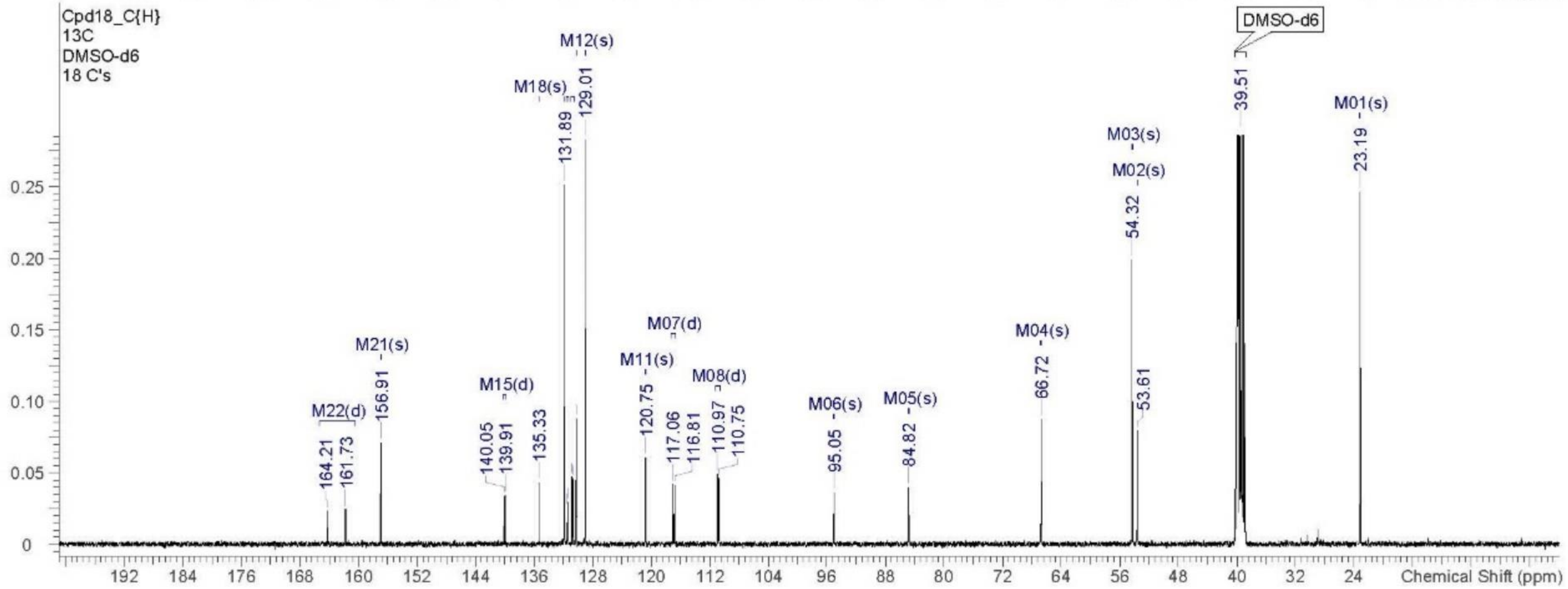
Cpd18_C{H}
13C
DMSO-d6
18 C's
M22(d)
M21(s)
M15(d)
M18(s)
M12(s)
M11(s)
M07(d)
M08(d)
M06(s)
M05(s)
M04(s)
M03(s)
M02(s)
M01(s)
DMSO-d6
164.21
161.73
156.91
140.05
139.91
135.33
131.89
129.01
120.75
117.06
116.81
110.97
110.75
95.05
84.82
66.72
54.32
53.61
39.51
23.19
0.25
0.20
0.15
0.10
0.05
0
192 184 176 168 160 152 144 136 128 120 112 104 96 88 80 72 64 56 48 40 32 24
Chemical Shift (ppm)

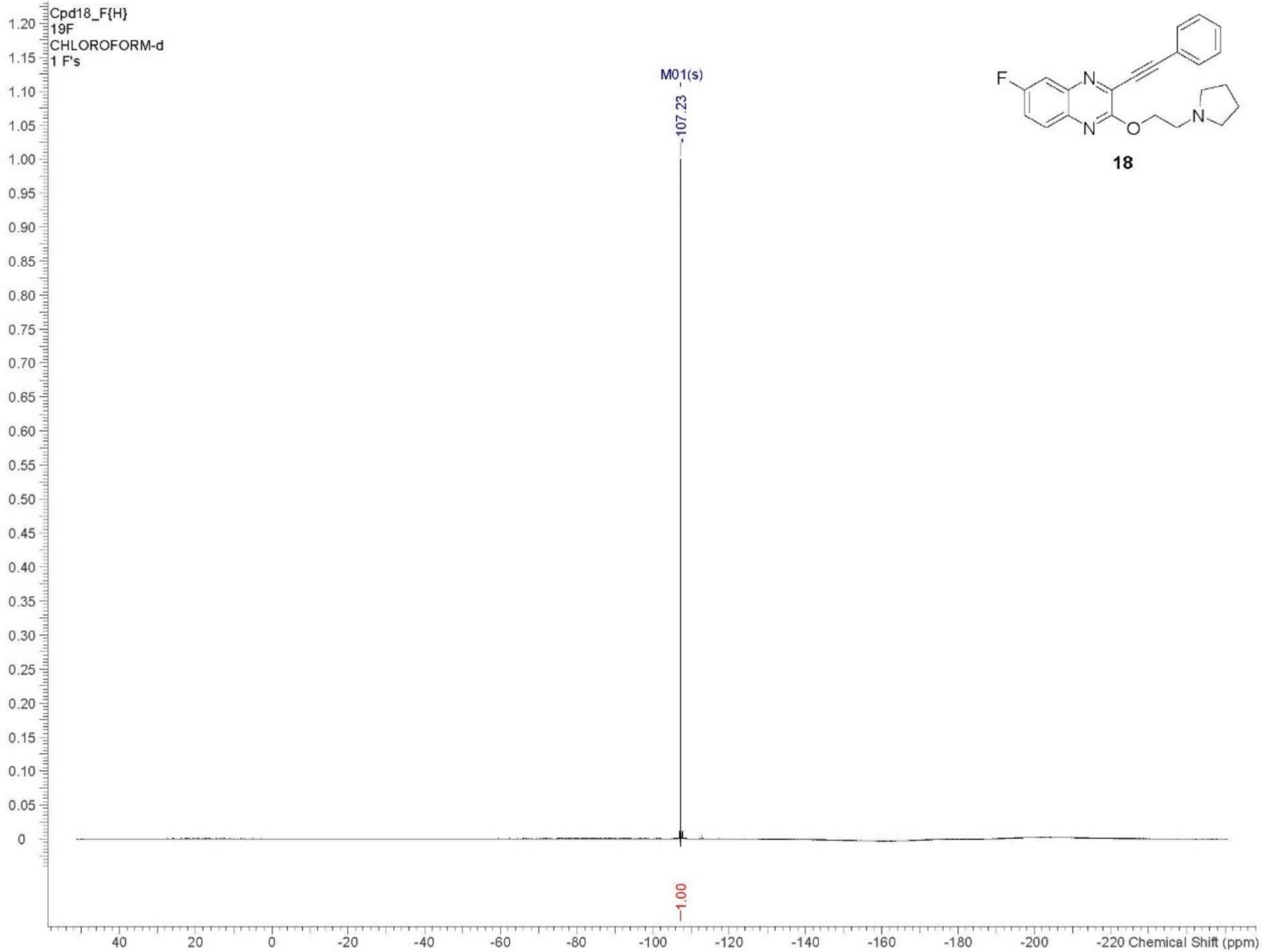
Cpd18_F{H}
19F
CHLOROFORM-d
1 F's
M01(s)
-107.23
1.00
F
N
N
O
N
18
Chemical Shift (ppm)

Top 5 Peak Report - UV

Sample ID: MAM7-188 LRMS
Group: Baud, M
Acquisition Date: 25/08/2021 13:36:25
Experiment: BLUE ESIPOSNEG C18 5 min
Filename: MAM7_188_LRMS_Michael_McCoy__Baud_M__99601.pdf

Submitter: Michael McCoy
Project: RP LC C18 custom
Instrument: Blue RP UHPLC-MS (B30:1023)

Absorbance, NL 7.907E05

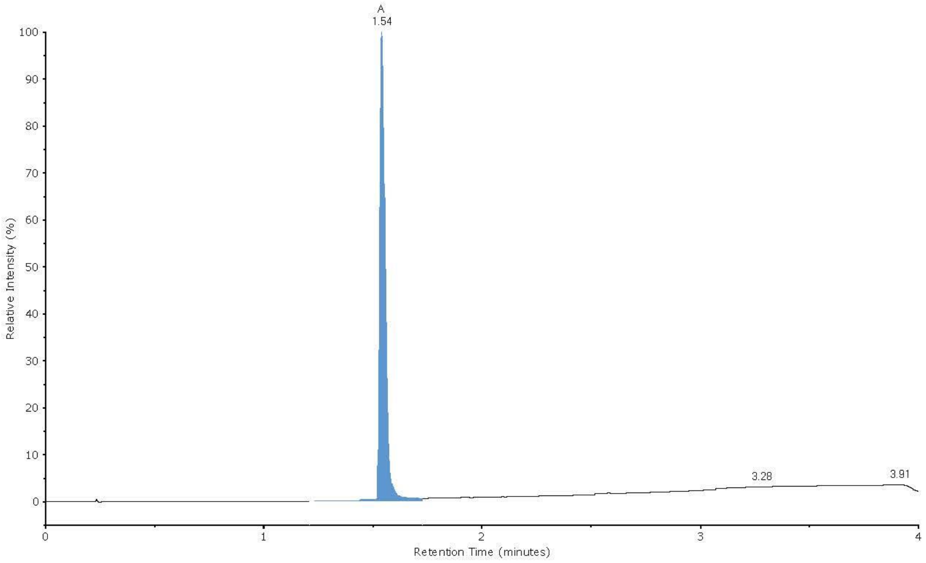

	RT Mins	Height	Height %	Area	Absolute Area %	Relative Area %
A	1.54	787886	100.00	1465291	100.00	100.00

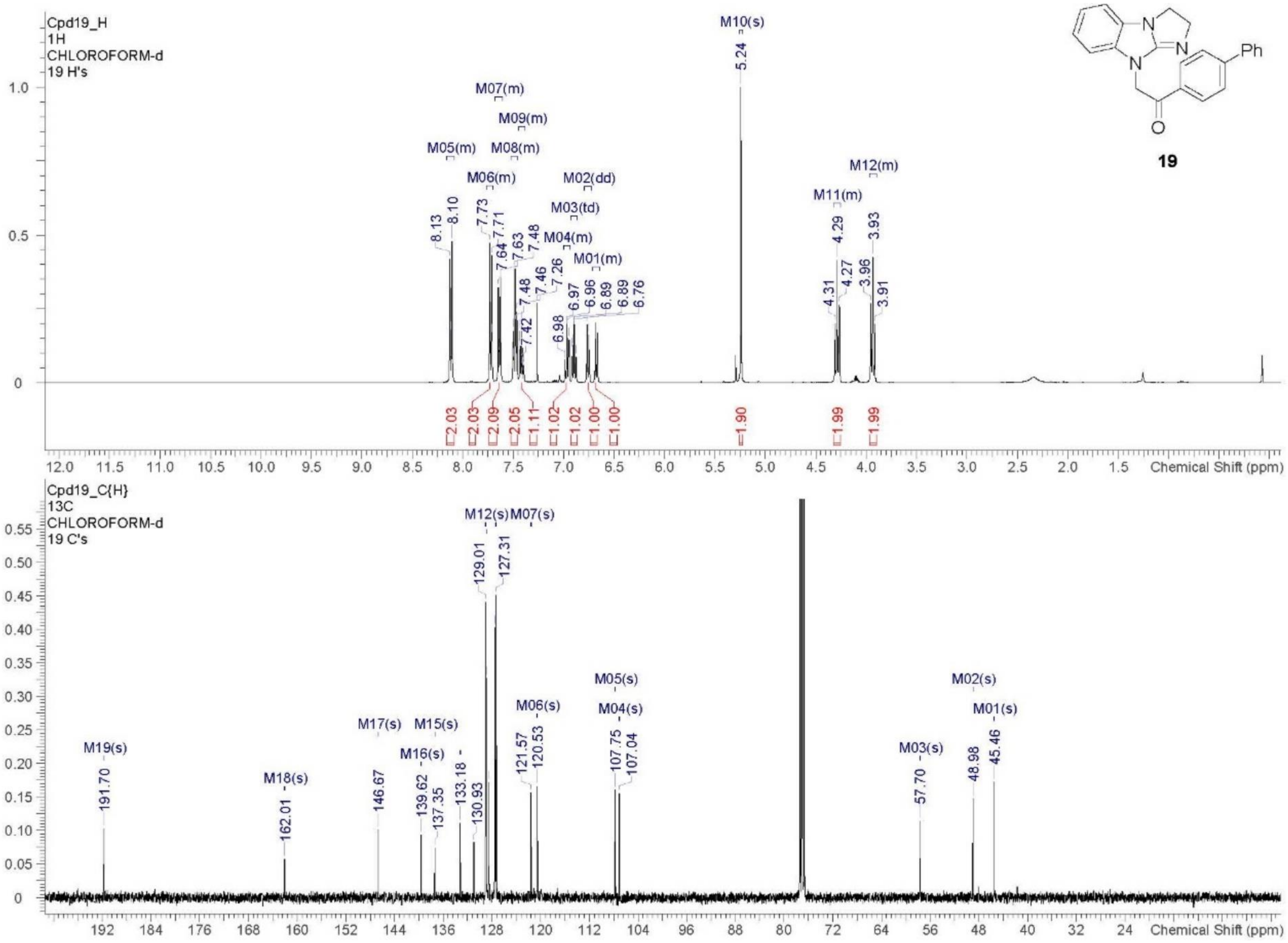
Cpd19_H
1H
CHLOROFORM-d
19 H's
M10(s)
5.24
M05(m) M06(m) M07(m) M08(m) M09(m)
M02(dd) M03(td) M04(m) M01(m)
M11(m) M12(m)
8.13 8.10 7.73 7.71 7.64 7.63 7.48 7.48 7.46 7.42 7.26 6.98 6.97 6.96 6.89 6.89 6.76
4.31 4.29 4.27 3.96 3.93 3.91
2.03 2.03 2.09 2.05 1.11 1.02 1.02 1.00 1.00 1.90 1.99 1.99
Chemical Shift (ppm)
Ph
19
Cpd19_C{H}
13C
CHLOROFORM-d
19 C's
M12(s) M07(s)
129.01 127.31
M19(s) 191.70
M18(s) 162.01
M17(s) 146.67
M16(s) 139.62
M15(s) 137.35
133.18 130.93
M06(s) 121.57 120.53
M05(s) M04(s) 107.75 107.04
M03(s) 57.70
M02(s) 48.98
M01(s) 45.46
Chemical Shift (ppm)

Chemistry - maXis HPLC-ESI Accurate Mass Report

Analysis Info

Analysis Name	D:\Data\Chemistry\2020\Jan\MAM4-018_RD1_01_39777.d	Acquisition Date	22/01/2020 18:01:09
Method	soton lcms pos 120 to 1500.m	Operator	MSWEB@SOTON.AC.UK
Sample Name	MAM4-018	Instrument / Ser#	maXis 17
Comment	Analyst: JMH		

Acquisition Parameter

Source Type	ESI	Ion Polarity	Positive	Set Nebulizer	2.0 Bar
Focus	Not active	Set Capillary	4000 V	Set Dry Heater	230 °C
Scan Begin	120 m/z	Set End Plate Offset	-500 V	Set Dry Gas	6.0 l/min
Scan End	1500 m/z	Set Collision Cell RF	300.0 Vpp	Set Divert Valve	Waste

+MS, 2.2-2.2min #(130-131)

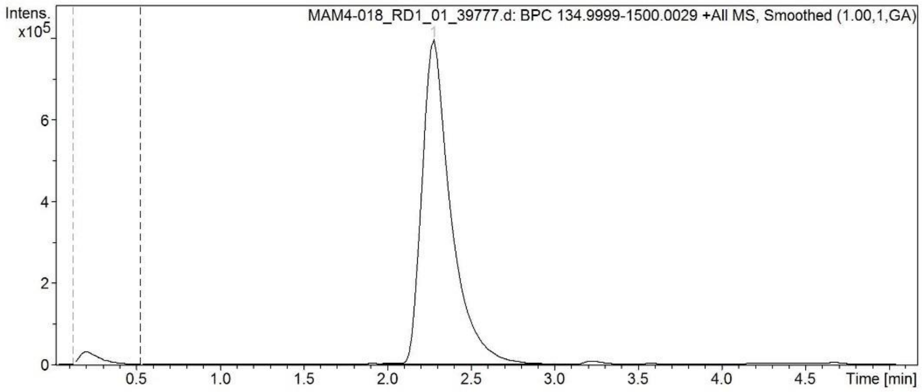

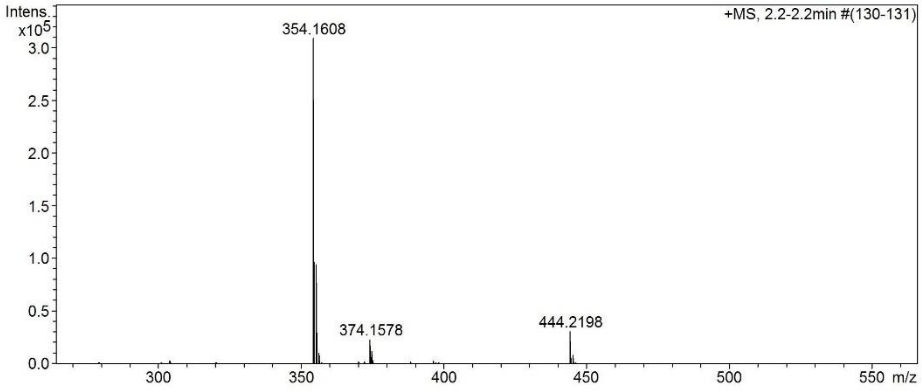

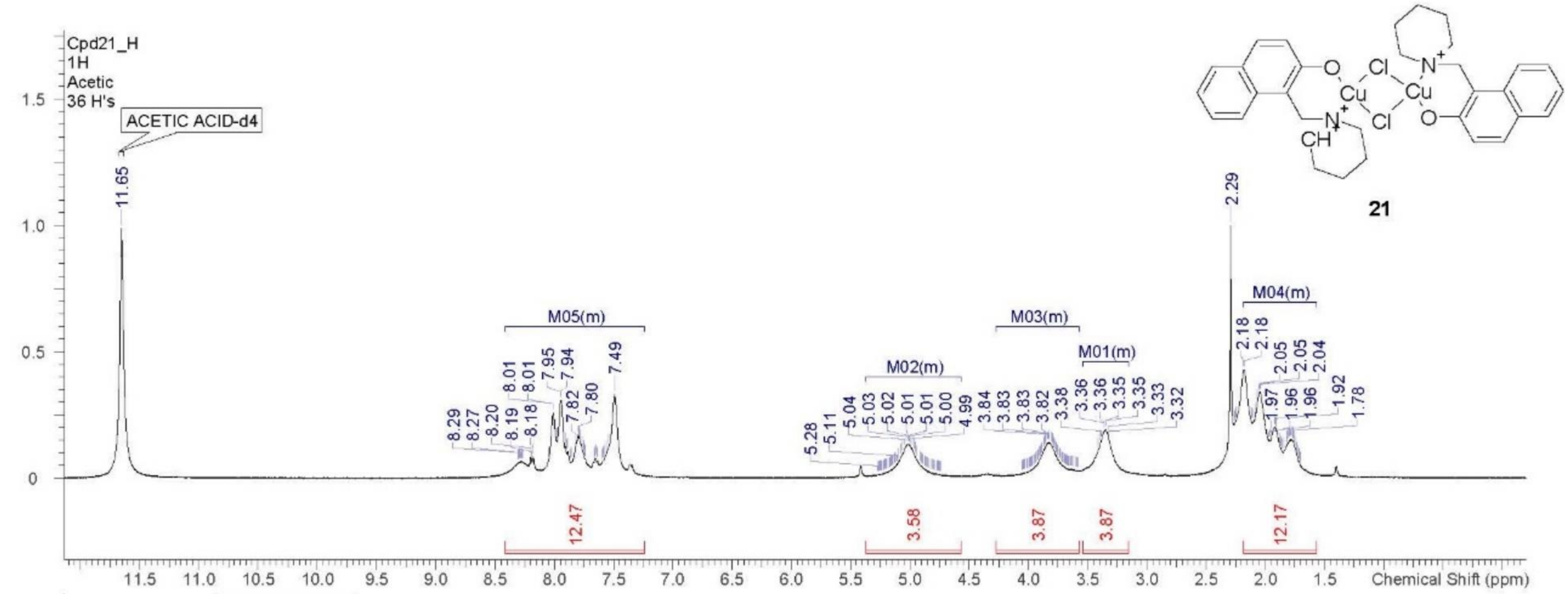
Cpd21_H
1H
Acetic
36 H's
ACETIC ACID-d4
11.65
M05(m)
8.29
8.27
8.20
8.19
8.18
8.01
8.01
7.95
7.94
7.82
7.80
7.49
M02(m)
5.28
5.11
5.04
5.03
5.02
5.01
5.01
5.00
4.99
M03(m)
3.84
3.83
3.83
3.82
3.38
M01(m)
3.36
3.36
3.35
3.35
3.33
3.32
2.29
M04(m)
2.18
2.18
2.05
2.05
2.04
1.97
1.96
1.96
1.92
1.78
12.47
3.58
3.87
3.87
12.17
Chemical Shift (ppm)
O
Cl
Cu
Cu
N+
N+
CH
Cl
O
21

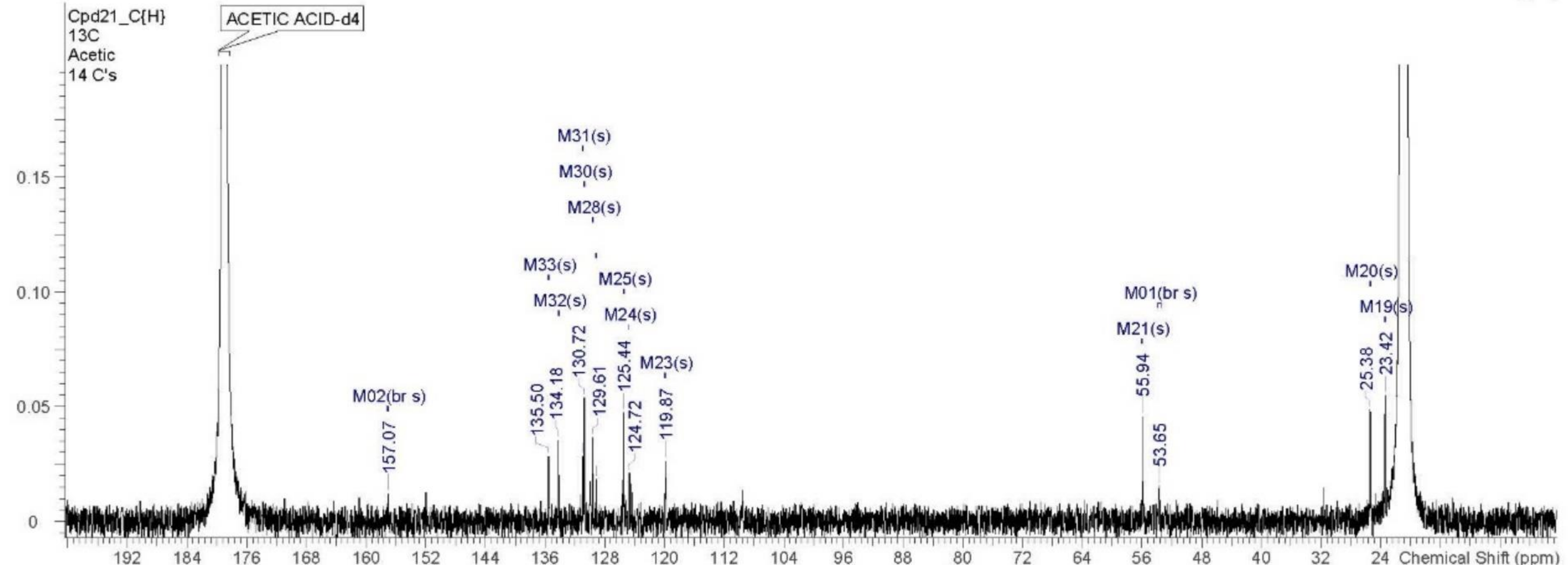
Cpd21_C{H}
13C
Acetic
14 C's
ACETIC ACID-d4
M02(br s)
157.07
M33(s)
M32(s)
135.50
134.18
M31(s)
M30(s)
M28(s)
130.72
129.61
M25(s)
M24(s)
125.44
124.72
M23(s)
119.87
M01(br s)
M21(s)
55.94
53.65
M20(s)
M19(s)
25.38
23.42
Chemical Shift (ppm)

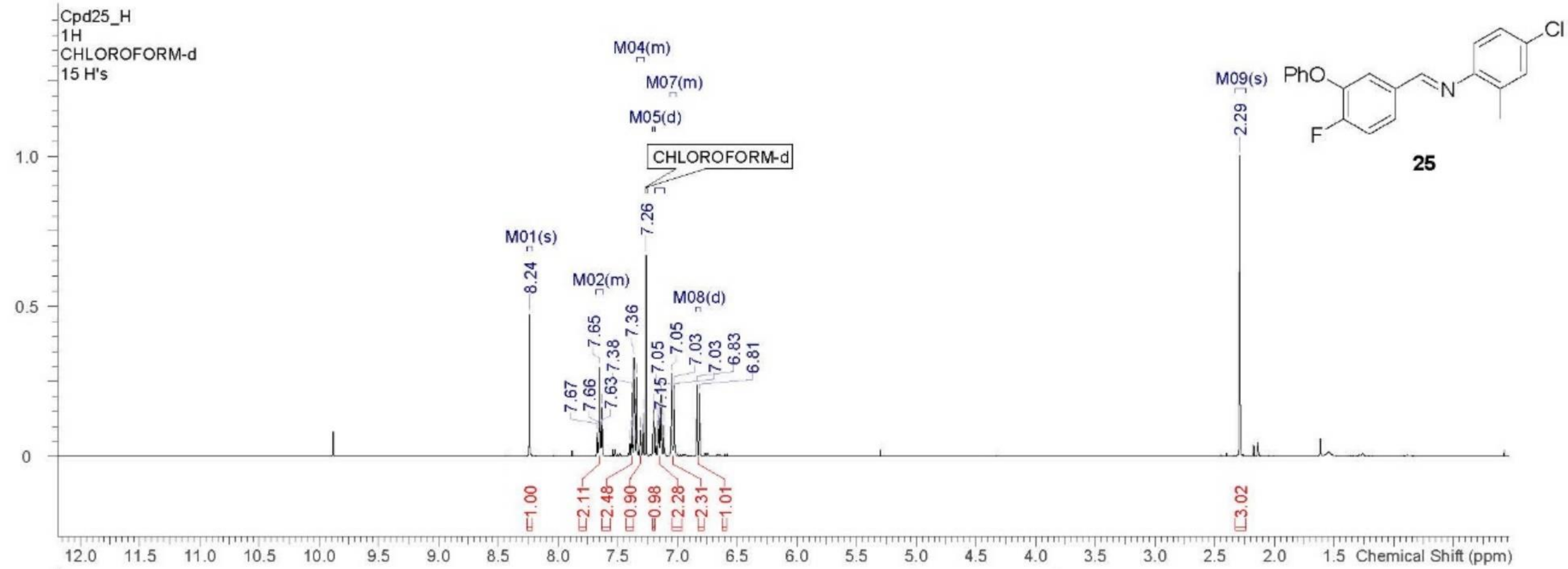
Cpd25_H
1H
CHLOROFORM-d
15 H's
M01(s)
8.24
M02(m)
7.67
7.66
7.65
7.63
7.38
7.36
M04(m)
M05(d)
M07(m)
7.15
7.05
7.05
7.03
7.03
M08(d)
6.83
6.81
CHLOROFORM-d
7.26
M09(s)
2.29
1.00
2.11
2.48
0.90
0.98
2.28
2.31
1.01
3.02
PhO
N
Cl
F
25
Chemical Shift (ppm)

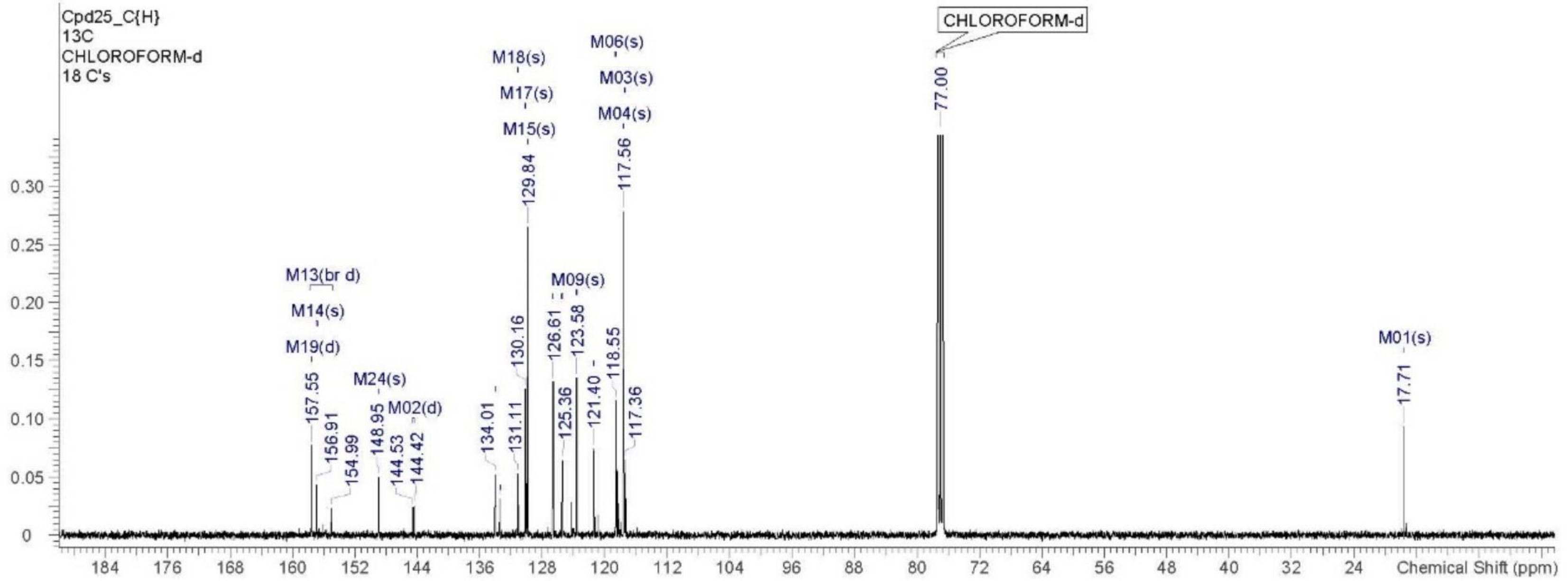
Cpd25_C{H}
13C
CHLOROFORM-d
18 C's
M13(br d)
M14(s)
M19(d)
157.55
156.91
154.99
M24(s)
148.95
M02(d)
144.53
144.42
134.01
131.11
130.16
M18(s)
M17(s)
M15(s)
129.84
M09(s)
126.61
125.36
123.58
121.40
118.55
M06(s)
M03(s)
M04(s)
117.56
117.36
CHLOROFORM-d
77.00
M01(s)
17.71
Chemical Shift (ppm)

Top 5 Peak Report - UV

Sample ID: MAM3-058 LRMS
Group: Baud, M
Acquisition Date: 10/11/2021 15:40:47
Experiment: BLUE ESIPOSNEG C18 5 min
Filename: MAM3_058_LRMS_Michael_McCoy__Baud_M__102762.pdf

Submitter: Michael McCoy
Project: RP LC C18 custom
Instrument: Blue RP UHPLC-MS (B30:1023)

Absorbance, NL 1.172E06

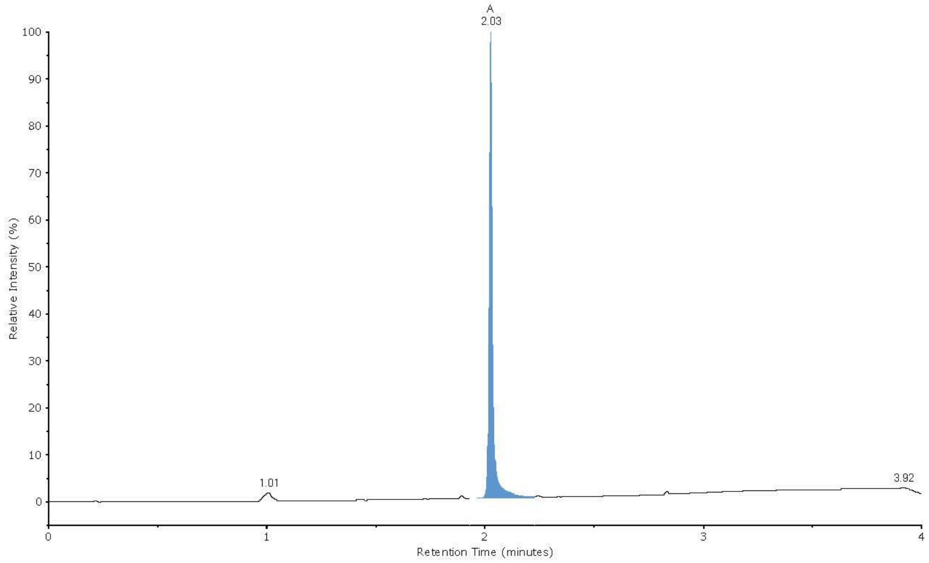

	RT Mins	Height	Height %	Area	Absolute Area %	Relative Area %
A	2.03	1162668	100.00	1331897	100.00	100.00

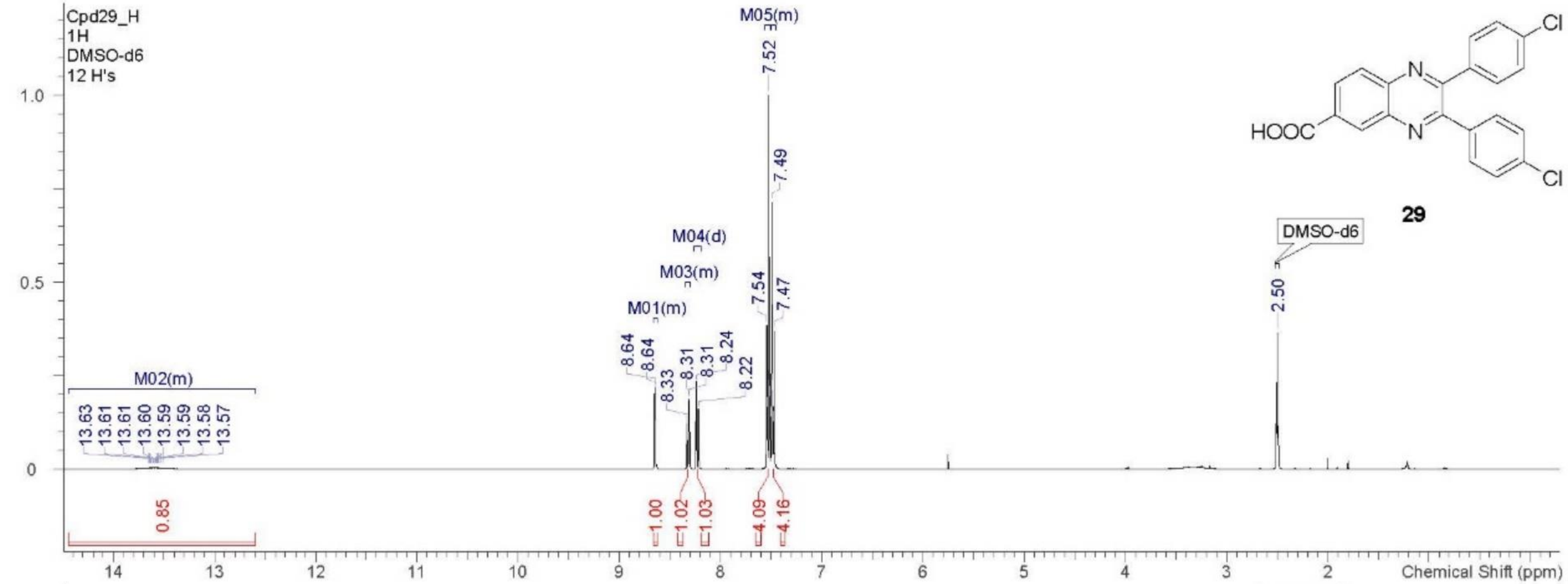
Cpd29_H
1H
DMSO-d6
12 H's
M05(m)
7.52
7.49
M04(d)
M03(m)
M01(m)
7.54
7.47
8.64
8.64
8.33
8.31
8.31
8.24
8.22
M02(m)
13.63
13.61
13.61
13.60
13.59
13.59
13.58
13.57
DMSO-d6
2.50
0.85
1.00
1.02
1.03
4.09
4.16
HOOC
N
N
Cl
Cl
29
Chemical Shift (ppm)

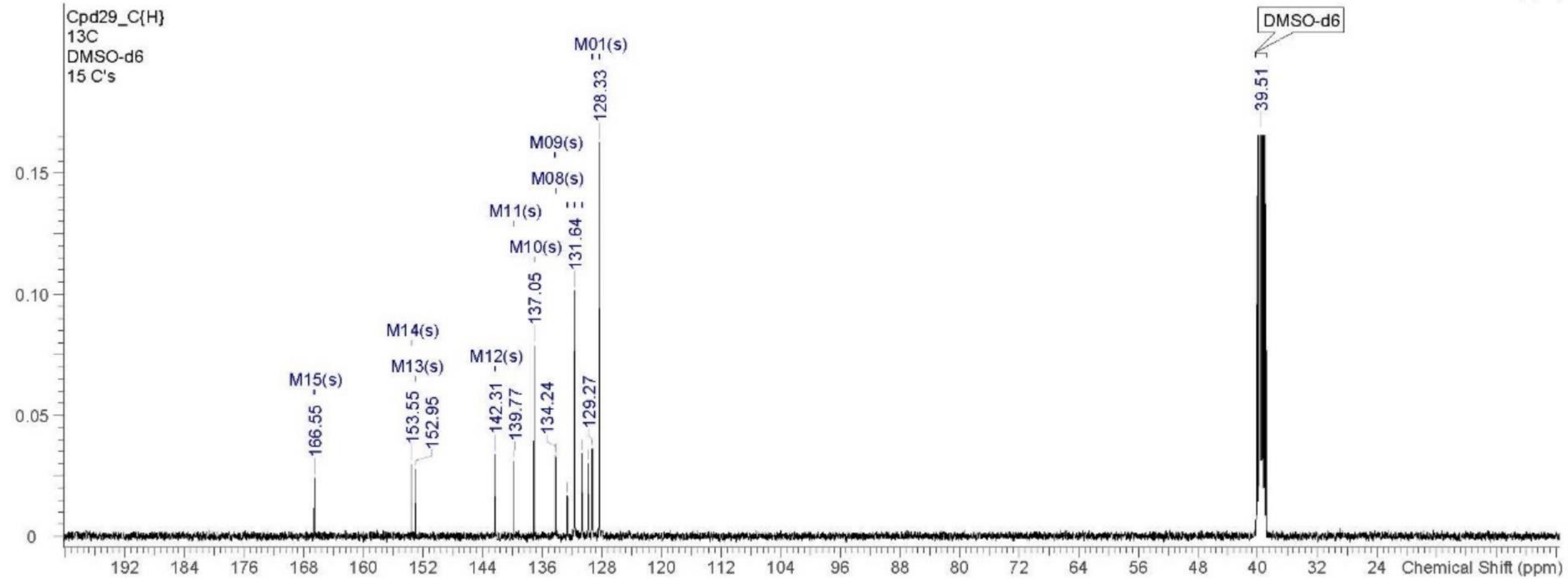
Cpd29_C{H}
13C
DMSO-d6
15 C's
M01(s)
128.33
DMSO-d6
39.51
M09(s)
M08(s)
M11(s)
M10(s)
131.64
137.05
M14(s)
M13(s)
M12(s)
M15(s)
166.55
153.55
152.95
142.31
139.77
134.24
129.27
Chemical Shift (ppm)

Chemistry - maXis HPLC-ESI Accurate Mass Report

Analysis Info

Analysis Name	D:\Data\Chemistry\2019\Dec\MAM4-011_RA6_01_39587.d	Acquisition Date	09/12/2019 16:03:15
Method	soton lcms pos 120 to 1500.m	Operator	MSWEB@SOTON.AC.UK
Sample Name	MAM4-011	Instrument / Ser#	maXis 17
Comment	Analyst: JMH		

Acquisition Parameter

Source Type	ESI	Ion Polarity	Positive	Set Nebulizer	2.0 Bar
Focus	Not active	Set Capillary	4000 V	Set Dry Heater	230 °C
Scan Begin	120 m/z	Set End Plate Offset	-500 V	Set Dry Gas	6.0 l/min
Scan End	1500 m/z	Set Collision Cell RF	300.0 Vpp	Set Divert Valve	Waste

Cmpd 2, 3.1 min

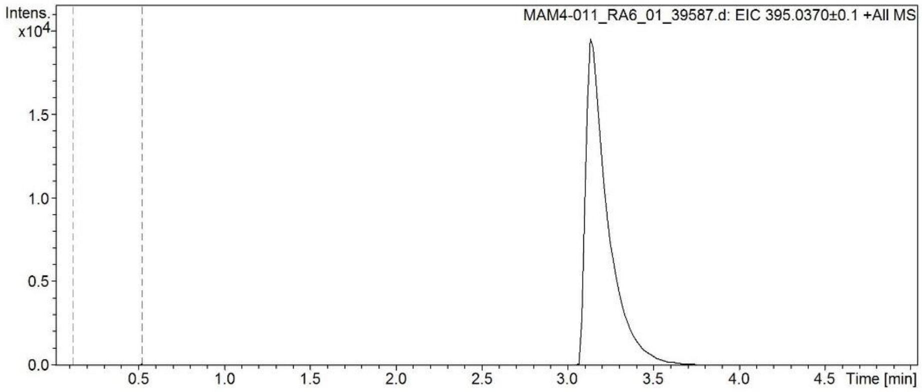

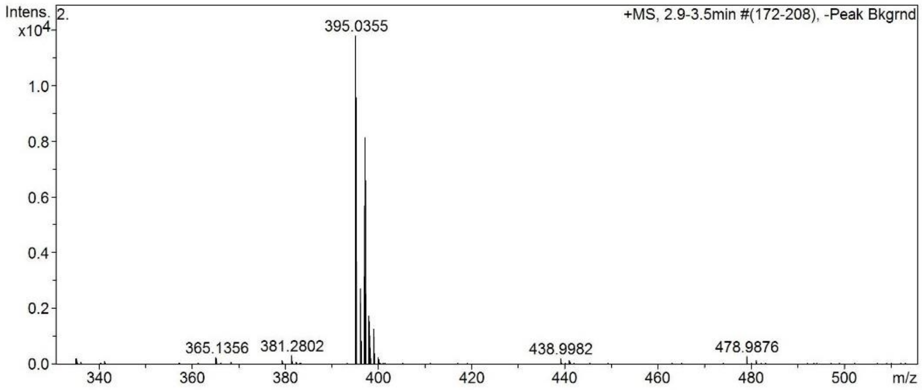

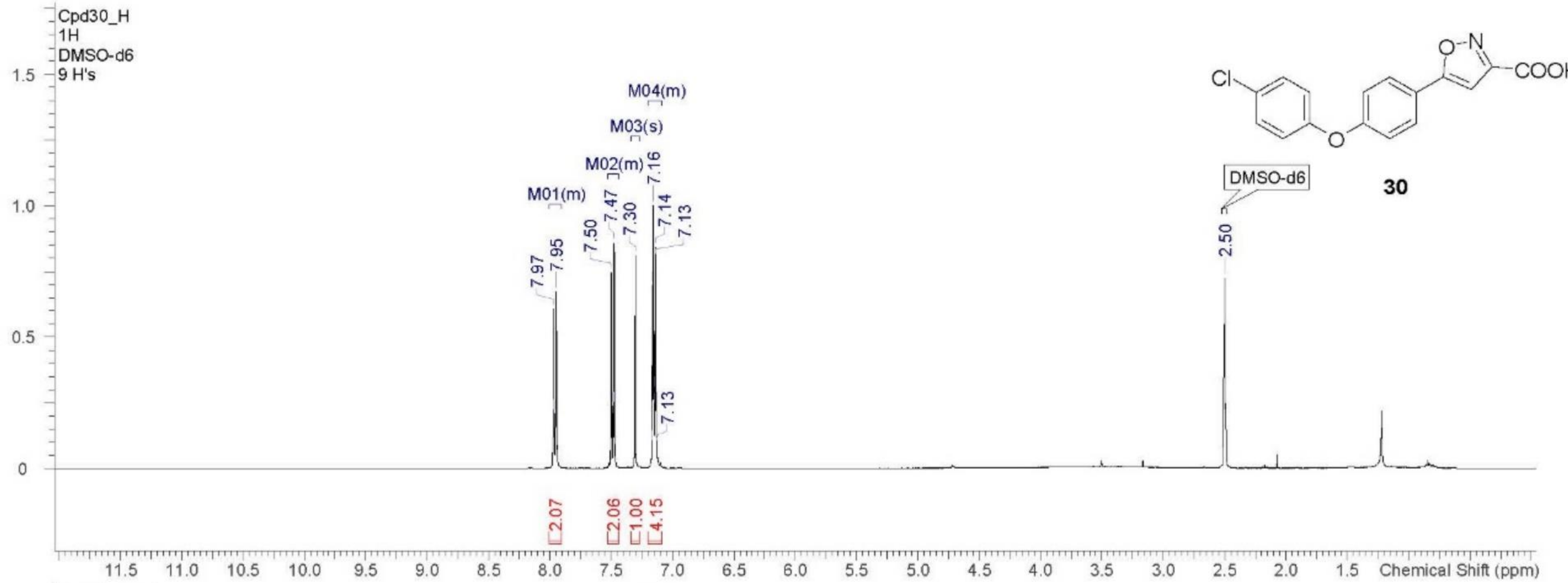
Cpd30_H
1H
DMSO-d6
9 H's
M01(m)
M02(m)
M03(s)
M04(m)
7.97
7.95
7.50
7.47
7.30
7.16
7.14
7.13
7.13
DMSO-d6
2.50
2.07
2.06
1.00
4.15
Cl
O
O
N
COOH
30
Chemical Shift (ppm)

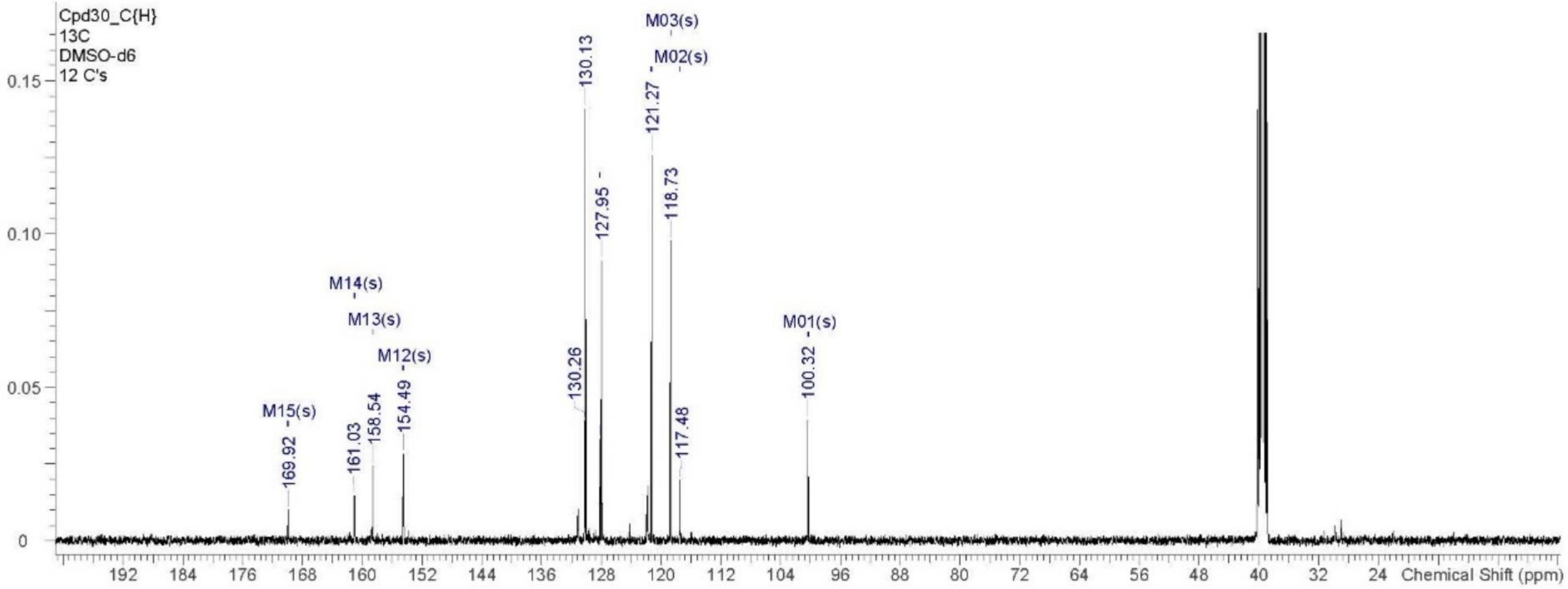
Cpd30_C{H}
13C
DMSO-d6
12 C's
M15(s)
M14(s)
M13(s)
M12(s)
M03(s)
M02(s)
M01(s)
169.92
161.03
158.54
154.49
130.26
130.13
127.95
121.27
118.73
117.48
100.32
Chemical Shift (ppm)

Top 5 Peak Report - UV

Sample ID: MAM4-138
Group: Baud, M
Acquisition Date: 09/09/2020 16:31:38
Experiment: BLUE ESIPOSNEG C18 5 min
Filename: MAM4_138_Michael_McCoy__Baud_M__82398.pdf

Submitter: Michael McCoy
Project: RP LC C18 custom
Instrument: Blue RP UHPLC-MS (B30:1023)

Absorbance, NL 2.249E06

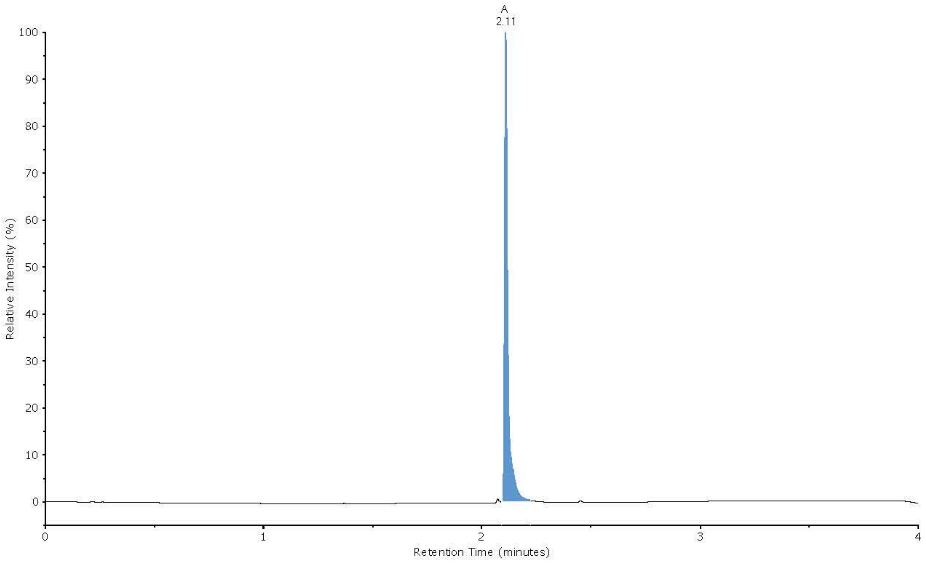

	RT Mins	Height	Height %	Area	Absolute Area %	Relative Area %
A	2.11	2247850	100.00	2479168	100.00	100.00

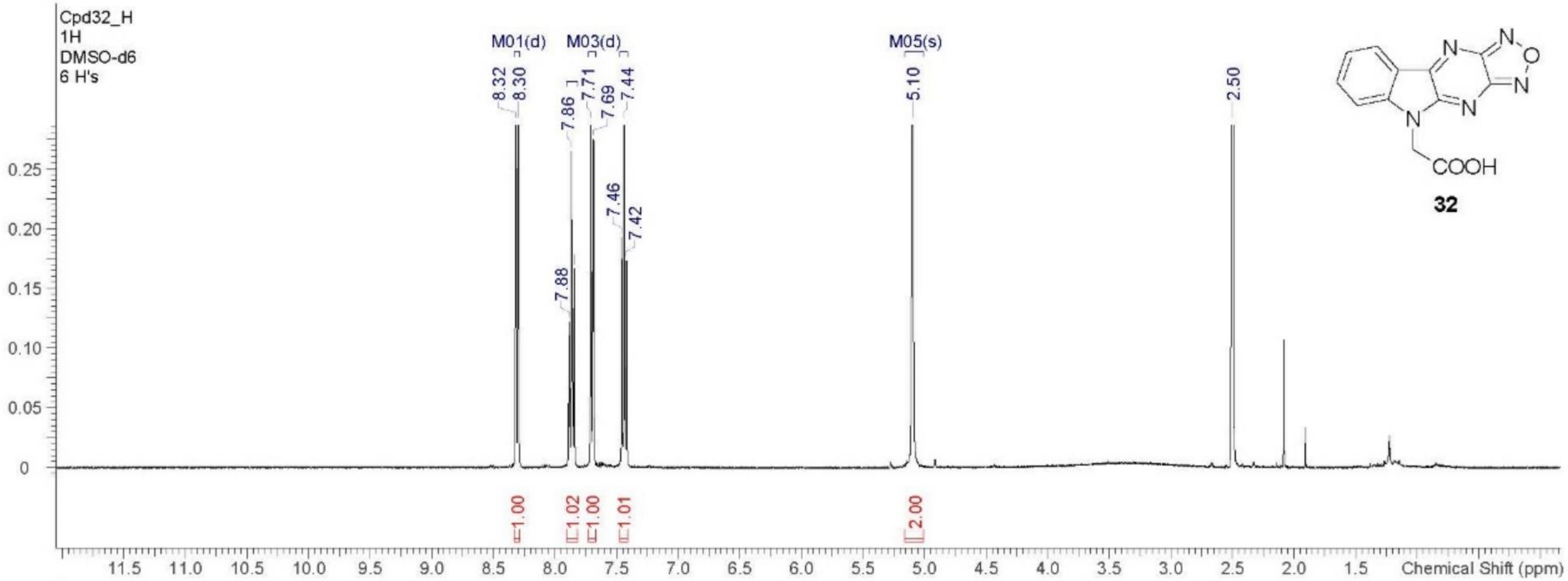
Cpd32_H
1H
DMSO-d6
6 H's
M01(d)
M03(d)
M05(s)
8.32
8.30
7.86
7.88
7.71
7.69
7.44
7.46
7.42
5.10
2.50
1.00
1.02
1.00
1.01
2.00
COOH
32
Chemical Shift (ppm)

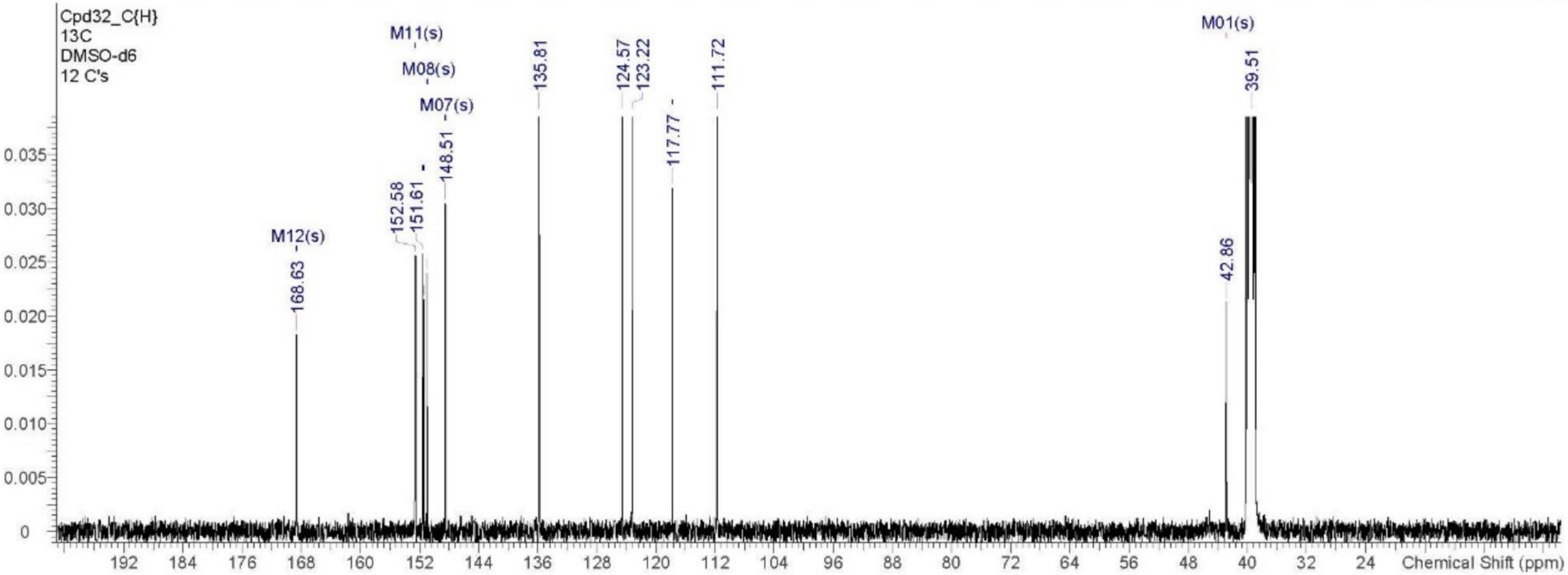
Cpd32_C{H}
13C
DMSO-d6
12 C's
M11(s)
M08(s)
M07(s)
M12(s)
M01(s)
168.63
152.58
151.61
148.51
135.81
124.57
123.22
117.77
111.72
42.86
39.51
Chemical Shift (ppm)

Top 5 Peak Report - UV

Sample ID: MAM7-167 LRMS
Group: Baud, M
Acquisition Date: 19/09/2021 13:23:38
Experiment: BLUE ESIPOSNEG C18 5 min
Filename: MAM7_167_LRMS_Michael_McCoy__Baud_M__100340.pdf

Submitter: Michael McCoy
Project: RP LC C18 custom
Instrument: Blue RP UHPLC-MS (B30:1023)

Absorbance, NL 6.898E05

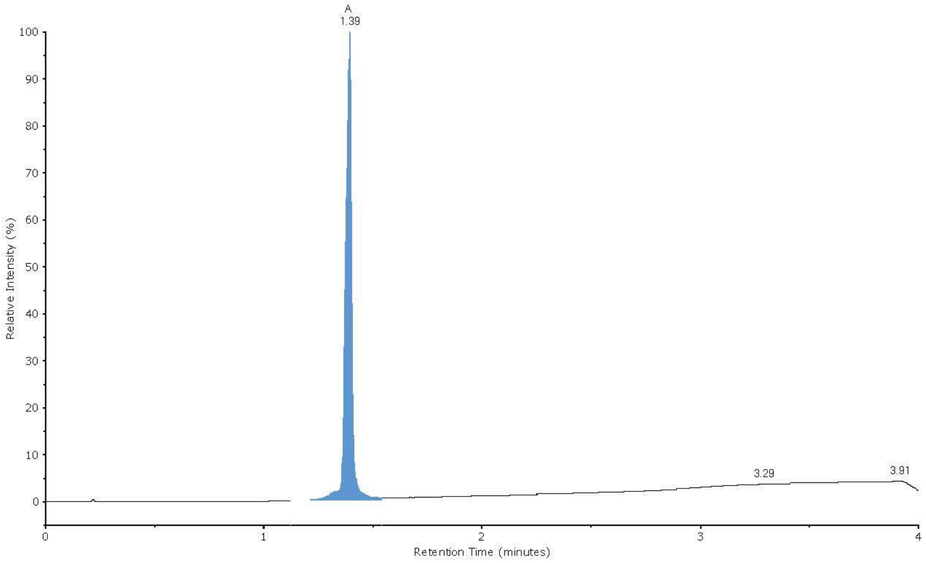

	RT Mins	Height	Height %	Area	Absolute Area %	Relative Area %
A	1.39	686711	100.00	1399158	100.00	100.00

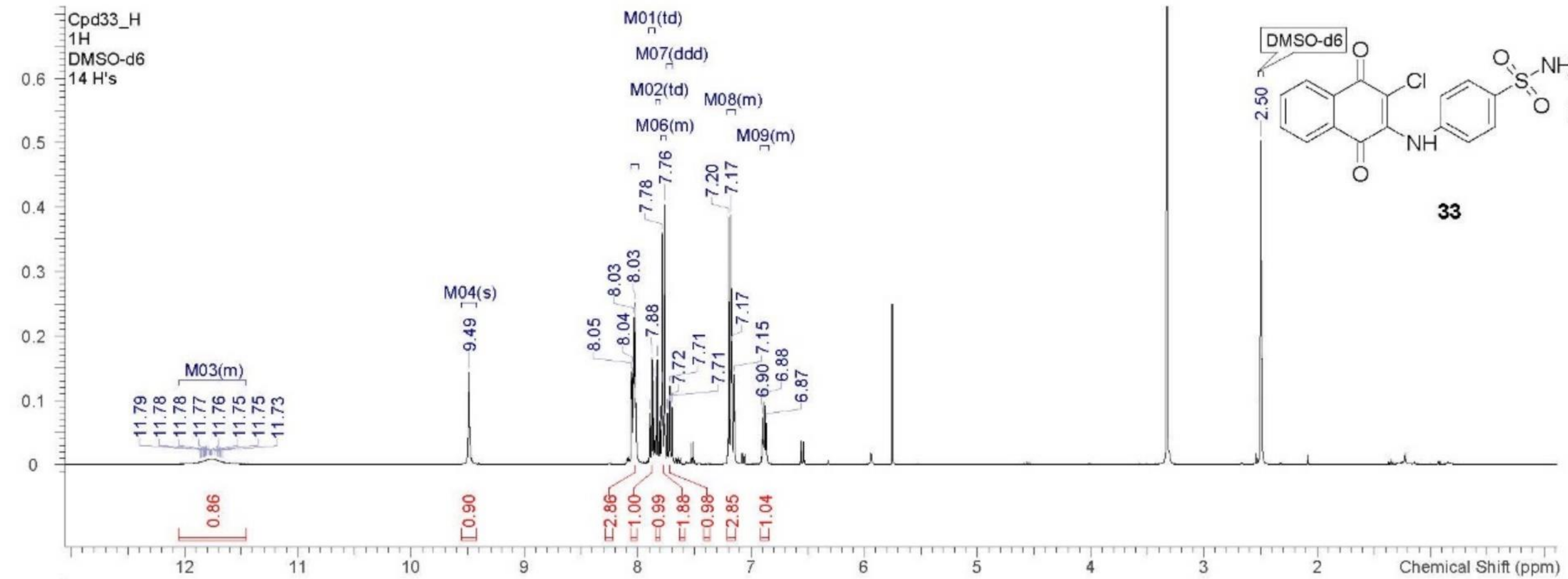
Cpd33_H
1H
DMSO-d6
14 H's
M01(td)
M07(ddd)
M02(td)
M06(m)
M08(m)
M09(m)
M04(s)
M03(m)
DMSO-d6
2.50
9.49
8.05
8.04
8.03
8.03
7.88
7.78
7.76
7.72
7.71
7.71
7.20
7.17
7.17
7.15
6.90
6.88
6.87
11.79
11.78
11.78
11.77
11.76
11.75
11.75
11.73
0.86
0.90
2.86
1.00
0.99
1.88
0.98
2.85
1.04
Cl
NH
O
S
N
33
Chemical Shift (ppm)

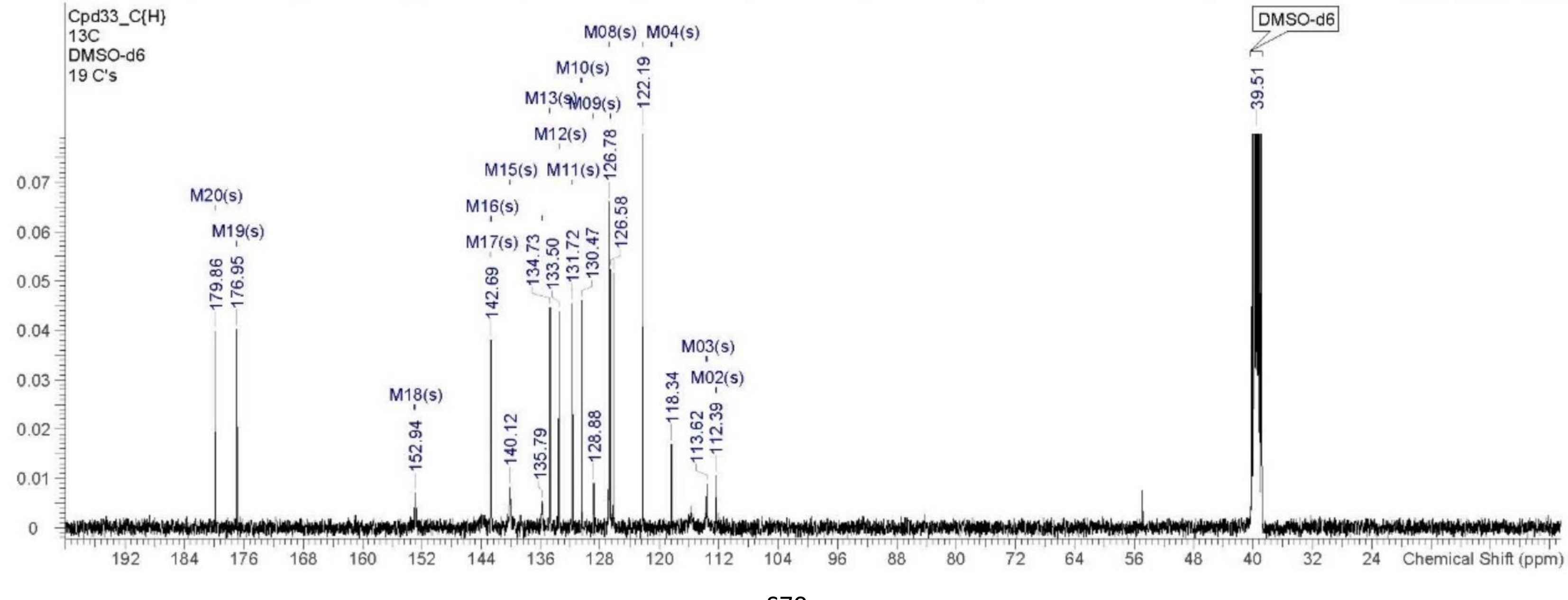
Cpd33_C{H}
13C
DMSO-d6
19 C's
M20(s)
M19(s)
M18(s)
M17(s)
M16(s)
M15(s)
M13(s)
M12(s)
M11(s)
M10(s)
M09(s)
M08(s)
M04(s)
M03(s)
M02(s)
DMSO-d6
39.51
179.86
176.95
152.94
142.69
140.12
135.79
134.73
133.50
131.72
130.47
128.88
126.78
126.58
122.19
118.34
113.62
112.39
Chemical Shift (ppm)

Chemistry - maXis HPLC-ESI Accurate Mass Report

Analysis Info

Analysis Name	D:\Data\Chemistry\2018\Dec\MAM2-DS-018_BB7_01_37962.d	Acquisition Date	12/12/2018 16:23:27
Method	soton lcms pos 120 to 1500.m	Operator	MSWEB@SOTON.AC.UK
Sample Name	MAM2-DS-018	Instrument / Ser#	maXis 17
Comment	Analyst: JMH		

Acquisition Parameter

Source Type	ESI	Ion Polarity	Positive	Set Nebulizer	2.0 Bar
Focus	Not active	Set Capillary	4000 V	Set Dry Heater	230 °C
Scan Begin	120 m/z	Set End Plate Offset	-500 V	Set Dry Gas	6.0 l/min
Scan End	1500 m/z	Set Collision Cell RF	300.0 Vpp	Set Divert Valve	Waste

+MS, 2.0-2.0min #(117-119)

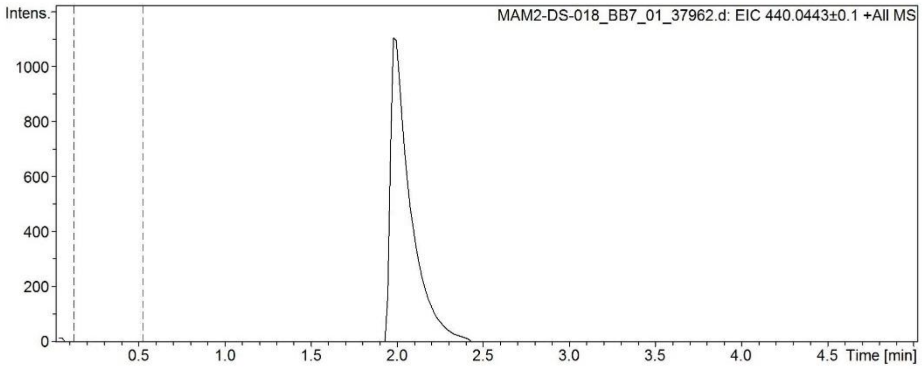

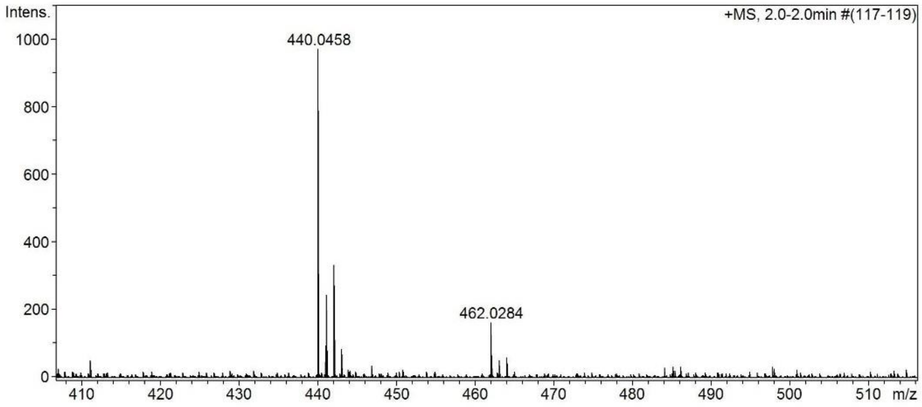

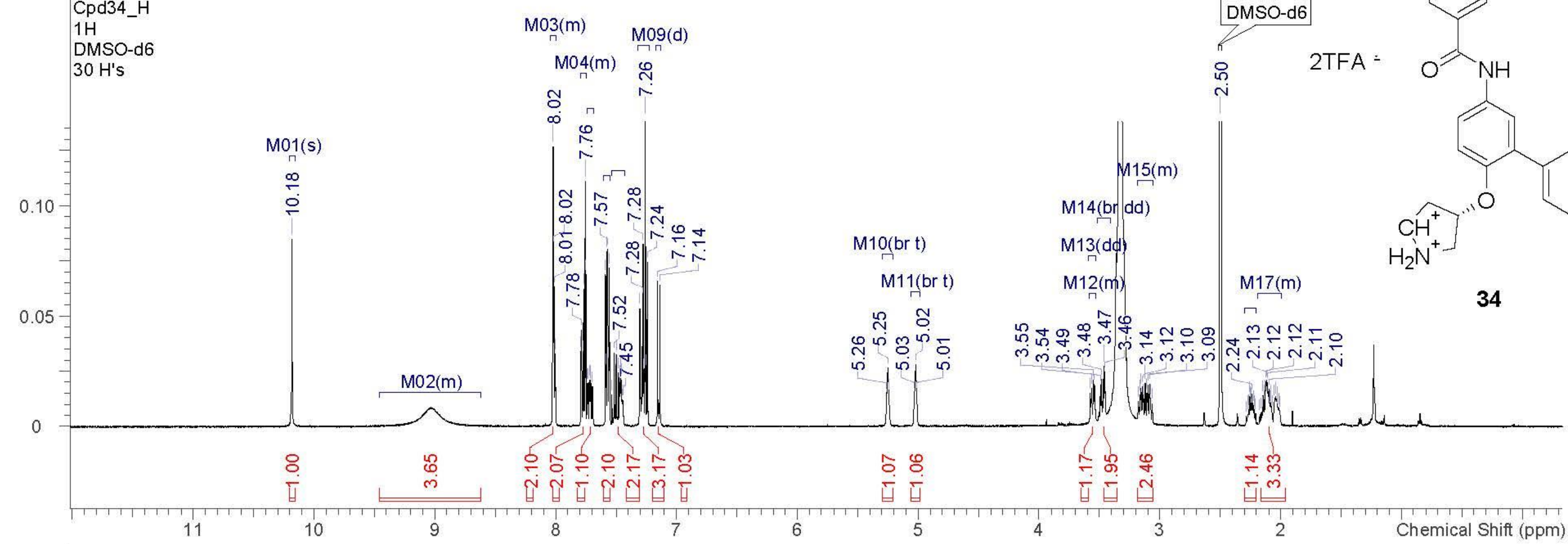
Cpd34_H
1H
DMSO-d6
30 H's
M01(s)
10.18
M02(m)
M03(m)
8.02
8.02
8.01
M04(m)
7.78
7.76
7.57
7.52
7.45
M09(d)
7.28
7.28
7.26
7.24
7.16
7.14
M10(br t)
5.26
5.25
5.03
M11(br t)
5.02
5.01
M12(m)
M13(dd)
M14(br dd)
M15(m)
3.55
3.54
3.49
3.48
3.47
3.46
3.14
3.12
3.10
3.09
DMSO-d6
2.50
M17(m)
2.24
2.13
2.12
2.12
2.11
2.10
0.10
0.05
0
1.00
3.65
2.10
2.07
1.10
2.10
2.17
3.17
1.03
1.07
1.06
1.17
1.95
2.46
1.14
3.33
11
10
9
8
7
6
5
4
3
2
Chemical Shift (ppm)

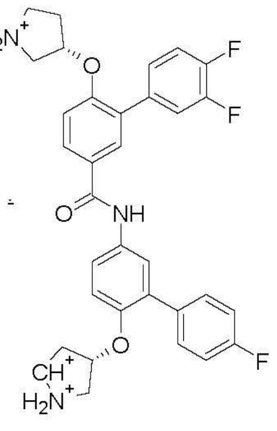
H2N+
O
F
F
2TFA
O
NH
O
F
CH+
H2N+

34

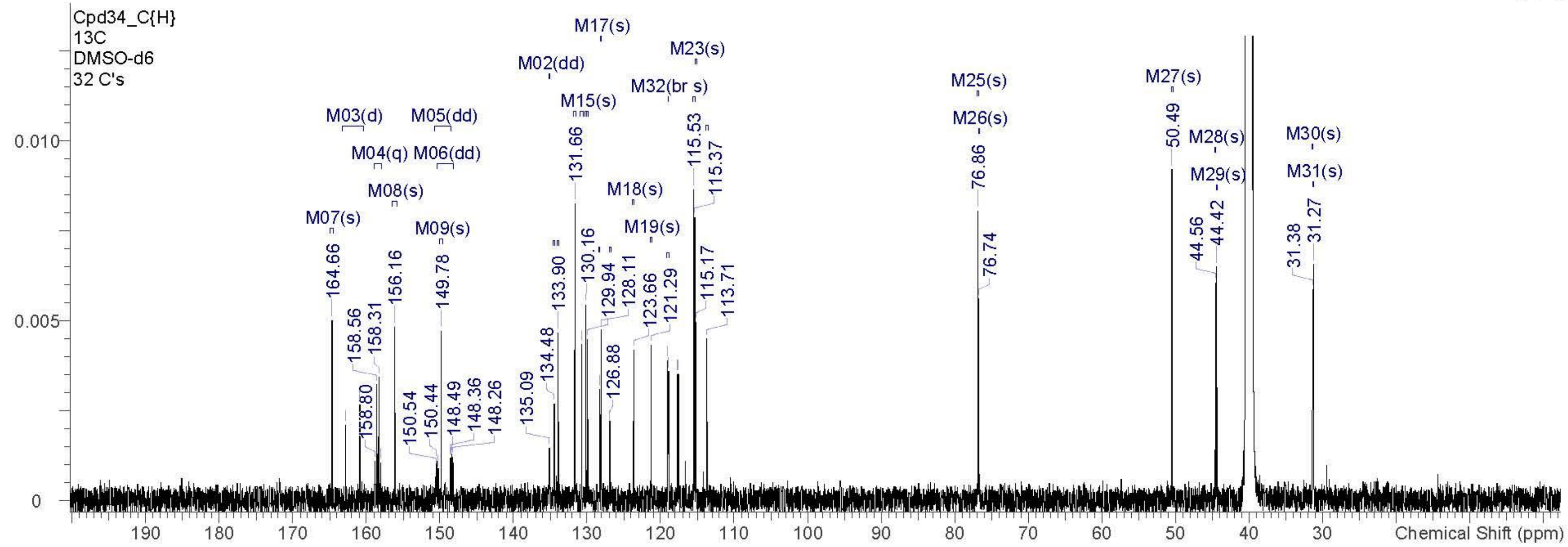
Cpd34_C{H}
13C
DMSO-d6
32 C's
M07(s)
164.66
M03(d)
158.80
158.56
158.31
M04(q)
M08(s)
156.16
M05(dd)
M06(dd)
150.54
150.44
M09(s)
149.78
148.49
148.36
148.26
M02(dd)
135.09
134.48
133.90
M15(s)
M17(s)
131.66
130.16
129.94
128.11
126.88
M18(s)
M19(s)
123.66
121.29
M32(br s)
M23(s)
115.53
115.37
115.17
113.71
M25(s)
M26(s)
76.86
76.74
M27(s)
50.49
M28(s)
M29(s)
44.56
44.42
M30(s)
M31(s)
31.38
31.27
0.010
0.005
0
190
180
170
160
150
140
130
120
110
100
90
80
70
60
50
40
30
Chemical Shift (ppm)

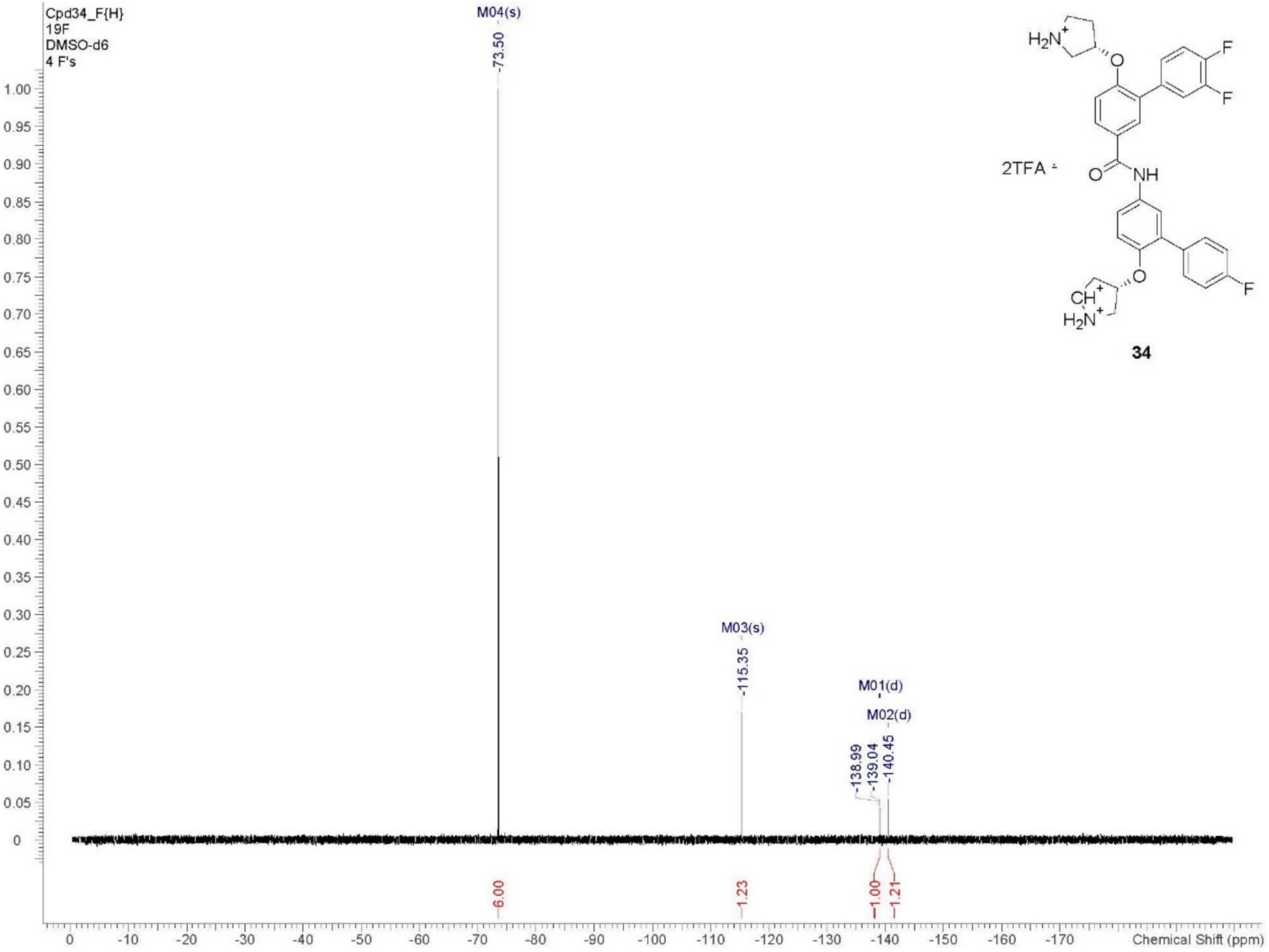

Cpd34_F{H}
19F
DMSO-d6
4 F's
M04(s)
-73.50
M03(s)
-115.35
M01(d)
M02(d)
-138.99
-139.04
-140.45
6.00
1.23
1.00
1.21
Chemical Shift (ppm)
2TFA
NH
O
F
34

Top 5 Peak Report - UV

Sample ID: MAM7-145 HPLC F1
Group: Baud, M
Acquisition Date: 18/05/2021 18:48:44
Experiment: BLUE ESIPOSNEG C18 5 min
Filename: MAM7_145_HPLC_F1_Michael_McCoy__Baud_M__94741.pdf

Submitter: Michael McCoy
Project: RP LC C18 custom
Instrument: Blue RP UHPLC-MS (B30:1023)

Absorbance, NL 1.958E05

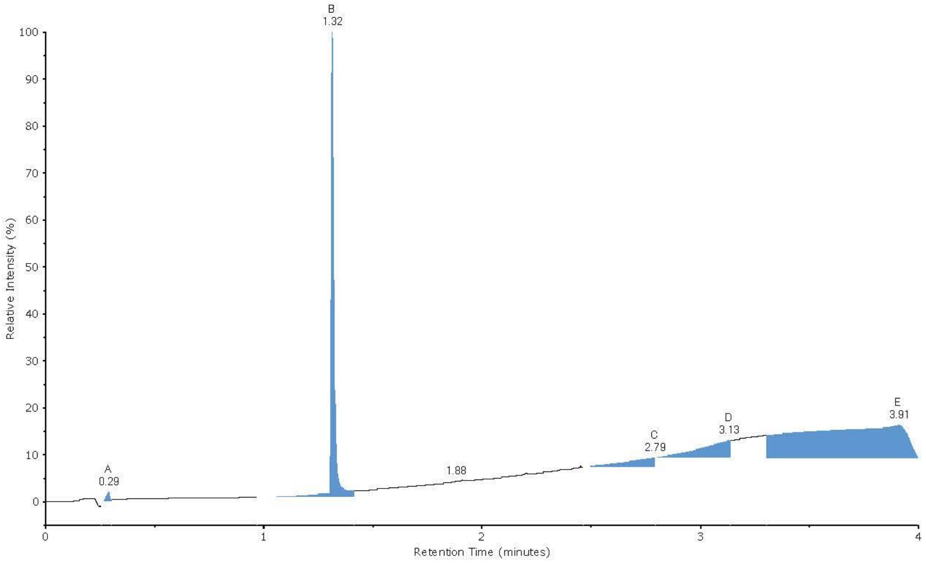

	RT Mins	Height	Height %	Area	Absolute Area %	Relative Area %
A	0.29	4509	2.34	9315	1.20	2.01
B	1.32	192763	100.00	203883	26.20	44.03
C	2.79	4142	2.15	37986	4.88	8.20
D	3.13	7165	3.72	63963	8.22	13.81
E	3.91	13737	7.13	463086	59.50	100.00

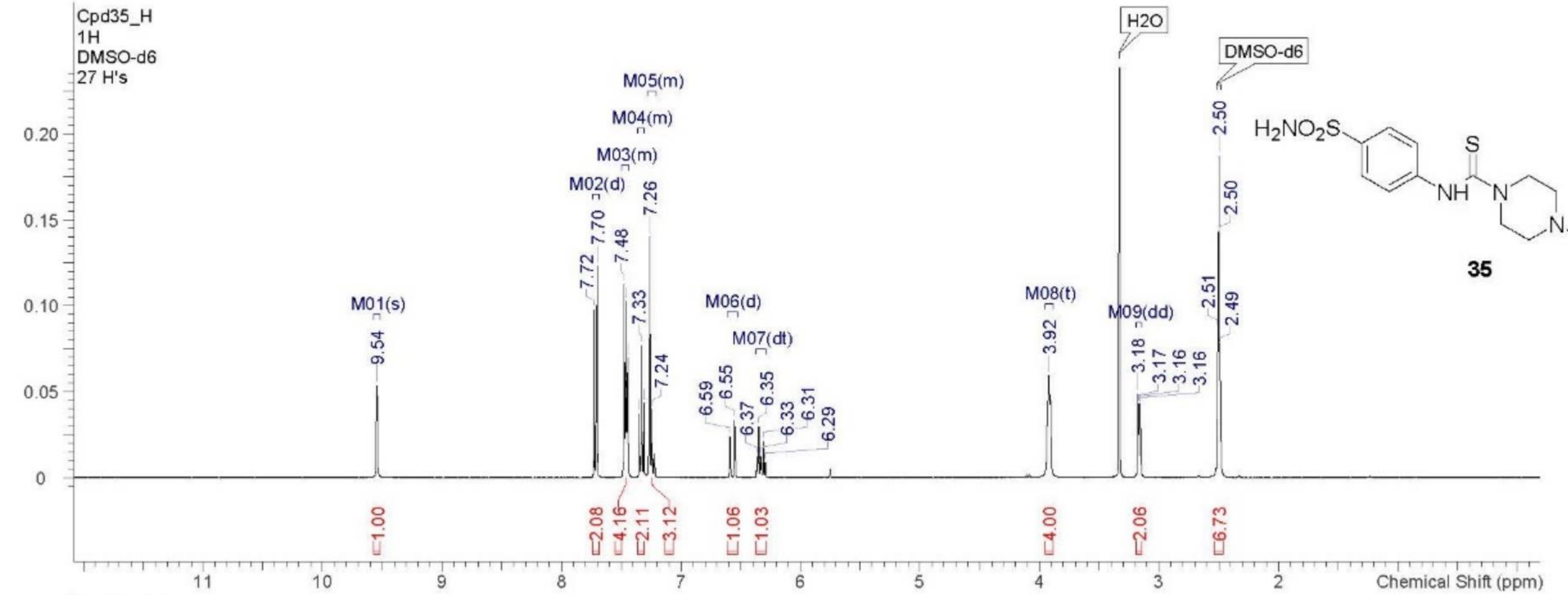
Cpd35_H
1H
DMSO-d6
27 H's
H2O
DMSO-d6
M01(s)
9.54
M02(d)
7.72
7.70
M03(m)
7.48
M04(m)
7.33
M05(m)
7.26
7.24
M06(d)
6.59
6.55
M07(dt)
6.37
6.35
6.33
6.31
6.29
M08(t)
3.92
M09(dd)
3.18
3.17
3.16
3.16
2.51
2.50
2.50
2.49
1.00
2.08
4.16
2.11
3.12
1.06
1.03
4.00
2.06
6.73
Chemical Shift (ppm)
H2NO2S
S
NH
N
N
Ph
35

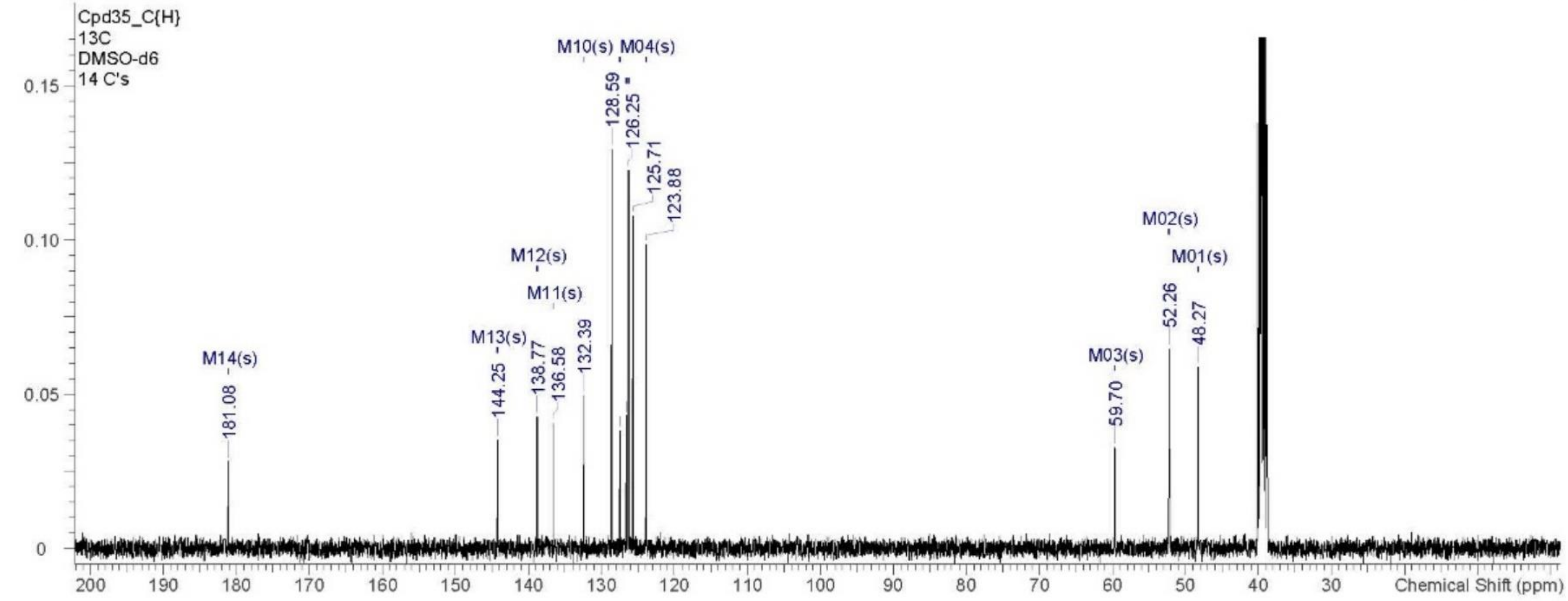
Cpd35_C{H}
13C
DMSO-d6
14 C's
M14(s)
181.08
M13(s)
144.25
M12(s)
138.77
M11(s)
136.58
132.39
M10(s)
128.59
M04(s)
126.25
125.71
123.88
M03(s)
59.70
M02(s)
52.26
M01(s)
48.27
Chemical Shift (ppm)

Chemistry - maXis HPLC-ESI Accurate Mass Report

**Analysis Info**		Acquisition Date	03/04/2019 16:26:57
Analysis Name	D:\Data\Chemistry\2019\Apr\MAM2-DS-010_GB2_01_38373.d		
Method	soton lcms pos 120 to 1500.m	Operator	MSWEB@SOTON.AC.UK
Sample Name	MAM2-DS-010	Instrument / Ser#	maXis 17
Comment	Analyst: JMH		

Acquisition Parameter

Source Type	ESI	Ion Polarity	Positive	Set Nebulizer	2.0 Bar
Focus	Not active	Set Capillary	4000 V	Set Dry Heater	230 °C
Scan Begin	120 m/z	Set End Plate Offset	-500 V	Set Dry Gas	6.0 l/min
Scan End	1500 m/z	Set Collision Cell RF	300.0 Vpp	Set Divert Valve	Waste

+MS, 2.0-2.1min #(117-122)

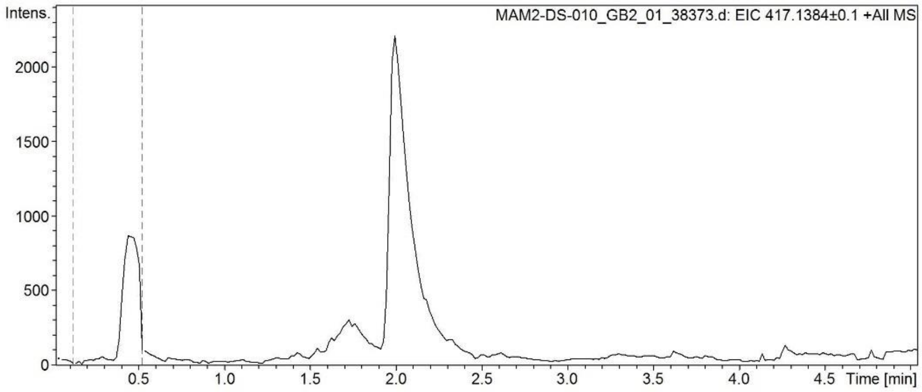

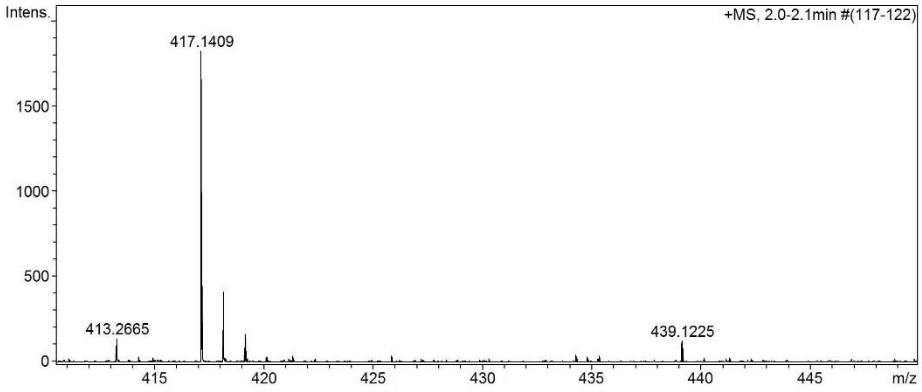

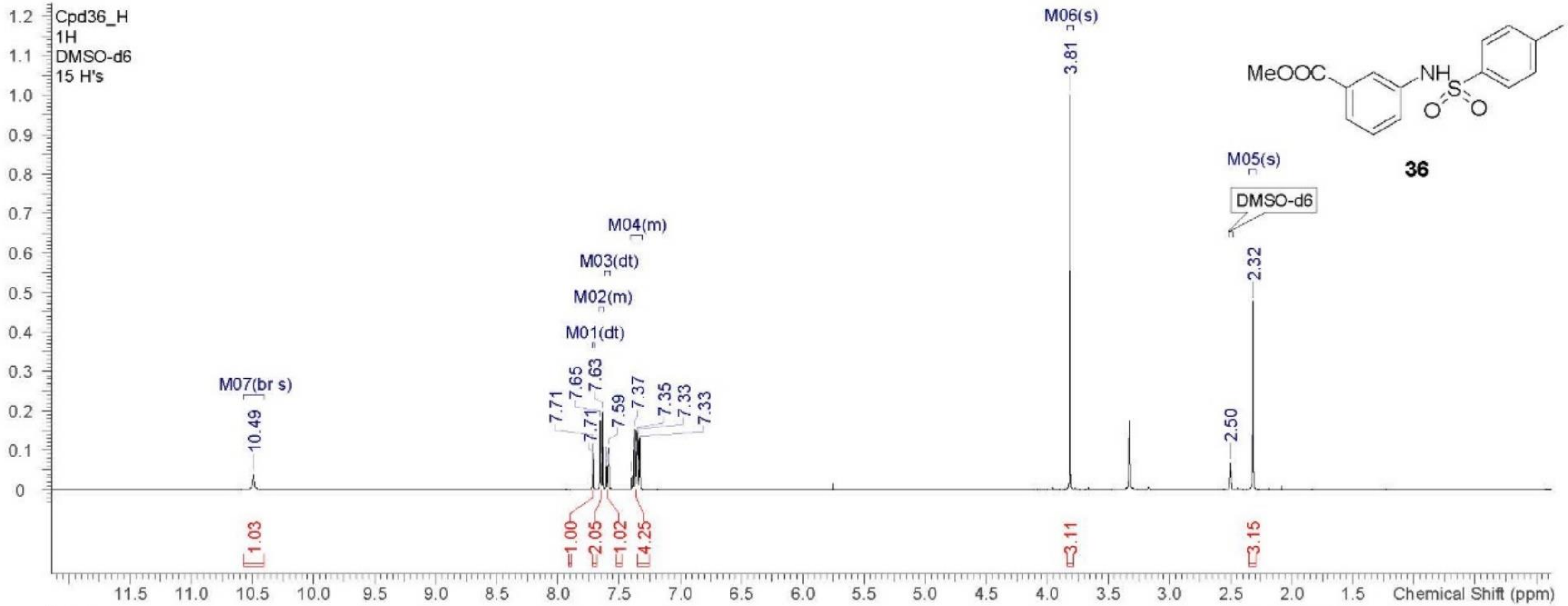
Cpd36_H
1H
DMSO-d6
15 H's
MeOOC
NH
S
O
O
36
M07(br s)
10.49
M01(dt)
M02(m)
M03(dt)
M04(m)
7.71
7.71
7.65
7.63
7.59
7.37
7.35
7.33
7.33
M06(s)
3.81
M05(s)
DMSO-d6
2.50
2.32
1.03
1.00
2.05
1.02
4.25
3.11
3.15
Chemical Shift (ppm)

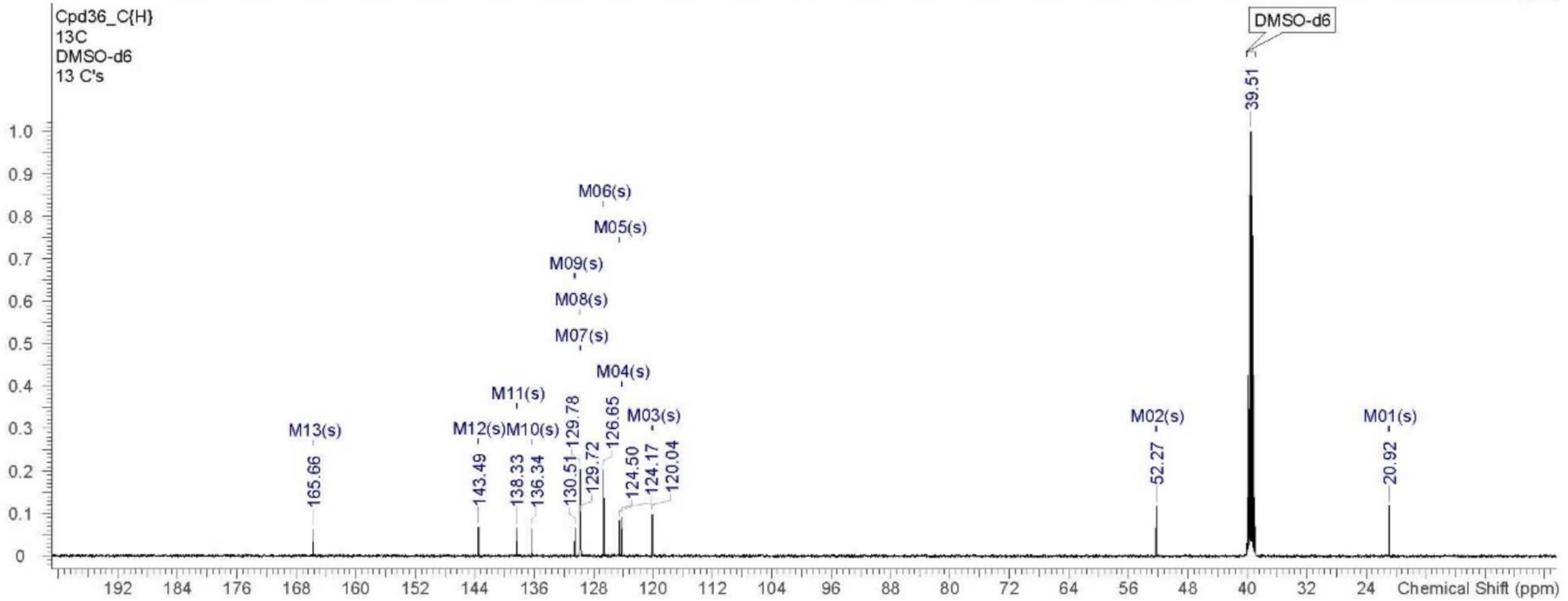
Cpd36_C{H}
13C
DMSO-d6
13 C's
M13(s)
165.66
M12(s)
143.49
M11(s)
138.33
M10(s)
136.34
M09(s)
M08(s)
M07(s)
130.51
129.78
129.72
M06(s)
M05(s)
M04(s)
126.65
124.50
124.17
M03(s)
120.04
M02(s)
52.27
DMSO-d6
39.51
M01(s)
20.92
Chemical Shift (ppm)

Top 5 Peak Report - UV

Sample ID: MAM7-065 LRMS
Group: Baud, M
Acquisition Date: 27/01/2021 19:21:39
Experiment: BLUE ESIPOSNEG C18 5 min
Filename: MAM7_065_LRMS_Michael_McCoy__Baud_M__88838.pdf

Submitter: Michael McCoy
Project: RP LC C18 custom
Instrument: Blue RP UHPLC-MS (B30:1023)

Absorbance, NL 1.338E06

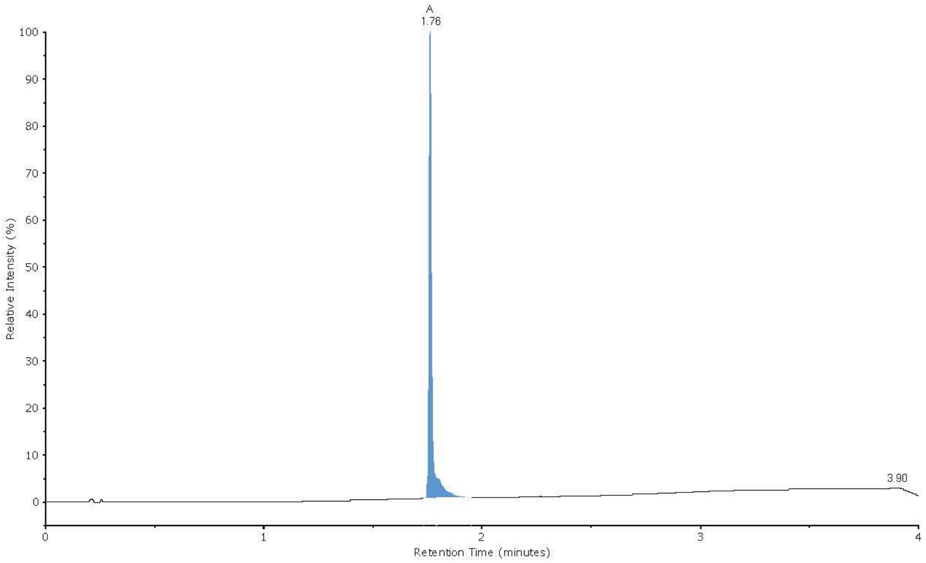

	RT Mins	Height	Height %	Area	Absolute Area %	Relative Area %
A	1.76	1300984	100.00	1117290	89.04	100.00
B	1.80	54251	4.17	137530	10.96	12.31

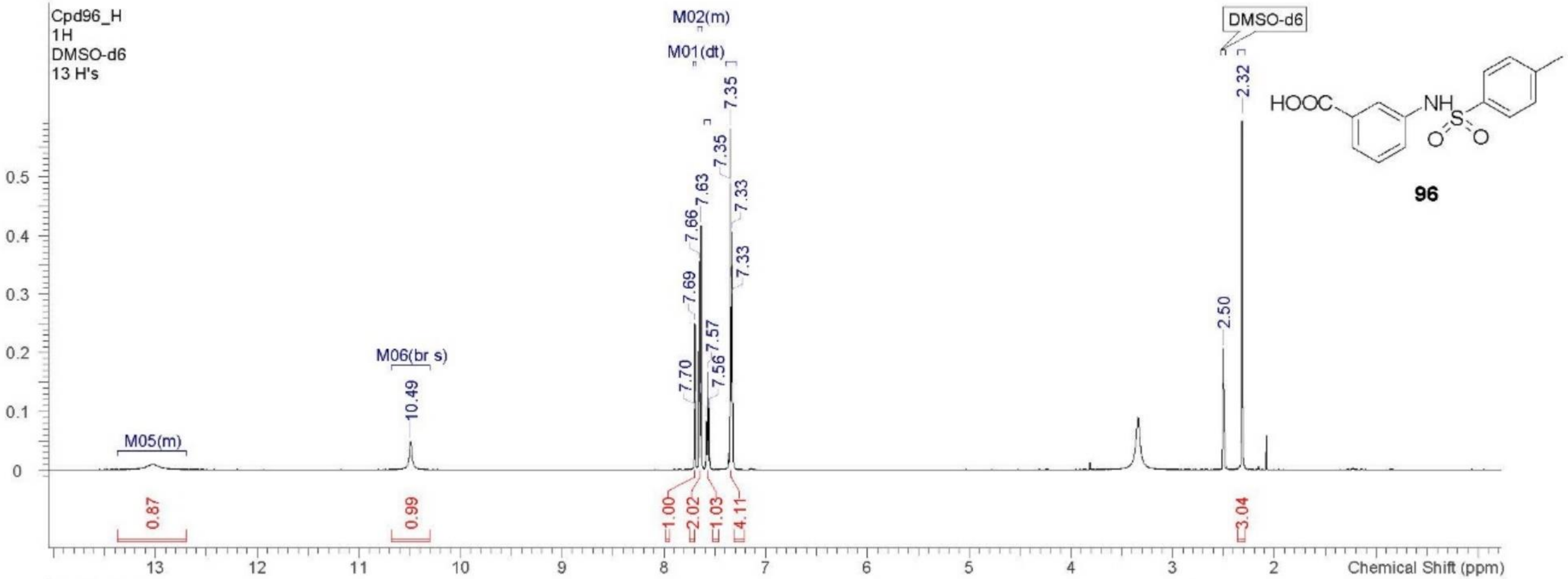
Cpd96_H
1H
DMSO-d6
13 H's
M02(m)
M01(dt)
M05(m)
M06(br s)
DMSO-d6
HOOC
NH
S
O
O
96
10.49
7.70
7.69
7.66
7.63
7.57
7.56
7.35
7.35
7.33
7.33
2.50
2.32
0.87
0.99
1.00
2.02
1.03
4.11
3.04
Chemical Shift (ppm)

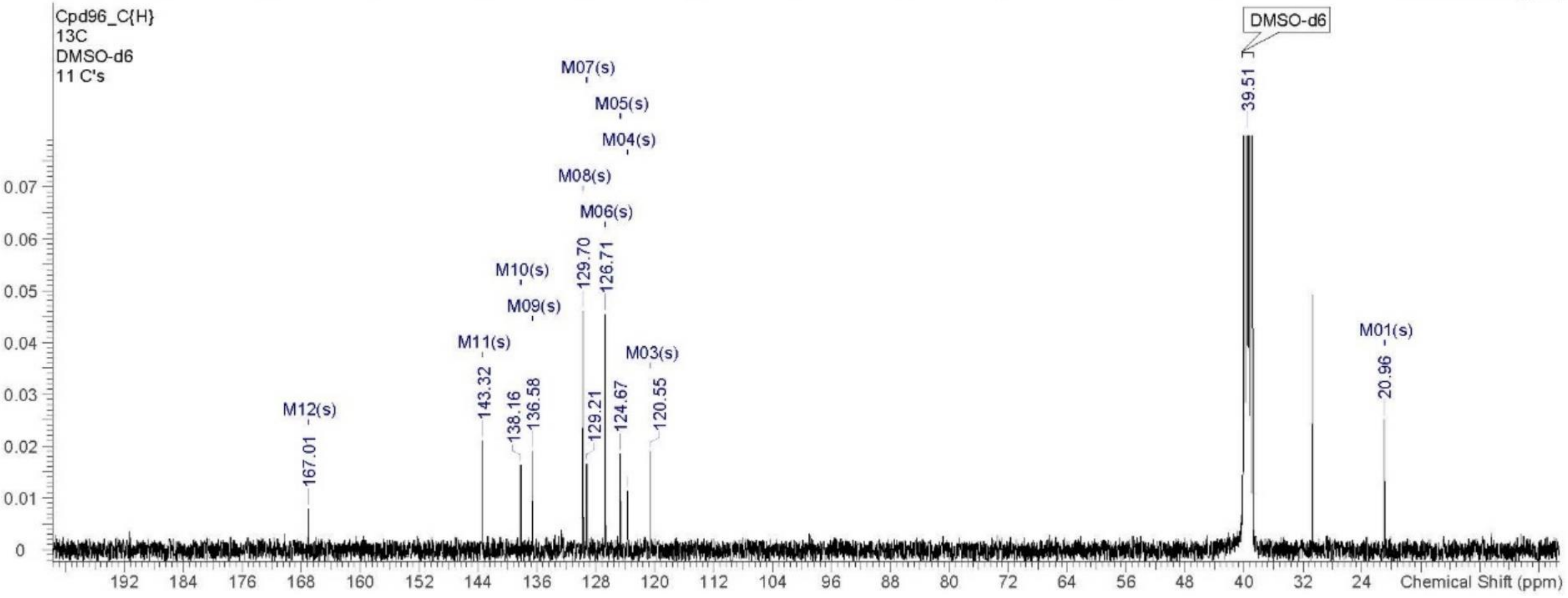
Cpd96_C{H}
13C
DMSO-d6
11 C's
DMSO-d6
M12(s)
M11(s)
M10(s)
M09(s)
M08(s)
M07(s)
M06(s)
M05(s)
M04(s)
M03(s)
M01(s)
167.01
143.32
138.16
136.58
129.70
129.21
126.71
124.67
120.55
39.51
20.96
Chemical Shift (ppm)

Top 5 Peak Report - UV

Sample ID: MAM7-076 LRMS
Group: Baud, M
Acquisition Date: 04/02/2021 15:14:58
Experiment: BLUE ESIPOSNEG C18 5 min
Filename: MAM7_076_LRMS_Michael_McCoy__Baud_M__90299.pdf

Submitter: Michael McCoy
Project: RP LC C18 custom
Instrument: Blue RP UHPLC-MS (B30:1023)

Absorbance, NL 2.169E06

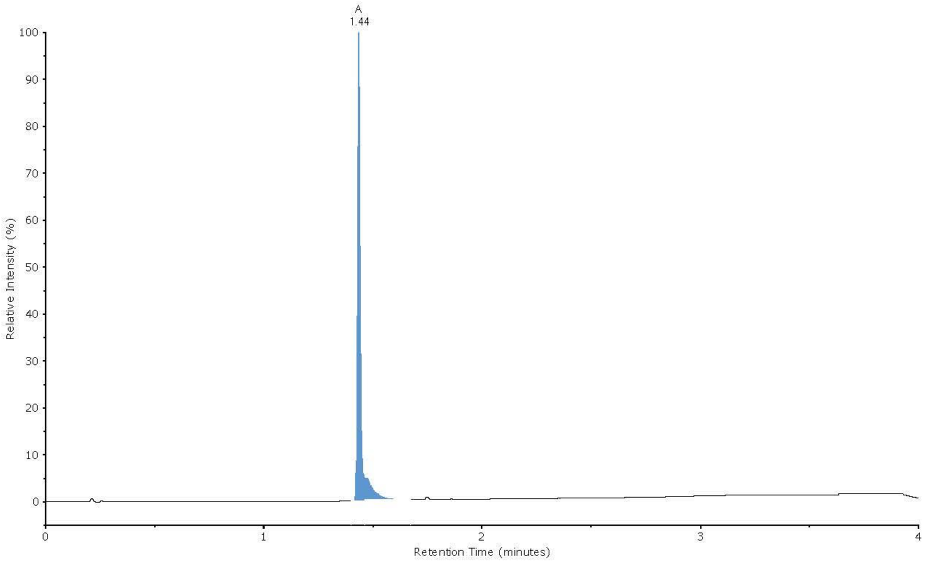

	RT Mins	Height	Height %	Area	Absolute Area %	Relative Area %
A	1.44	2113911	100.00	2074857	88.13	100.00
B	1.47	103627	4.90	279368	11.87	13.46

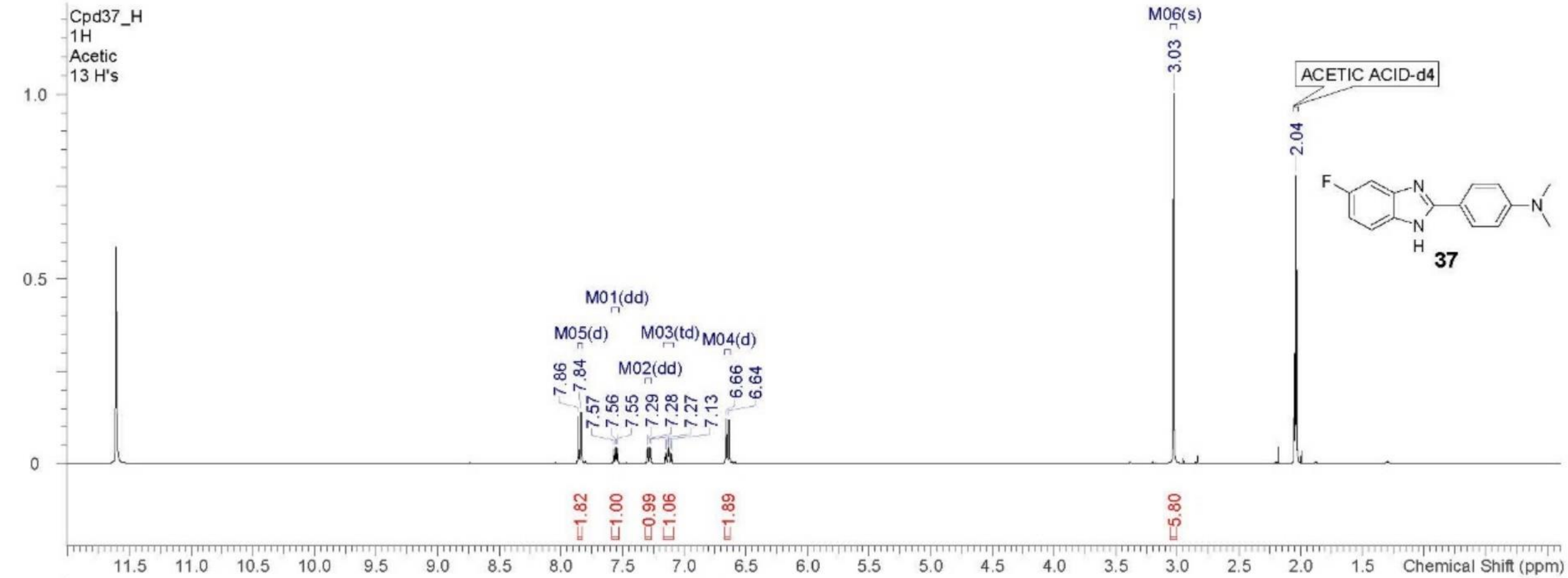
Cpd37_H
1H
Acetic
13 H's
M06(s)
3.03
ACETIC ACID-d4
2.04
F
N
N
H
N
37
M01(dd)
M05(d)
M03(td)
M04(d)
M02(dd)
7.86
7.84
7.57
7.56
7.55
7.29
7.28
7.27
7.13
6.66
6.64
1.82
1.00
0.99
1.06
1.89
5.80
Chemical Shift (ppm)

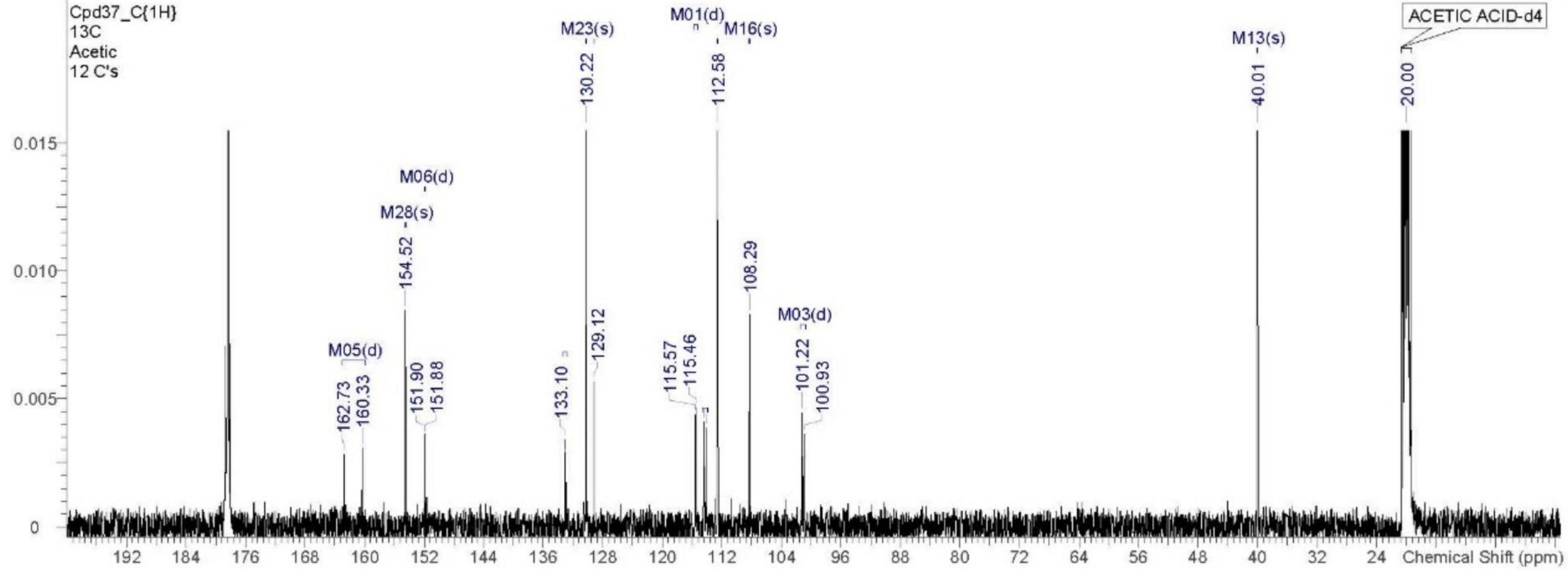
Cpd37_C{1H}
13C
Acetic
12 C's
M23(s)
130.22
M01(d)
M16(s)
112.58
M13(s)
40.01
ACETIC ACID-d4
20.00
M06(d)
M28(s)
154.52
108.29
M03(d)
M05(d)
162.73
160.33
151.90
151.88
133.10
129.12
115.57
115.46
101.22
100.93
Chemical Shift (ppm)

Chemistry - maXis HPLC-ESI Accurate Mass Report

Analysis Info

Analysis Name	D:\Data\Chemistry\2019\Dec\MAM3-033_RA7_01_39588.d	Acquisition Date	09/12/2019 16:09:35
Method	soton lcms pos 120 to 1500.m	Operator	MSWEB@SOTON.AC.UK
Sample Name	MAM3-033	Instrument / Ser#	maXis 17
Comment	Analyst: JMH		

Acquisition Parameter

Source Type	ESI	Ion Polarity	Positive	Set Nebulizer	2.0 Bar
Focus	Not active	Set Capillary	4000 V	Set Dry Heater	230 °C
Scan Begin	120 m/z	Set End Plate Offset	-500 V	Set Dry Gas	6.0 l/min
Scan End	1500 m/z	Set Collision Cell RF	300.0 Vpp	Set Divert Valve	Waste

Cmpd 1, 1.8 min

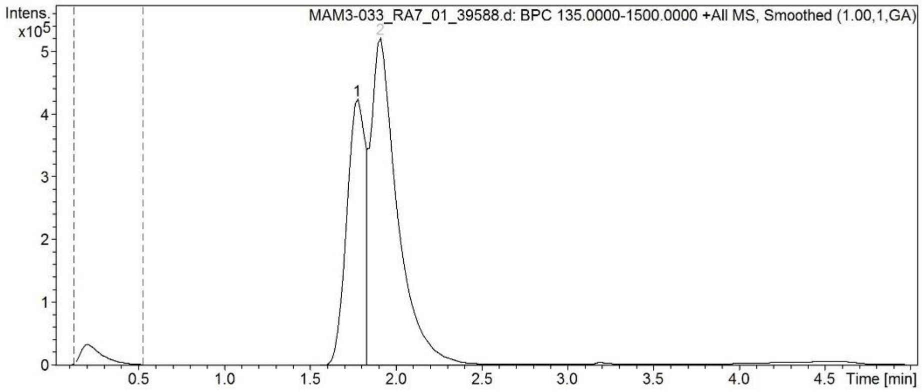

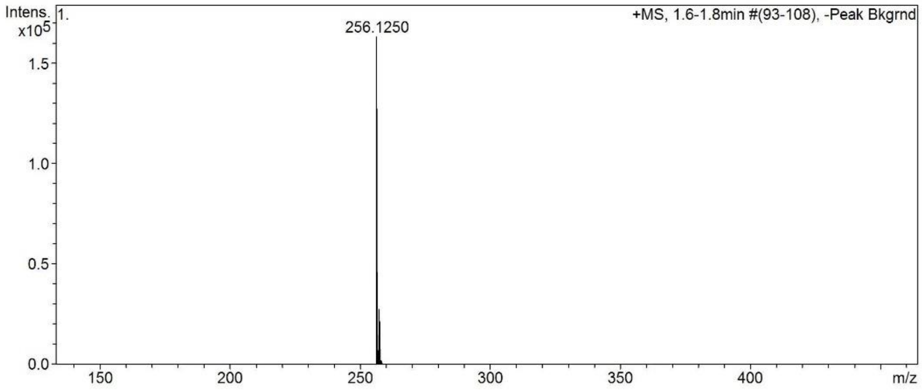

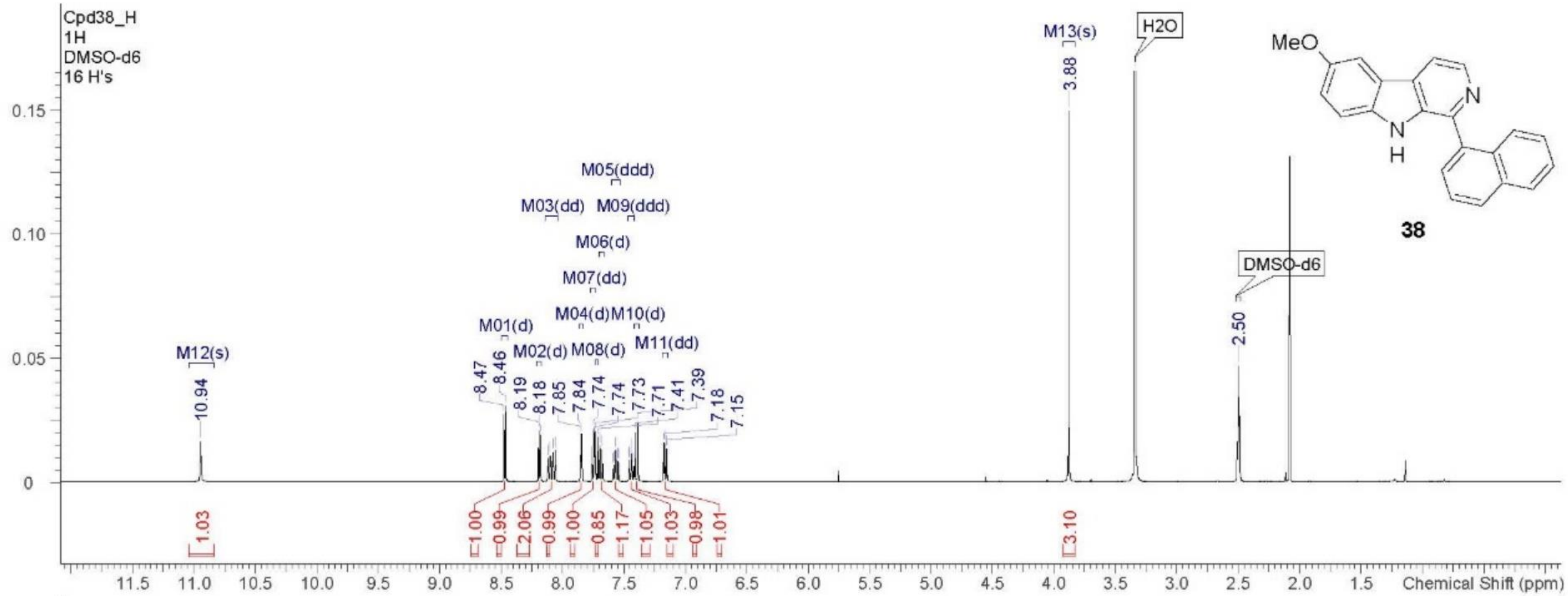
Cpd38_H
1H
DMSO-d6
16 H's
MeO
N
N
H
38
H2O
DMSO-d6
M13(s)
3.88
M12(s)
10.94
M01(d)
M02(d)
M03(dd)
M04(d)
M05(ddd)
M06(d)
M07(dd)
M08(d)
M09(ddd)
M10(d)
M11(dd)
8.47
8.46
8.19
8.18
7.85
7.84
7.74
7.74
7.73
7.71
7.41
7.39
7.18
7.15
2.50
1.03
1.00
0.99
2.06
0.99
1.00
0.85
1.17
1.05
1.03
0.98
1.01
3.10
Chemical Shift (ppm)

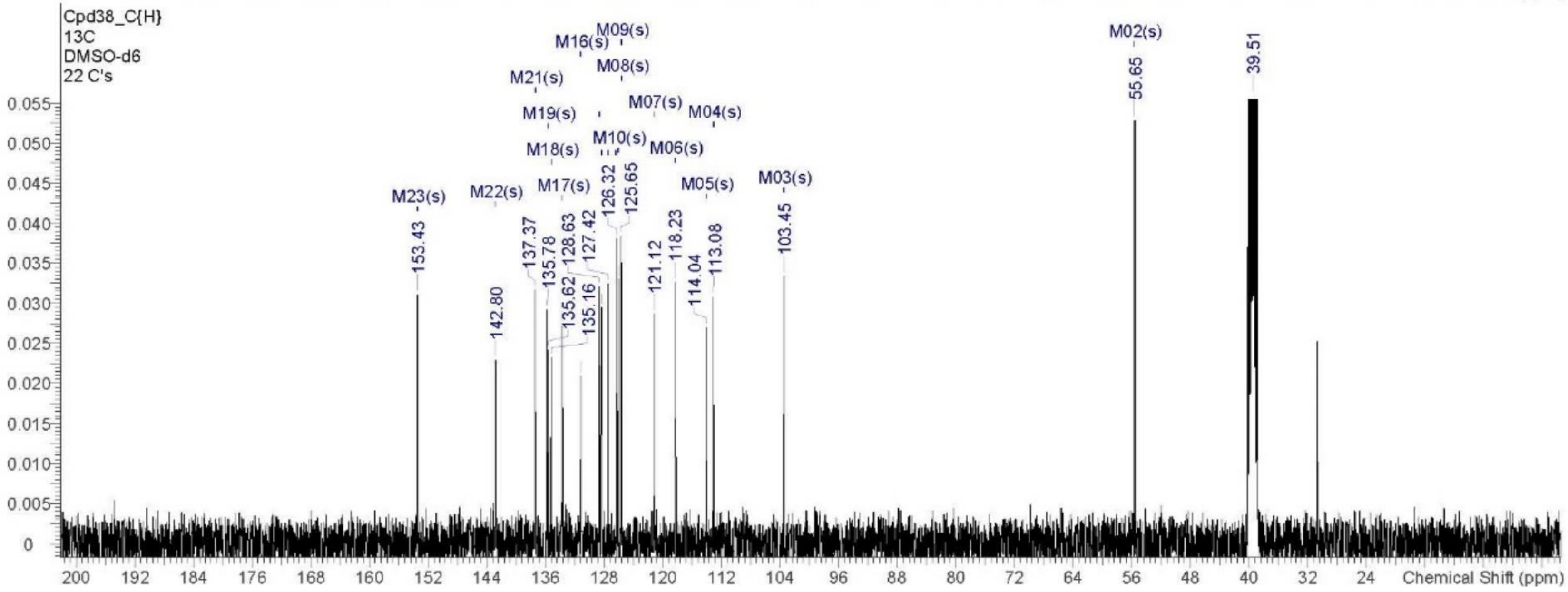
Cpd38_C{H}
13C
DMSO-d6
22 C's
M23(s)
153.43
M22(s)
142.80
M21(s)
M19(s)
M18(s)
M17(s)
M16(s)
M09(s)
M08(s)
M10(s)
M07(s)
M06(s)
M05(s)
M04(s)
M03(s)
M02(s)
137.37
135.78
135.62
135.16
128.63
127.42
126.32
125.65
121.12
118.23
114.04
113.08
103.45
55.65
39.51
Chemical Shift (ppm)

Top 5 Peak Report - UV

Sample ID: MAM3-050 Column
Group: Baud, M
Acquisition Date: 02/10/2019 18:22:05
Experiment: BLUE ESIPOSNEG C18 5 min
Filename: MAM3_050_Column_Michael_McCoy__Baud_M__69801.pdf

Submitter: Michael McCoy
Project: RP LC C18 custom
Instrument: Blue RP UHPLC-MS (B30:1023)

Absorbance, NL 1.147E06

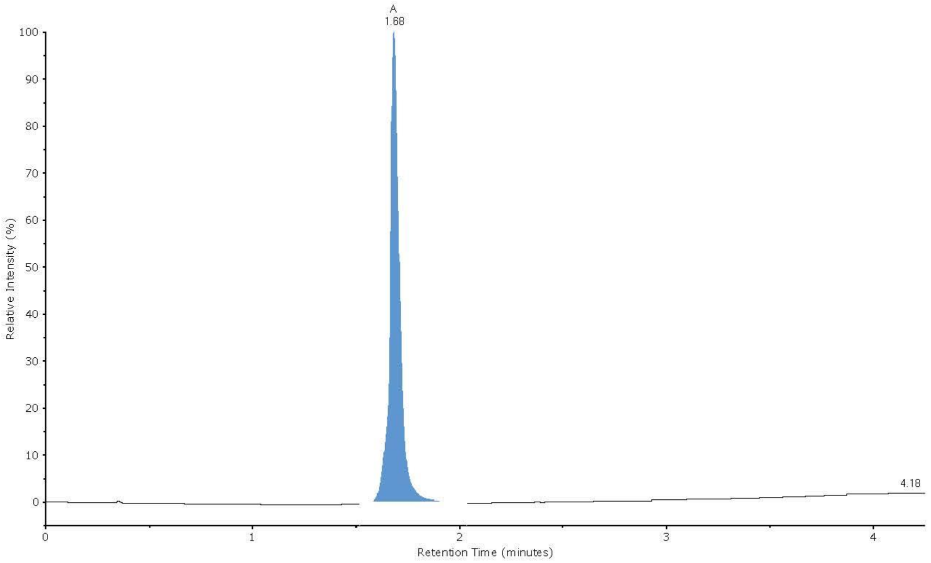

	RT Mins	Height	Height %	Area	Absolute Area %	Relative Area %
A	1.68	1152155	100.00	3784359	100.00	100.00

References

1. Todorovic, N.; Giacomelli, A.; Hassell, J. A.; Frampton, C. S.; Capretta, A., Microwave-assisted synthesis of 3-aryl-pyrimido[5,4-e][1,2,4]triazine-5,7(1H,6H)-dione libraries: derivatives of toxoflavin. *Tetrahedron Letters* **2010,** *51* (46), 6037-6040.

2. Mao, Y.; Tian, W.; Huang, Z.; An, J., Convenient Synthesis of Toxoflavin that Targets β-Catenin/Tcf4 Signaling Activities. *Journal of Heterocyclic Chemistry* **2014,** *51* (3), 594-597.

3. Mao, Y.; Lin, N.; Tian, W.; Han, X.; Han, X.; Huang, Z.; An, J., Design, Synthesis, and Biological Evaluation of New Diaminoquinazolines as β-Catenin/Tcf4 Pathway Inhibitors. *Journal of Medicinal Chemistry* **2012,** *55* (3), 1346-1359.
